# An Improved Theory for Designing and Numerically Calibrating Circular Touch Mode Capacitive Pressure Sensors

**DOI:** 10.3390/s24030907

**Published:** 2024-01-30

**Authors:** Xiao-Ting He, Xin Wang, Fei-Yan Li, Jun-Yi Sun

**Affiliations:** 1 School of Civil Engineering, Chongqing University, Chongqing 400045, China; 202216131258t@stu.cqu.edu.cn (X.W.); 202116131224t@cqu.edu.cn (F.-Y.L.); sunjunyi@cqu.edu.cn (J.-Y.S.); 2 Key Laboratory of New Technology for Construction of Cities in Mountain Area (Chongqing University), Ministry of Education, Chongqing 400045, China

**Keywords:** pressure sensor, capacitive sensor, touch mode of operation, circular conductive membrane, analytical solution, numerical calibration

## Abstract

The design, especially the numerical calibration, of a circular touch mode capacitive pressure sensor is highly dependent on the accuracy of the analytical solution of the contact problem between the circular conductive membrane and the rigid plate of the sensor. In this paper, the plate/membrane contact problem is reformulated using a more accurate in-plane equilibrium equation, and a new and more accurate analytical solution is presented. On this basis, the design and numerical calibration theory for circular touch mode capacitive pressure sensors has been greatly improved and perfected. The analytical relationships of pressure and capacitance are numerically calculated using the new and previous analytical solutions, and the gradually increasing difference between the two numerical calculation results with the gradual increase in the applied pressure is graphically shown. How to use analytical solutions and analytical relationships to design and numerically calibrate a circular touch mode capacitive pressure sensor with a specified pressure detecting range is illustrated in detail. The effect of changing design parameters on capacitance–pressure analytical relationships is comprehensively investigated; thus, the direction of changing design parameters to meet the required or desired range of pressure or capacitance is clarified.

## 1. Introduction

Membranes have a wide range of applications in engineering, technology and other fields, such as space engineering [1], wastewater treatment [2], bionic structure of tympanic membranes [3] and so on. Many membranes have the ability to exhibit large elastic deflections under transverse loading [4,5,6,7,8,9,10], which provides the possibility of designing and developing deflection-based devices [11,12,13,14,15,16,17,18,19]. For instance, the circular capacitive pressure sensor addressed here is such a deflection-based device, which is a pressure sensor using a circular conductive membrane as the sensitive element and a variable capacitor as the sensing element. The circular conductive membrane, which works as the movable electrode plate of the variable capacitor, elastically deflects toward the fixed electrode plate of the variable capacitor under pressure, resulting in the capacitance change in the variable capacitor. So, the testing principle of such a capacitive pressure sensor is to detect the applied pressure by measuring the capacitance change under the applied pressure, where the sensitive element (the circular conductive membrane) converts the pressure to be detected to the membrane deflection, and the sensing element (the variable capacitor) converts the membrane deflection to the capacitance. Such sensors usually operate in non-touch (or normal) mode or touch mode, and can be embedded or packaged for use, for example, embedded in industrial structures such as tires, or packaged as conventional sensors for industrial field applications. Considering that the touch mode of operation has many advantages over the non-touch mode of operation, this paper is devoted to the improvement of the design and numerical calibration theory for circular touch mode capacitive pressure sensors.

**Figure 1:**
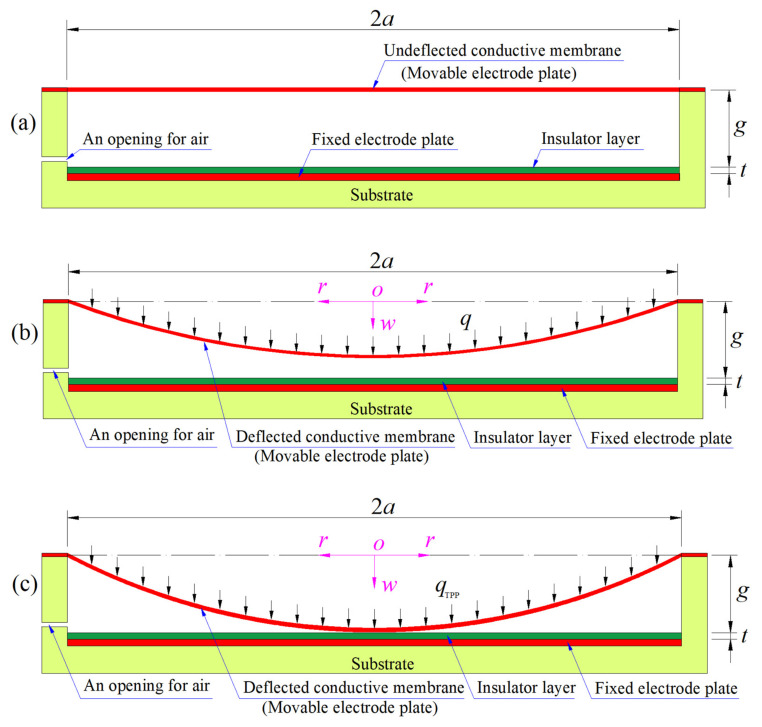

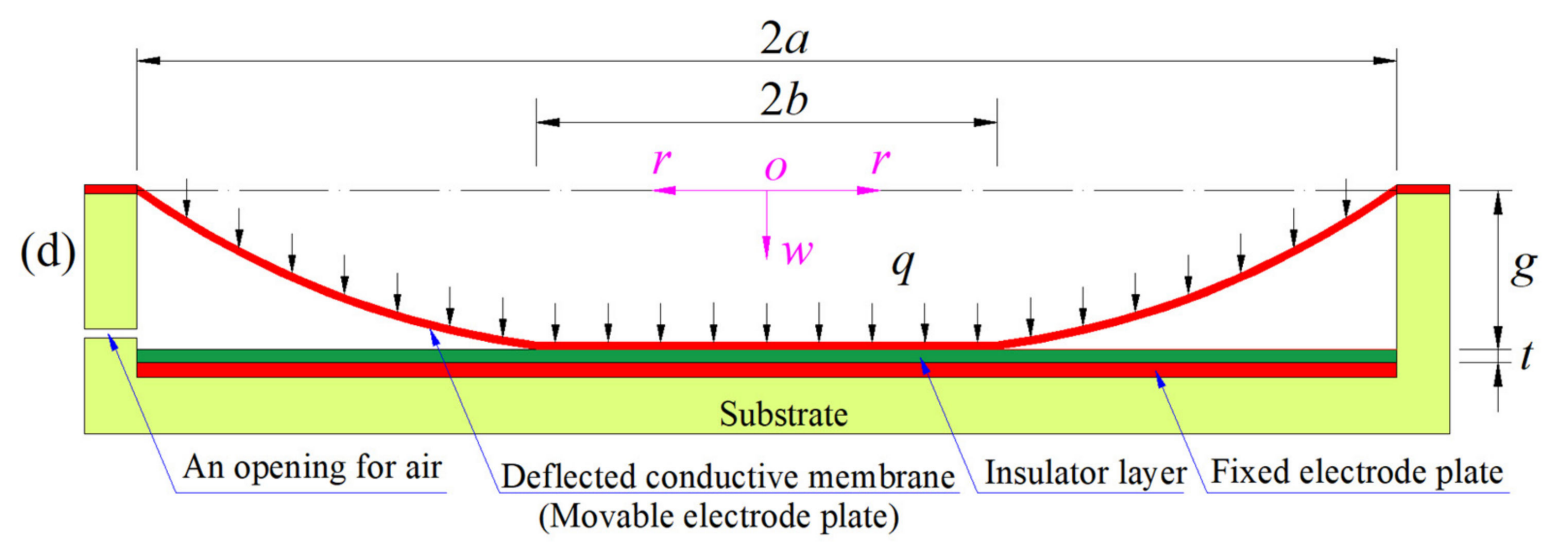
Sketch of a circular capacitive pressure sensor from non-touch mode of operation to touch mode of operation: (**a**) initial state, (**b**) non-touch mode of operation, (**c**) critical state between non-touch mode of operation and touch mode of operation, and (**d**) touch mode of operation.

The key element of a circular capacitive pressure sensor is a circular variable capacitor using a circular conductive membrane and a thin plate as the movable and fixed electrode plates, as shown in Figure 1, where *a* is the radius of the initially flat circular conductive membrane; *g* is the initially parallel gap between the initially flat circular conductive membrane and the insulator layer; *t* is the thickness of the insulator layer; the dash–dotted line represents the geometric middle plane of the initially flat circular conductive membrane; *q* is the pressure to be detected; $q_{\mathrm{TPP}}$ is the touch point pressure when the circular conductive membrane under the pressure *q* just touches the insulator layer; *b* is the radius of the circular contact area between the deflected conductive membrane and the insulator layer; *o* denotes the origin of the introduced cylindrical coordinate system (*r*, *φ*, *w*); *r* is the radial coordinate; *φ* is the angle coordinate but is not represented due to the characteristics of axial symmetry and *w* is the transverse coordinate that also denotes the deflection in the circular conductive membrane under the pressure *q*.

Before application of the pressure *q*, as shown in Figure 1a, the circular conductive membrane is initially flat (undeflected), the circular conductive thin plate is fixed on the substrate and coated with a thin layer of insulator and the initially parallel gap between the initially flat circular conductive membrane and the insulator layer is filled with air. On application of the pressure *q*, as shown in Figure 1b, the initially flat circular conductive membrane elastically deflects towards the insulator layer and is thus known as a movable electrode plate, while the circular conductive thin plate fixed on the substrate is known as a fixed electrode plate. When the pressure *q* reaches the touch point pressure $q_{\mathrm{TPP}}$ , the deflected conductive membrane just touches the insulator layer, as shown in Figure 1c. 

Therefore, before the pressure *q* reaches the touch point pressure $q_{\mathrm{TPP}}$ , the total capacitor between the movable and fixed electrode plates can be regarded as one consisting of two capacitors in series. The first capacitor is the one between the conductive membrane and the insulator layer, and the second capacitor is the one between the insulator layer and the conductive thin plate. Obviously, the application of the pressure *q* only causes a change in the capacitance of the first capacitor and does not affect the capacitance of the second capacitor. So, the first capacitor is known as a variable capacitor and the second capacitor is known as a fixed capacitor. The first capacitor changes from the parallel plate capacitor before application of the pressure *q* to the non-parallel plate capacitor after application of the pressure *q* (from the initially parallel gap *g* to the non-parallel gap *g*–*w*(*r*), see Figure 1a,b), and the second capacitor always remains as a parallel plate capacitor (whose parallel gap is the thickness *t* of the insulator layer). Of course, the total capacitor between the movable and fixed electrode plates is also a variable capacitor.

After the pressure *q* exceeds the touch point pressure $q_{\mathrm{TPP}}$ , that is, when $q>q_{\mathrm{TPP}}$ , a circular contact area will be formed between the deflected conductive membrane and the insulator layer, and the radius *b* of the circular contact area will gradually increase as the pressure *q* further increases, as shown in Figure 1d. At this time, the total capacitor between the movable and fixed electrode plates (which will be denoted by C in Section 3) can be regarded as one consisting of two capacitors in parallel (which will be denoted by C_1_ and C_2_ in Section 3). C_1_ refers to the parallel plate capacitor in the contact area of *0* ≤ *r* ≤ *b* (the parallel gap between its two electrode plates is equal to the thickness *t* of the insulator layer, see Figure 1d), and is a variable capacitor due to the gradually increasing *b*. C_2_ refers to the non-parallel plate capacitor in the non-contact area of *b* ≤ *r* ≤ *a*, and can be regarded as one consisting of two capacitors in series (which will be denoted by C_3_ and C_4_ in Section 3). C_3_ refers to the parallel plate capacitor in the non-contact area of *b* ≤ *r* ≤ *a* (the parallel gap between its two electrode plates is equal to the thickness *t* of the insulator layer, see Figure 1d) and is a variable capacitor due to the gradually increasing *b*. C_4_ refers to the non-parallel plate capacitor in the non-contact area of *b* ≤ *r* ≤ *a* (the non-parallel gap between its two electrode plates is equal to *g–w*(*r*), see Figure 1d) and is also a variable capacitor due to the gradually increasing *b* as well as the non-parallel gap *g–w*(*r*) varying with the applied pressure *q*. 

The circular capacitive pressure sensor in Figure 1 is said to operate in touch mode when $q>q_{\mathrm{TPP}}$ , to operate in non-touch (or normal) mode when $q<q_{\mathrm{TPP}}$ , to be in a critical state when $q\;=q_{\mathrm{TPP}}$ , and to be in an initial state when q =0 , corresponding to Figure 1a–d, respectively. 

Capacitive pressure sensors are less sensitive to side stress and other environmental effects, and have high sensitivity, robust structure and no turn-on temperature drift [20,21]. A capacitive pressure sensor is called a non-touch (or normal) mode capacitive pressure sensor if it operates in non-touch (or normal) mode, and is called a touch mode capacitive pressure sensor if it operates in touch mode. Obviously, since the substrate can directly bear the pressure *q* applied in the plate/membrane contact area of *0* ≤ *r* ≤ *b* (see Figure 1d), the touch mode capacitive pressure sensor has larger overload protection, in comparison with the non-touch mode capacitive pressure sensor. This implies that the pressure range when a capacitive pressure sensor operates in touch mode is much wider than that when this capacitive pressure sensor operates in non-touch (or normal) mode. Therefore, the touch mode capacitive pressure sensors can show better performance, especially in industrial applications. In the specific design and fabrication of a capacitive pressure sensor, it is very important to accurately understand the stress, strain and displacement in the circular conductive membrane under the pressure *q*. Therefore, it is often necessary to analytically solve the elastic behavior of the circular conductive membrane under the pressure *q*.

On the other hand, it can be seen from Figure 1b,d that under the pressure *q*, the circular conductive membrane is in a state of free deflection when the sensor operates in non-touch (or normal) mode, and when the sensor operates in touch mode, it is in a state of limited maximum deflection. The former is a large deflection problem of a circular membrane under transverse uniform loading (the well-known Föppl–Hencky membrane problem), while the latter is an axisymmetric contact problem between a deflected circular membrane and a rigid plate (which is usually called the plate/membrane contact problem for short). In comparison with the analytical solution to the plate/membrane contact problem, the well-known Föppl–Hencky membrane problem is much easier to be analytically solved. 

In fact, the Föppl–Hencky membrane problem has been very well solved analytically [22,23,24,25], but the analytical solution of the plate/membrane contact problem still needs to be further improved. Xu and Liechti solved this plate/membrane contact problem based on the assumptions of an equi-biaxial constant stress state and small rotation angle of the membrane [26]. Wang et al. presented a closed-form solution of this plate/membrane contact problem by giving up the assumption of equi-biaxial constant stress state for the first time [27]. Lian et al. also presented a closed-form solution of this plate/membrane contact problem [28], where the equi-biaxial constant stress state assumption was given up, and the out-of-plane equilibrium equation used was established by giving up the small rotation angle assumption of the membrane. Li et al. presented a more refined closed-form solution of this plate/membrane contact problem [29], where the out-of-plane equilibrium equation and geometric equations used were established by giving up the small rotation angle assumption of the membrane, except for giving up the equi-biaxial constant stress state assumption. However, the in-plane equilibrium equation used in [26,27,28,29] is the classic one, which does not take into account the contribution of deflection to in-plane equilibrium at all and is only applicable to plane-stretched or compressed membranes and not to large deflection membranes. In other words, the classic in-plane equilibrium equation used in [26,27,28,29] is only applicable to the plane-stretched membrane in the plate/membrane contact area of *0* ≤ *r* ≤ *b* and not to the large deflection membrane in the plate/membrane non-contact area of *b* ≤ *r* ≤ *a* (see Figure 1d). So, in [26,27,28,29], the use of the classic in-plane equilibrium equation inevitably introduces calculation errors. In this paper, the plate/membrane contact problem is reformulated using a more accurate in-plane equilibrium equation which fully takes into account the contribution of deflection to the in-plane equilibrium [25], resulting in a new and more accurate analytical solution of the problem. On this basis, the design and numerical calibration theory for circular touch mode capacitive pressure sensors have been greatly improved and perfected. 

This paper is organized in the order of, first, the sensitive element, then the sensing element, then the results and discussion, and finally the concluding remarks. In the following section, depending on the magnitude of the applied pressure *q* (see Figure 1), the elastic behavior of the sensitive element (the circular conductive membrane) is reduced to a large deflection problem (Figure 1b) and a plate/membrane contact problem (Figure 1d), respectively. The new and more accurate analytical solution of the plate/membrane contact problem in Figure 1d is presented, where the stress solution is used for the strength design of the sensitive element, and the deflection solution is used for determining the total capacitance of the sensing element (the capacitor between the movable and fixed electrode plates, see Figure 1d). In Section 3, based on the newly presented deflection solution, the analytical relationship between the total capacitance and the applied pressure *q* is derived and discussed. In Section 4, an example is first given to illustrate how to use the analytical solutions of the large deflection problem and plate/membrane contact problem as well as the pressure–capacitance analytical relationship to design and numerically calibrate a circular touch mode capacitive pressure sensor with a specified pressure detecting range. Then, the analytical relationships of the capacitance as input and the applied pressure as output are calculated using the newly and previously presented analytical solutions, and are compared to show the rapidly increasing difference between the two calculated results with the increase in the applied pressure. Finally, the effect of changing design parameters on input capacitance–output pressure relationships is comprehensively investigated, including changing the radius *a*, thickness *h*, Poisson’s ratio *v* and Young’s modulus of elasticity *E* of the circular conductive membrane, as well as the thickness *t* of the insulator layer and the initially parallel gap *g* between the initially flat circular conductive membrane and the insulator layer. Concluding remarks are given in Section 5.

The contribution of this study mainly lies in the following two aspects. One is purely a mechanical contribution: using a more accurate in-plane equilibrium equation, a new and more accurate analytical solution of the plate/membrane contact problem is presented for the first time. The other aspect is the technical contribution: based on the newly presented analytical solution, the design and numerical calibration theory for circular touch mode capacitive pressure sensors has been greatly improved and perfected on the basis of the existing theory. 

## 2. More Refined Analytical Solution to the Sensitive Element of the Sensor

The circular conductive membrane, as the sensitive element of the circular capacitive pressure sensor, produces axisymmetric deformation with a large deflection in response to the applied pressure *q*, as shown in Figure 1. Before the pressure *q* reaches the touch point pressure $q_{\mathrm{TPP}}$ (see Figure 1c), the initially flat circular conductive membrane (see Figure 1a) is in a state of free deflection, as shown in Figure 1b, which is usually called the large deflection problem of circular membranes under uniformly distributed transverse loads. This large deflection problem of circular membranes was dealt with originally by Föppl [30] and Hencky [31], and is usually called the Föppl–Hencky membrane problem. After the pressure *q* reaches the touch point pressure $q_{\mathrm{TPP}}$ , the deflected circular conductive membrane is in a state of limited maximum deflection, as shown in Figure 1d, which is known as an axisymmetric contact problem between a deflected circular membrane and a rigid plate, or the plate/membrane contact problem for short. 

The plate/membrane contact problem has many potential applications such as the membrane/substrate delamination [32,33,34], adhesion [35,36,37,38,39,40] and especially capacitive pressure sensors [41,42,43,44,45,46,47,48,49,50,51]. However, the plate/membrane contact problem involves both the plane-stretched membrane in the plate/membrane contact area of *0* ≤ *r* ≤ *b* and the large deflection membrane in the plate/membrane non-contact area of *b* ≤ *r* ≤ *a* (see Figure 1d). But the existing analytical solutions of the plate/membrane contact problem [26,27,28,29] are all obtained using the classic in-plane equilibrium equation that is only applicable to the plane-stretched membrane problems, which inevitably introduces calculation errors. In this paper, this plate/membrane contact problem is further analytically solved by giving up the equi-biaxial constant stress state assumption and using more accurate out-of-plane and in-plane equilibrium equations and geometric equations, and a new and more refined closed-form solution is presented, which is detailed as follows.

Suppose that the circular conductive membrane in Figure 1 has Young’s modulus *E*, Poisson’s ratio *ν*, thickness *h* and radius *a*. After the pressure *q* reaches the touch point pressure $q_{\mathrm{TPP}}$ , the circular conductive membrane comes into axisymmetric contact with the insulator layer, resulting in a contact radius *b*. The maximum deflection *w*_m_ of the circular membrane is always equal to the initially parallel gap *g*, that is, *w*(*r*) ≡ *g* when *r* ≤ *b* (see Figure 1d). Throughout the following formulation, it is assumed that the circular membrane always has a constant thickness *h* during its deflection. In this plate/membrane axisymmetric contact problem, the whole deflected circular membrane may be divided into two parts: one is a circular plate/membrane contact area with a contact radius *b* and the other part is an annular plate/membrane non-contact area with an inner radius *b* and an outer radius *a*. In the plate/membrane contact area, the circular membrane only undergoes the in-plane axisymmetric stretching (only a plane problem), while in the plate/membrane non-contact area, the annular membrane undergoes the out-of-plane axisymmetric deflection. The stress, strain and displacement of the membrane should be continuous at the connecting ring between the annular region and the circular region, i.e., at *r* = *b* (see Figure 1d). Such a continuity condition will be used as conditions for determining special solutions.

**Figure 2:**
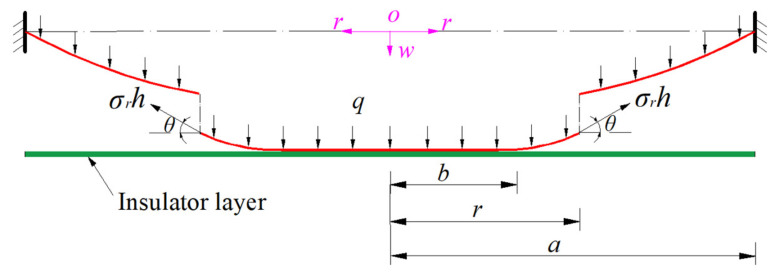
A free body with radius *r* (*b* ≤ *r* ≤ *a*) taken from the circular conductive membrane in contact with the insulator layer in Figure 1d.

A free body of radius *r* (*b* ≤ *r* ≤ *a*) is assumed to be taken from the circular conductive membrane in contact with the insulator layer in Figure 1d, as shown in Figure 2, to study its static problem of equilibrium, where *σ_r_* is the radial stress at *r* and *θ* is the meridional rotation angle of the deflected membrane at *r*.

In the vertical direction perpendicular to the initially flat circular membrane (represented by the dash–dotted line in Figure 2), the vertical forces are *πr*^2^*q* (the total force of the uniformly distributed transverse loads *q* within radius *r*), *πb*^2^*q* (the total reaction force from the rigid plate) and 2*πrσ_r_h*sin*θ* (the total vertical force produced by the membrane force *σ_r_h*), where *b* ≤ *r* ≤ *a*. Therefore, the out-of-plane equilibrium equation can be derived from the condition that the resultant force should be equal to zero in the vertical direction, i.e.,

(1) $$\pi r^{2}q-\pi b^{2}q-2\pi r\sigma_{r}h\sin\theta =0,$$

where

(2) $$\sin\theta =1/\sqrt{1+1/\tan^{2}\theta }=1/\sqrt{1+1/{\left(-dw/dr\right)}^{2}}.$$

Substituting Equation (2) into Equation (1) yields

(3) $$(r^{2}-b^{2})q\sqrt{1+1/{\left(dw/dr\right)}^{2}}=2r\sigma_{r}h.$$

The classic in-plane equilibrium equation does not take into account the contribution of deflection to in-plane equilibrium at all [22,26,27,28,29,31]. So, Li et al. modified the classic in-plane equilibrium equation, but presented only an in-plane equilibrium equation partly taking into account the contribution of deflection to in-plane equilibrium [4]. The in-plane equilibrium equation that fully takes into account the contribution of deflection to in-plane equilibrium was established by Sun et al. [25], and is given by

(4) $$\frac{d}{dr}[\frac{r\sigma_{r}}{\sqrt{1+{\left(-\frac{dw}{dr}\right)}^{2}}}]-\sigma_{t}\sqrt{1+{\left(-\frac{dw}{dr}\right)}^{2}}=0,$$

where *σ_t_* denotes the circumferential stress. 

Obviously, if the membrane is in the plane-stretched or compressed state, then the membrane is flat, the first derivative of the deflection *w*(*r*) is thus equal to zero, i.e., d*w*/d*r ≡* 0. Therefore, after substituting d*w*/d*r* = 0 into Equation (4), we can obtain the classic in-plane equilibrium equation in [22,26,27,28,29,31], that is, $d(r\sigma_{r})/dr-\sigma_{t}=0$ . So, the classic in-plane equilibrium equation used in [22,26,27,28,29,31] is only applicable to the membrane in the plate/membrane contact area of 0 ≤ *r* ≤ *b*, and not to the large deflection membrane in the plate/membrane non-contact area of *b* ≤ *r* ≤ *a* (see Figure 2).

In order to take into account the effect of deflection on geometric relationship, the geometric equations (the relationships between strain and displacement) have been modified from the classic one [22,31] into the following form [24]

(5) $$e_{r}={\left[{\left(1+\frac{du}{dr}\right)}^{2}+{\left(\frac{dw}{dr}\right)}^{2}\right]}^{1/2}-1$$

and

(6) $$e_{t}=\frac{u}{r},$$

where *e_r_*, *e_t_* and *u* denote the radial and circumferential strains and radial displacement, respectively. Moreover, the relationships between stress and strain are still assumed to be linearly elastic and given by the generalized Hooke’s law

(7) $$\sigma_{r}=\frac{E}{1-\nu^{2}}(e_{r}+\nu e_{t})$$

and

(8) $$\sigma_{t}=\frac{E}{1-\nu^{2}}(e_{t}+\nu e_{r}).$$

Substituting Equations (5) and (6) into Equations (7) and (8) yields

(9) $$\sigma_{r}=\frac{E}{1-\nu^{2}}\{{\left[{\left(1+\frac{du}{dr}\right)}^{2}+{\left(\frac{dw}{dr}\right)}^{2}\right]}^{1/2}-1+\nu \frac{u}{r}\}$$

and

(10) $$\sigma_{t}=\frac{E}{1-\nu^{2}}\{\frac{u}{r}+\nu {\left[{\left(1+\frac{du}{dr}\right)}^{2}+{\left(\frac{dw}{dr}\right)}^{2}\right]}^{1/2}-\nu \}.$$

By means of Equations (4), (9) and (10), one has

(11) $$\frac{u}{r}=\frac{1}{E}(\sigma_{t}-\nu \sigma_{r}).$$

After substituting the *u* in Equation (11) into Equation (9), the consistency equation may be written as

(12) $${\left(\frac{1}{E}\sigma_{r}+1-\frac{\nu }{E}\sigma_{t}\right)}^{2}-{\left\{1+\frac{d}{dr}[\frac{1}{E}r(\sigma_{t}-\nu \sigma_{r})]\right\}}^{2}-{\left(\frac{dw}{dr}\right)}^{2}=0.$$

Therefore, the radial and circumferential stresses σ*_r_* and σ*_t_* and the deflection *w*(*r*) within *b* ≤ *r* ≤ *a* can be determined by simultaneously solving Equations (3), (4) and (12). The conditions for determining the special solutions of σ*_r_*, σ*_t_* and *w*(*r*) are the boundary conditions at *r* = *a*, as well as the continuity conditions at *r* = *b*, which can be determined only by solving the plane problem of axisymmetric stretching of the circular membrane in the plate/membrane contact area with radius 0 ≤ *r* ≤ *b*, which is detailed as follows.

Obviously, in the plate/membrane contact area with radius 0 ≤ *r* ≤ *b*, the membrane is flat, then the first derivative of the deflection *w*(*r*) is always zero, i.e., d*w*/d*r ≡* 0 for 0 ≤ *r* ≤ *b*. Therefore, it can be obtained from Equations (5) and (6) that

(13) $$e_{r}=\frac{du}{dr}$$

and

(14) $$e_{t}=\frac{u}{r}.$$

Substituting Equations (13) and (14) into Equations (7) and (8) yields

(15) $$\sigma_{r}=\frac{E}{1-\nu^{2}}(\frac{du}{dr}+\nu \frac{u}{r})$$

and

(16) $$\sigma_{t}=\frac{E}{1-\nu^{2}}(\frac{u}{r}+\nu \frac{du}{dr}).$$

Substituting Equations (15) and (16) into Equation (4), one has

(17) $$r\frac{d^{2}u}{dr^{2}}+\frac{du}{dr}-\frac{u}{r}=0.$$

Since Equation (17) satisfies the form of the Euler equation, the general solution of Equation (17) can be written as

(18) $$u(r)=K_{1}r+\frac{K_{2}}{r},$$

where *K*_1_ and *K*_2_ are two unknown constants. It is not difficult to understand that since the radial displacement *u* is finite at *r* = 0, the unknown constant *K*_2_ has to be equal to zero. So, if the radial displacement *u*(*r*) at *r = b* is denoted by *u*(*b*), then *K*_1_ = *u*(*b*)/*b*. Therefore, the radial displacement may be written as

(19) $$u(r)=\frac{u(b)}{b}r.$$

Substituting Equation (19) into Equations (13)–(16) yields

(20) $$e_{r}=e_{t}=\frac{u(b)}{b}$$

and

(21) $$\sigma_{r}=\sigma_{t}=\frac{E}{1-\nu }\frac{u(b)}{b}.$$

Equations (20) and (21) suggest that the strain and stress are uniformly distributed in the plate/membrane contact area with radius 0 ≤ *r* ≤ *b*.

Therefore, the boundary conditions at *r* = *a* are

(22) w=0 at r=a

and

(23) $$e_{t}=\frac{1}{E}(\sigma_{t}-\nu \sigma_{r})=0\;\mathrm{at}\;r=a.$$

The continuity conditions at *r* = *b* are

(24) w=g at r=b,

(25) $${\left(\frac{u}{r}\right)}_{A}={\left(\frac{u}{r}\right)}_{B}=\frac{u(b)}{b}\;\mathrm{at}\;r=b$$

and

(26) $${\left(\sigma_{r}\right)}_{A}={\left(\sigma_{r}\right)}_{B}=\frac{E}{1-\nu }\frac{u(b)}{b}\;\mathrm{at}\;r=b,$$

where ( )*_A_* and ( )*_B_* represent the values of various variables on two sides of the interconnecting circle of *r* = *b* and the subscript *A* refers to the side of plate/membrane non-contact area of *b* ≤ *r* ≤ *a* while the subscript *B* refers to the side of plate/membrane contact area of 0 ≤ *r* ≤ *b*. 

Let us introduce the following dimensionless variables

(27) $$Q=\frac{qa}{Eh},\;W=\frac{w}{a},\;S_{r}=\frac{\sigma_{r}}{E},\;S_{t}=\frac{\sigma_{t}}{E},\;x=\frac{r}{a},\;\alpha =\frac{b}{a},$$

and transform Equations (3), (4), (12) and (22)–(26) into

(28) $$[4x^{2}{S_{r}}^{2}-Q^{2}{\left(x^{2}-\alpha^{2}\right)}^{2}]{\left(\frac{dW}{dx}\right)}^{2}-Q^{2}{\left(x^{2}-\alpha^{2}\right)}^{2}=0,$$

(29) $$(\frac{dS_{r}}{dx}x+S_{r})[1+{\left(\frac{dW}{dx}\right)}^{2}]-\frac{dW}{dx}\frac{d^{2}W}{dx^{2}}S_{r}x-S_{t}{\left[1+{\left(\frac{dW}{dx}\right)}^{2}\right]}^{2}=0,$$

(30) $${\left(S_{r}+1-\nu S_{t}\right)}^{2}-{\left(1+S_{t}+x\frac{dS_{t}}{dx}-\nu S_{r}-\nu x\frac{dS_{r}}{dx}\right)}^{2}-{\left(\frac{dW}{dx}\right)}^{2}=0,$$

(31) W=0 at x=1,

(32) $$S_{t}-\nu S_{r}=0\;\mathrm{at}\;x=1,$$

(33) $$W=\frac{g}{a}\;\mathrm{at}\;x=\alpha ,$$

(34) $${\left(S_{t}-\nu S_{r}\right)}_{A}={\left(S_{t}-\nu S_{r}\right)}_{B}=\frac{u(b)}{b}\;\mathrm{at}\;x=\alpha$$

and

(35) $${\left(S_{r}\right)}_{A}={\left(S_{r}\right)}_{B}=\frac{1}{1-\nu }\frac{u(b)}{b}\;\mathrm{at}\;x=\alpha .$$

Since the stress and deflection are all finite in the plate/membrane non-contact annular area of *b* ≤ *r* ≤ *a* (i.e., *α* ≤ *x* ≤ 1), *S_r_* and *W* can be expanded as the power series of *x* − *β*,

(36) $$S_{r}=\sum_{i=0}^{\infty }b_{i}{\left(x-\beta \right)}^{i},$$

(37) $$S_{t}=\sum_{i=0}^{\infty }c_{i}{\left(x-\beta \right)}^{i}$$

and

(38) $$W=\sum_{i=0}^{\infty }d_{i}{\left(x-\beta \right)}^{i}$$

where *β* = (1 + *α*)/2 and *α* = *b*/*a*. After substituting Equations (36)–(38) into Equations (28)–(30), all the coefficients *b_i_*, *c_i_* and *d_i_* (*i* = 1, 2, 3, …) can be expressed as the polynomials of *b*_0_, *c*_0_ and *β* (i.e., (1 + *α*)/2), which are listed in Appendix A. The coefficients *b*_0_, *c*_0_ and *β* are called undetermined constants, where since *β* = (1 + *α*)/2 and *α* = *b*/*a*, the undetermined constant *β* actually represents the unknown plate/membrane contact radius *b* that needs to be determined. The remaining coefficient *d*_0_ is the other undetermined constant that depends on *b*_0_, *c*_0_ and *β*. All the undetermined constants *b*_0_, *c*_0_, *β* and *d*_0_ can be determined using the boundary conditions and continuity conditions as follows. 

From Equation (38), Equations (31) and (33) give

(39) $$\sum_{i=0}^{\infty }d_{i}{\left(1-\beta \right)}^{i}=0$$

and

(40) $$\sum_{i=0}^{\infty }d_{i}{\left(\alpha -\beta \right)}^{i}=\frac{g}{a}.$$

Eliminating *d*_0_ by Equation (40) minus Equation (39) yields

(41) $$\sum_{i=1}^{\infty }d_{i}[{\left(\alpha -\beta \right)}^{i}-{\left(1-\beta \right)}^{i}]=\frac{g}{a}.$$

From Equations (36) and (37), Equations (32), (34) and (35) give

(42) $$\sum_{i=0}^{n}c_{i}{\left(1-\beta \right)}^{i}-\nu \sum_{i=0}^{n}b_{i}{\left(1-\beta \right)}^{i}=0,$$

(43) $$\sum_{i=0}^{n}c_{i}{\left(\alpha -\beta \right)}^{i}-\nu \sum_{i=0}^{n}b_{i}{\left(\alpha -\beta \right)}^{i}=\frac{u(b)}{b}$$

and

(44) $$\sum_{i=0}^{\infty }b_{i}{\left(\alpha -\beta \right)}^{i}=\frac{1}{1-\nu }\frac{u(b)}{b}.$$

Eliminating the *u*(*b*)/*b* from Equations (43) and (44), one has

(45) $$\sum_{i=0}^{n}c_{i}{\left(\alpha -\beta \right)}^{i}-\sum_{i=0}^{n}b_{i}{\left(\alpha -\beta \right)}^{i}=0.$$

So, for a given problem where *a*, *h*, *E*, *υ*, *g*, and *q* are known in advance, the undetermined constants *c*_0_, *c*_1_, and *β* can be determined by simultaneously solving Equations (41), (42) and (45). Furthermore, with the known *c*_0_, *c*_1_ and *β*, the undetermined constant *d*_0_ can be determined by Equations (39) or (40). Thus, the plate/membrane contact problem dealt with here is solved analytically.

In addition, after considering Equation (27) and *β* = (1 + *α*)/2 and *α* = *b*/*a*, from Equations (36)–(38), the dimensional stress and deflection may finally be written as

(46) $$\sigma_{r}=E\sum_{i=0}^{\infty }b_{i}{\left(\frac{r}{a}-\frac{a+b}{2a}\right)}^{i},$$

(47) $$\sigma_{t}=E\sum_{i=0}^{\infty }c_{i}{\left(\frac{r}{a}-\frac{a+b}{2a}\right)}^{i}$$

and

(48) $$w=a\sum_{i=0}^{\infty }d_{i}{\left(\frac{r}{a}-\frac{a+b}{2a}\right)}^{i}.$$

The maximum stress in membrane should be at *r* = *b*, then

(49) $$\sigma_{m}=E\sum_{i=0}^{\infty }b_{i}{\left(\frac{b-a}{2a}\right)}^{i}$$

or

(50) $$\sigma_{m}=E\sum_{i=0}^{\infty }c_{i}{\left(\frac{b-a}{2a}\right)}^{i}.$$

## 3. Pressure–Capacitance Relationship Derivation of the Sensing Element of the Sensor

The circular capacitive pressure sensor in Figure 1 uses the variable capacitor between the movable and fixed electrode plates as a sensing element, and when the pressure *q* exceeds the touch point pressure $q_{\mathrm{TPP}}$ , a circular plate/membrane contact area with radius *b* is formed between the deflected circular conductive membrane and the insulator layer, as shown in Figure 1d, where the contact radius *b* will increase with the further increase in the pressure *q*. The circular capacitive pressure sensor in Figure 1d is said to operate in touch mode and is referred to as the circular touch mode capacitive pressure sensor for short.

**Figure 3:**
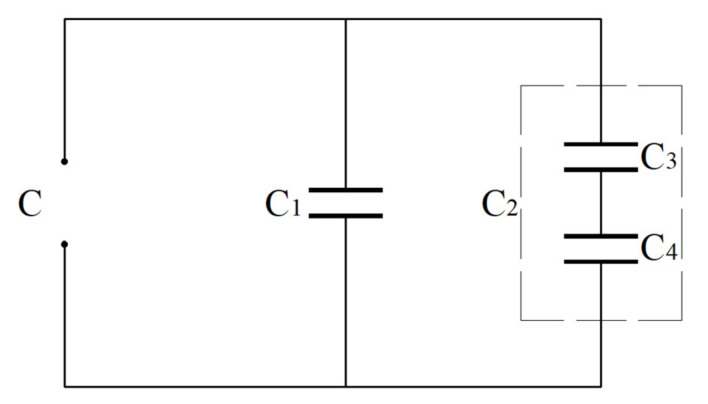
Sketch of series and parallel relationships of the capacitors in the circular capacitive pressure sensor of touch mode of operation in Figure 1d.

In order to facilitate the calculation of the total capacitance of the circular touch mode capacitive pressure sensor in Figure 1d, the total capacitor (denoted by C) between the movable and fixed electrode plates can be regarded as one consisting of two capacitors in parallel (denoted by C_1_ and C_2_). C_1_ refers to the parallel plate capacitor in the plate/membrane contact area of *0* ≤ *r* ≤ *b* (the parallel gap between its two electrode plates is equal to the thickness *t* of the insulator layer, see Figure 1d), and is a variable capacitor due to the gradually increasing *b*. C_2_ refers to the non-parallel plate capacitor in the plate/membrane non-contact area of *b* ≤ *r* ≤ *a*, and can be regarded as one consisting of two capacitors in series (denoted by C_3_ and C_4_). C_3_ refers to the parallel plate capacitor in the plate/membrane non-contact area of *b* ≤ *r* ≤ *a* (the parallel gap between its two electrode plates is equal to the thickness *t* of the insulator layer, see Figure 1d) and is a variable capacitor due to the gradually increasing *b* with the gradually increasing pressure *q*. C_4_ refers to the non-parallel plate capacitor in the plate/membrane non-contact area of *b* ≤ *r* ≤ *a* (the non-parallel gap between its two electrode plates is *g–w*(*r*), see Figure 1d) and is also a variable capacitor due to the gradually increasing *b* as well as the non-parallel gap *g–w*(*r*) varying with the pressure *q*. In addition, it can be seen from Figure 1d that the two electrode plates of the capacitors C_1_ and C_3_ are separated by the insulator layer and the two electrode plates of the capacitors C_4_ are separated by the air. Let us denote the vacuum permittivity by *ε*_0_ (about 8.854 × 10^−3^ pF/mm), the relative permittivity of the insulator layer by *ε*_r1_ and the relative permittivity of the air by *ε*_r2_ (about 1.00053). The series and parallel relationships of the capacitors C_1_, C_2_, C_3_ and C_4_ are shown in Figure 3.

As can be seen from Figure 1d, the parallel plate capacitor C_1_ locates in the plate/membrane contact area of *0* ≤ *r* ≤ *b*, so its capacitance may be written as

(51) $$C_{1}=\frac{\varepsilon_{0}\varepsilon_{r1}\pi b^{2}}{t},$$

and the parallel plate capacitor C_3_ locates in the plate/membrane non-contact area of *b* ≤ *r* ≤ *a*, so its capacitance may be written as

(52) $$C_{3}=\frac{\varepsilon_{0}\varepsilon_{r1}\pi (a^{2}-b^{2})}{t}.$$

**Figure 4:**
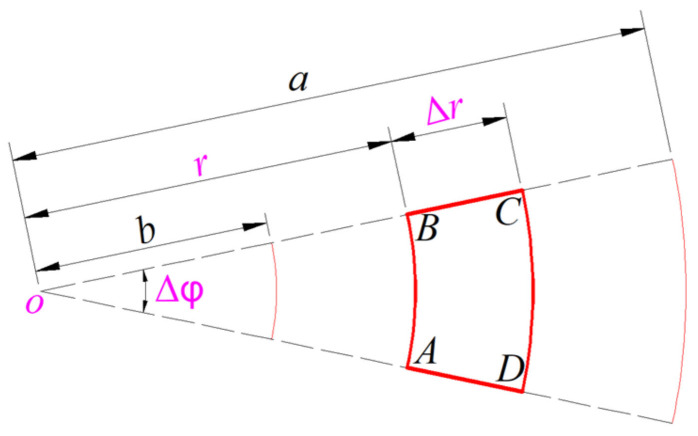
A micro area element ABCD taken from the deflected membrane in the plate/membrane non-contact region of *b* ≤ *r* ≤ *a* in Figure 1d.

It can also be seen from Figure 1d that the non-parallel plate capacitor C_4_ also locates in the plate/membrane non-contact area of *b* ≤ *r* ≤ *a*, so its capacitance expression can be derived as follows. A micro area element with radial increment ∆*r* and circumferential increment ∆*φ*, ABCD, is taken from the deflected membrane in the plate/membrane non-contact region of *b* ≤ *r* ≤ *a* in Figure 1d, as shown in Figure 4. Therefore, the non-parallel plate capacitor C_4_ can be regarded as one consisting of infinitely many tiny capacitors in parallel, where each tiny capacitor occupies a micro area element ABCD and is approximated by a tiny parallel plate capacitor.

The area of the micro area element ABCD is

(53) $$\Delta S_{ABCD}=\frac{{\left(r+\Delta r\right)}^{2}\Delta \varphi }{2}-\frac{r^{2}\Delta \varphi }{2}=r\Delta r\Delta \varphi +\frac{1}{2}{\left(\Delta r\right)}^{2}\Delta \varphi .$$

In Equation (53), the high order infinitesimal term (Δ*r*)^2^∆*φ* can be ignored, and the area Δ*S*_ABCD_ can thus be approximated by *r*Δ*r*∆*φ*. The air gap of the tiny parallel plate capacitor corresponding to the micro area element ABCD can be approximated by *g*–*w*(*r*) (see Figure 1d and Figure 4). Therefore, after considering Equation (48), the capacitance of this tiny parallel plate capacitor may be written as

(54) $$\Delta C=\varepsilon_{0}\varepsilon_{r2}\frac{r\Delta r\Delta \varphi }{g-w(r)}=\varepsilon_{0}\varepsilon_{r2}\frac{r}{g-a\sum_{i=0}^{\infty }d_{i}{\left(\frac{r}{a}-\frac{b}{2a}-\frac{1}{2}\right)}^{i}}\Delta r\Delta \varphi .$$

Then, the capacitance of infinitely many tiny parallel plate capacitors in parallel, that is, the capacitance of the non-parallel plate capacitor C_4_, can be obtained by the integration of Equation (54) over the plate /membrane non-contact region of *b* ≤ *r* ≤ *a* and 0 ≤ *φ* ≤ 2π

(55) $$C_{4}=\int_{b}^{a}\int_{0}^{2\pi }\varepsilon_{0}\varepsilon_{r2}\frac{r}{g-a\sum_{i=0}^{\infty }d_{i}{\left(\frac{r}{a}-\frac{b}{2a}-\frac{1}{2}\right)}^{i}}d\varphi dr=2\pi \varepsilon_{0}\varepsilon_{r2}\int_{b}^{a}\frac{r}{g-a\sum_{i=0}^{\infty }d_{i}{\left(\frac{r}{a}-\frac{b}{2a}-\frac{1}{2}\right)}^{i}}dr.$$

Then, from Equations (52) and (55), the capacitance of the non-parallel plate capacitor C_2_ (formed by C_3_ and C_4_ in series) may be written as

(56) $$C_{2}=\frac{C_{3}C_{4}}{C_{3}+C_{4}}=\frac{\frac{\varepsilon_{0}\varepsilon_{r1}\pi {\left(a-b\right)}^{2}}{t}2\pi \varepsilon_{0}\varepsilon_{r2}\int_{0}^{a}\frac{r}{g-a\sum_{i=0}^{\infty }d_{i}{\left(\frac{r}{a}-\frac{b}{2a}-\frac{1}{2}\right)}^{i}}dr}{\frac{\varepsilon_{0}\varepsilon_{r1}\pi {\left(a-b\right)}^{2}}{t}+2\pi \varepsilon_{0}\varepsilon_{r2}\int_{0}^{a}\frac{r}{g-a\sum_{i=0}^{\infty }d_{i}{\left(\frac{r}{a}-\frac{b}{2a}-\frac{1}{2}\right)}^{i}}dr}.$$

Therefore, from Equations (51) and (56), the capacitance of the total capacitor C (formed by C_1_ and C_2_ in parallel) between the movable and fixed electrode plates may finally be written as

(57) $$C=C_{1}+C_{2}=\frac{\varepsilon_{0}\varepsilon_{r1}\pi b^{2}}{t}+\frac{\frac{\varepsilon_{0}\varepsilon_{r1}\pi {\left(a-b\right)}^{2}}{t}2\pi \varepsilon_{0}\varepsilon_{r2}\int_{0}^{a}\frac{r}{g-a\sum_{i=0}^{\infty }d_{i}{\left(\frac{r}{a}-\frac{b}{2a}-\frac{1}{2}\right)}^{i}}dr}{\frac{\varepsilon_{0}\varepsilon_{r1}\pi {\left(a-b\right)}^{2}}{t}+2\pi \varepsilon_{0}\varepsilon_{r2}\int_{0}^{a}\frac{r}{g-a\sum_{i=0}^{\infty }d_{i}{\left(\frac{r}{a}-\frac{b}{2a}-\frac{1}{2}\right)}^{i}}dr}.$$

It can be seen from Equation (57) that the total capacitances *C* can be determined as long as the deflection expression *w*(*r*) is available, i.e., as long as the power series coefficients *d_i_* (*i* = 0, 1, 2, 3, *…*) in Equation (48) are known. However, Equation (57) gives only the analytical relationship of the pressure *q* as an independent variable (or input variable) and the capacitance *C* as a dependent variable (or output variable), that is, the pressure–capacitance analytical relationship, where the pressure *q* is included in the expressions of the power series coefficients *d_i_* (*i* = 0, 1, 2, 3, *…*) (see Appendix A). But the sensor mechanism of such capacitive pressure sensors is to detect the applied pressure *q* by measuring the capacitance *C* under the applied pressure *q*. So, it is necessary to give the analytical relationship of the capacitance *C* as an independent variable (or input variable) and the pressure *q* as a dependent variable (or output variable), i.e., the capacitance–pressure analytical relationship. Obviously, due to the strong nonlinearity of Equation (57), the capacitance–pressure analytical relationship cannot be derived directly using Equation (57). Therefore, the capacitance–pressure analytical relationship has to resort to numerical calculations, where the analytical solution obtained in Section 2 as well as Equation (57) are first used to calculate the total capacitances *C* and their corresponding pressure *q* for a specific problem, and then, based on the obtained large number of capacitances and pressure numerical calculation values, the capacitance–pressure analytical relationship can be obtained using least-squares data fitting, which will be shown in Section 4.1.

## 4. Results and Discussion

In this section, an example is first given to illustrate how to use the analytical solutions of the large deflection problem and plate/membrane contact problem as well as the pressure–capacitance analytical relationship to design and numerically calibrate a specific circular touch mode capacitive pressure sensor, which is shown in Section 4.1. Then, the effect of changing design parameters on the capacitance–pressure analytical relationships is comprehensively investigated, including all design parameters (the initially parallel gap *g*, membrane thickness *h*, Young’s modulus of elasticity *E*, Poisson’s ratio *v*, insulator layer thickness *t*, circular membrane radius *a* and the number of parallel capacitors *n*), which is shown in Section 4.2, Section 4.3, Section 4.4, Section 4.5, Section 4.6, Section 4.7 and Section 4.8

### 4.1. Design and Numerical Calibration Based on Analytical Solutions

How to use the analytical solution of the plate/membrane contact problem (derived in Section 2) and the analytical solution of the large deflection problem (derived in [25]), as well as the pressure–capacitance analytical relationship (i.e., Equation (57) derived in Section 3) to design and numerically calibrate a circular touch mode capacitive pressure sensor with a specified pressure detecting range is detailed as follows.

The initially parallel gap between the initially flat circular conductive membrane and the insulator layer (see Figure 1a), *g*, needs to be first determined. Obviously, the touch point pressure $q_{\mathrm{TPP}}$ should be equal to the lower limit of the pressure detecting range required or desired by the design of the circular touch mode capacitive pressure sensor. Therefore, the initially parallel gap *g* should be equal to the maximum deflection *w*_m_ of the circular conductive membrane under the required or desired lower limit pressure, i.e., under the touch point pressure $q_{\mathrm{TPP}}$ . To this end, the analytical solution for the large deflection problem of a circular membrane under transverse loading, which is presented in [25], is used to determine the maximum deflection *w*_m_ of the circular conductive membrane under the touch point pressure $q_{\mathrm{TPP}}$ , where the undetermined constants *b*_0_, *c*_0_ and *d*_0_ should be first determined, and then the maximum deflection *w*_m_ and maximum stress *σ*_m_ should be calculated. If the calculated maximum stress *σ*_m_ is relatively small (about 0.2 times the yield strength *σ*_y_ of the used membrane materials), then the calculated maximum deflection *w*_m_ can be used as the initially parallel gap *g*; otherwise, it is necessary to change the design parameters of the circular conductive membrane (such as radius *a*, thickness *h*, Poisson’s ratio *v* and Young’s modulus of elasticity *E*) and repeat the above calculation until the requirement for *σ*_m_ ≤ 0.2*σ*_y_ is met.

After the initially parallel gap *g* is determined, the maximum stress *σ*_m_ of the circular conductive membrane under the upper limit pressure of the required or desired detecting range needs to be calculated using the analytical solution of the plate/membrane contact problem derived in Section 2. If the calculated maximum stress *σ*_m_ does not exceed 0.7 times the yield strength *σ*_y_, then the next step can perform the numerical calibration, otherwise, it is necessary to change the design parameters of the circular conductive membrane (such as radius *a*, thickness *h*, Poisson’s ratio *v* and Young’s modulus of elasticity *E*) and repeat the above calculation until the requirement for *σ*_m_ ≤ 0.7*σ*_y_ is met.

The numerical calibration can be performed using the analytical solution of the plate/membrane contact problem derived in Section 2 and the pressure–capacitance analytical relationship (i.e., Equation (57) derived in Section 3). The numerical calculations can start from the required or desired lower limit pressure (i.e., the touch point pressure $q_{\mathrm{TPP}}$ ) plus a pressure increment as small as possible. Equations (41), (42) and (45) are first used to determine the undetermined constants *c*_0_, *c*_1_, and *β* (*β* = (1 + *α*)/2 and *α* = *b*/*a*), and with the known *c*_0_, *c*_1_, and *β*, the other undetermined constant *d*_0_ can be determined by Equations (39) or (40). Further, with the known *c*_0_, *c*_1_, *β* and *d*_0_, all the power series coefficient *c_i_* and *d_i_* can be determined. The maximum stress σ_m_ can be determined by Equation (49) or by Equation (50) (whichever is the maximum), and the total capacitances *C* under this pressure can be determined by Equation (57). Then, a pressure increment was added to continue the numerical calculation until the repeatedly increased pressure is equal to the upper limit pressure of the required or desired detecting range. And finally, all the numerical calculation values of the total capacitances *C* and their corresponding pressures *q* are collected, and used to establish the capacitance–pressure analytical relationship using least-squares data fitting.

Suppose that the required or desired pressure detecting range of a circular touch mode capacitive pressure sensor to be designed is *q* = 2.718–45 KPa, and a circular conductive membrane with radius *a* = 100 mm, thickness *h* = 1 mm, Young’s modulus of elasticity *E* = 7.84 MPa, Poisson’s ratio *v* = 0.47, and yield strength *σ*_y_ = 2.4 MPa is assumed to be used. The insulator layer is assumed to take 0.1 mm thickness of polystyrene, then *t* = 0.1 mm and *ε*_r1_ = 2.7. In addition, the vacuum permittivity is *ε*_0_ = 8.854 × 10^−3^ pF/mm, and the air relative permittivity is *ε*_r2_ = 1.00053. The design and numerical calibration of this circular touch mode capacitive pressure sensor are as follows.

The analytical solution for the large deflection problem of a circular membrane under transverse loading, which is presented in [25], is first used to calculate the maximum deflection *w*_m_ of the circular conductive membrane under *q* = 2.718 KPa (the lower limit of the required or desired pressure detecting range, i.e., $q_{\mathrm{TPP}}=2.718\mathrm{KPa}$ ). The calculated maximum deflection *w*_m_ is about 19.998 mm and the calculated maximum stress *σ*_m_ is about 0.332 MPa, where the undetermined constants are *b*_0_ = 0.04603, *c*_0_ = 0.04113 and *d*_0_ = 0.15332. Therefore, the initially parallel gap *g* can take 20 mm, and the calculated maximum stress *σ*_m_ (0.332 MPa) is less than 0.2*σ*_y_ (0.48 MPa).

The analytical solution for the plate/membrane contact problem derived in Section 2 is used to calculate the maximum stress *σ*_m_ of the circular conductive membrane under *q*= 45 KPa (the upper limit pressure of the required or desired detecting range). The calculated maximum stress *σ*_m_ is about 1.679 MPa and is less than 0.7*σ*_y_ (1.68 MPa). Therefore, the numerical calibration can be further performed using the analytical solution for the plate/membrane contact problem derived in Section 2 and the pressure–capacitance analytical relationship (i.e., Equation (57) derived in Section 3).

The numerical calculations of the total capacitances *C* under different pressures *q* start from the pressure *q* = 2.718 KPa, and then the pressure *q* is gradually increased, as shown in Table 1, where the undetermined constants *c*_0_, *c*_1_ and *β* (*β* = (1 + *α*)/2 and *α* = *b*/*a*) are determined by simultaneously solving Equations (41), (42) and (45), the undetermined constant *d*_0_ is determined by Equations (39) or (40) with the known *c*_0_, *c*_1_ and *β*, the maximum stress σ_m_ is determined using Equation (49) (the radial maximum stress obtained by Equation (49) is greater than the circumferential maximum stress obtained by Equation (50)), the total capacitances *C* of the total capacitor C between the movable and fixed electrode plates are determined using Equation (57), the capacitance *C*_1_ of the parallel plate capacitor C_1_ in the plate/membrane contact area of *0* ≤ *r* ≤ *b* is determined using Equation (51) and the capacitance *C*_2_ of the non-parallel plate capacitor C_2_ in the plate/membrane non-contact area of *b* ≤ *r* ≤ *a* is determined using Equation (56).

**Figure 5:**
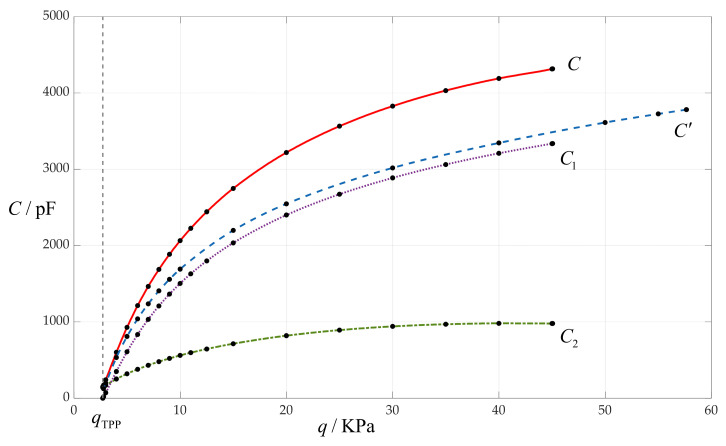
The variation in the total capacitances *C* with the applied pressure *q* when *a* = 100 mm, *h* = 1 mm, *t* = 0.1 mm, *E* = 7.84 MPa, *ν* = 0.47 and *g* = 20 mm.

In Table 1, the capacitance *C*_1_ of the parallel plate capacitor C_1_ in the plate/membrane contact area of *0* ≤ *r* ≤ *b* and the capacitance *C*_2_ of the non-parallel plate capacitor C_2_ in the plate/membrane non-contact area of *b* ≤ *r* ≤ *a* are calculated specifically for discussion of the following issue. The total capacitance *C* of a touch mode capacitive pressure sensor is often assumed to be mainly equal to the capacitance *C*_1_, that is, the capacitance *C*_2_ can be neglected [15,20,21,44,47,48]. However, it can be seen from Figure 5 that adopting this assumption will cause the designed touch mode capacitive pressure sensor to lose too much accuracy. So, it is suggested that full attention should be paid to this, especially for detecting a lower pressure range, for example, the low pressure range 3–10 MPa in Figure 5.

In Figure 5, the dashed line, *C*′, represents the total capacitances calculated using the previously derived analytical solution in [11,29]. It can be seen from Figure 5 that the dashed line *C*′ gradually deviates from the solid line *C* (the total capacitances calculated using the newly derived analytical solution in this paper), and in particular, the degree of deviation becomes larger and larger as the pressure increases. This suggests that in comparison with the previously derived analytical solution in [11,29], the newly derived analytical solution in this paper has indeed been greatly improved and can provide a better support for designing circular touch mode capacitive pressure sensors.

**Figure 6:**
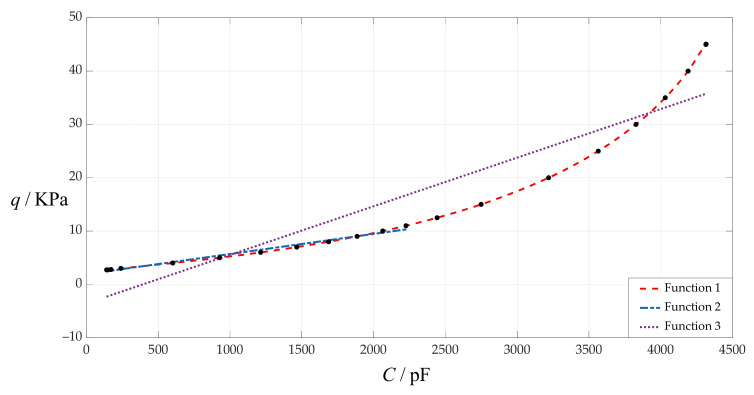
The variation in the applied pressure *q* with the total capacitances *C* when *a* = 100 mm, *h* = 1 mm, *t* = 0.1 mm, *E* = 7.84 MPa, *ν* = 0.47 and *g* = 20 mm.

As mentioned above, the sensor mechanism of such capacitive pressure sensors is to detect the applied pressure *q* by measuring the capacitance *C* under the applied pressure *q*. Therefore, it is necessary to give the analytical relationship of the capacitance *C* as an independent variable or an input variable and the pressure *q* as a dependent variable or an output variable, i.e., the capacitance–pressure analytical relationship. Based on the numerical calculation values of capacitance and pressure in Table 1, the capacitance–pressure analytical relationships are obtained using least-squares data fitting, as shown in Figure 6 and Table 2, where “Function 1” refers to the fitting result using a sixth-power function, and “Function 2” and “Function 3” refer to the fitting results using a straight line (see Table 2).

It can be seen from Figure 6 and Table 2 that the circular touch mode capacitive pressure sensor to be designed, whose pressure detecting range is *q* = 2.718–45 KPa, can be achieved using “Function 1” or “Function 3” in Figure 6 and Table 2. However, as shown in Table 2, “Function 1” is a sixth-power function but with high fitting accuracy, while “Function 3” is a straight line function but with unacceptable fitting accuracy. Therefore, although “Function 3” can be used to develop a linear sensor and “Function 1” can only be used to develop a nonlinear sensor, the fitting accuracy of “Function 3” is completely unacceptable, so “Function 1” has to be used to develop a nonlinear sensor. Of course, “Function 2” is a straight line function with acceptable fitting accuracy, but it can only be used to develop a linear sensor with a pressure detecting range of 2.718~11 KPa (see Table 2).

Therefore, if it is necessary to develop a linear sensor with a pressure detecting range of 2.718~45 KPa, the only way is to continuously change the design parameters, such as the initially parallel gap *g*, membrane thickness *h*, Young’s modulus of elasticity *E*, Poisson’s ratio *v*, insulator layer thickness *t* and the circular membrane radius *a*, until a linear relationship (with a pressure detecting range of 2.718~45 KPa and with an acceptable fitting accuracy) is fitted. In fact, the analytical relationship of capacitance *C* as an independent variable or an input variable and pressure *q* as a dependent variable or an output variable (hereinafter referred to as the *C–q* relationship) to be fitted usually has requirements of both the output pressure range and the input capacitance range. The requirement of the output pressure range is to meet the required or desired pressure detecting range, and the requirement of the input capacitance range is to meet the requirement of sensitivity when the sensor is designed.

Therefore, it is very important for sensor design to know the effect of changing the design parameters on the *C–q* relationships. In other words, one needs to know which design parameters (*g*, *h*, *E*, *v*, *t* or *a*) can be changed (increased or decreased) to increase the output pressure range to meet the required or desired pressure detecting range, and which design parameters can be changed to increase the input capacitance range to meet the sensitivity requirement. So, in this sense, it is very important to correctly understand how changing design parameters will affect the *C–q* relationships (which will be addressed in Section 4.2, Section 4.3, Section 4.4, Section 4.5, Section 4.6, Section 4.7 and Section 4.8); after all, the fabrication of sensors can only be considered after all the design parameters (*g*, *h*, *E*, *v*, *t* and *a*) have been determined.

### 4.2. The Effect of Changing Initially Parallel Gap g on C–q Relationships

**Figure 7:**
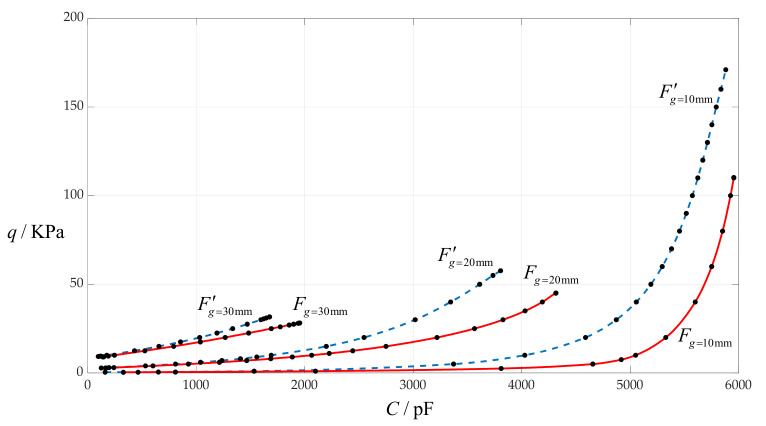
The effect of changing the initially parallel gap *g* on the *C*–*q* relationships when *a* = 100 mm, *h* = 1 mm, *t* = 0.1 mm, *E* = 7.84 MPa, *ν* = 0.47 and *g* takes 10 mm, 20 mm and 30 mm, respectively.

In this section, the initially parallel gap *g* takes 10 mm, 20 mm and 30 mm, respectively, while the other design parameters remain unchanged, that is, the circular membrane radius *a* = 100 mm, membrane thickness *h* = 1 mm, insulator layer thickness *t* = 0.1 mm, Young’s modulus of elasticity *E* = 7.84 MPa and Poisson’s ratio *v* = 0.47. In addition, the vacuum permittivity is *ε*_0_ = 8.854 × 10^−3^ pF/mm, the relative permittivity of the insulator layer (polystyrene) is *ε*_r1_ = 2.7 and the air relative permittivity is *ε*_r2_ = 1.00053. The calculation results of the total capacitances *C* under different pressures *q* are listed in Table 3 when *g* = 10 mm, in Table 1 when *g* = 20 mm and in Table 4 when *g* = 30 mm. The effect of changing the initially parallel gap *g* on the *C*–*q* relationships is shown in Figure 7, where the solid lines *F_g_*_= 10mm_, *F_g_*_= 20mm_ and *F_g_*_= 30mm_ refer to the calculation results when *g* = 10 mm, 20 mm and 30 mm, which are obtained using the newly derived analytical solution in this paper, and the dotted lines *F*′*_g_*_= 10mm_, *F*′*_g_*_= 20mm_ and *F*′*_g_*_= 30mm_ refer to the calculation results when *g* = 10 mm, 20 mm and 30 mm, which are obtained using the previously derived analytical solutions in [11,29].

From Figure 7, it can be seen that decreasing the initially parallel gap *g* can increase both the output pressure range and the input capacitance range, but it can also increase the nonlinear strength of the *C*–*q* relationships. In addition, the obvious differences between the solid and dotted lines once again suggest that the newly derived analytical solution in this paper has been greatly improved, and can provide a better support for designing circular touch mode capacitive pressure sensors, in comparison with the previously derived analytical solutions in [11,29].

### 4.3. The Effect of Changing Circular Membrane Thickness h on C–q Relationships

**Figure 8:**
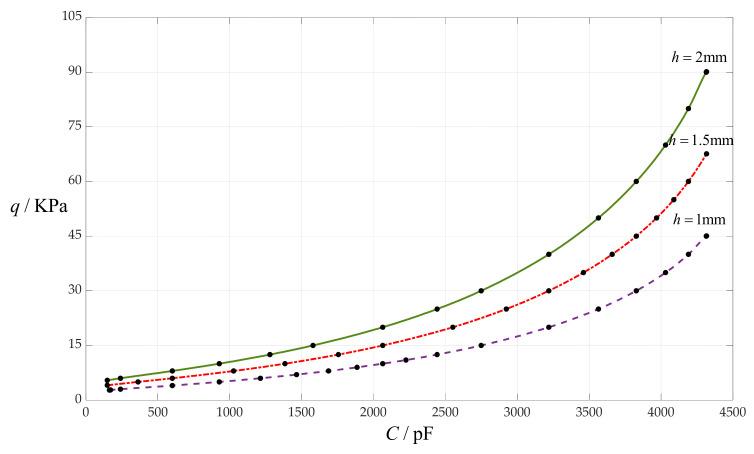
The effect of changing the circular membrane thickness *h* on the *C*–*q* relationships when *a* = 100 mm, *g* = 20 mm, *t* = 0.1 mm, *E* = 7.84 MPa, *ν* = 0.47 and *h* takes 1 mm, 1.5 mm and 2 mm, respectively.

In this section, the circular membrane thickness *h* takes 1 mm, 1.5 mm and 2 mm, respectively, while the other design parameters remain unchanged, that is, the circular membrane radius *a* = 100 mm, the initially parallel gap *g* = 20 mm, insulator layer thickness *t* = 0.1 mm, Young’s modulus of elasticity *E* = 7.84 MPa and Poisson’s ratio *v* = 0.47. In addition, the vacuum permittivity is still *ε*_0_ = 8.854 × 10^−3^ pF/mm, the relative permittivity of the insulator layer (polystyrene) is *ε*_r1_ = 2.7, and the air relative permittivity is *ε*_r2_ = 1.00053. The calculation results of the total capacitances *C* under different pressures *q* are listed in Table 1 when *h* = 1 mm, in Table 5 when *h* = 1.5 mm and in Table 6 when *h* = 2 mm. The effect of changing the circular membrane thickness *h* on the *C*–*q* relationships is shown in Figure 8.

From Figure 8, it can be seen that decreasing the circular membrane thickness *h* can decrease the output pressure range, and does not change the input capacitance range, so it can decrease the nonlinear strength of the *C*–*q* relationships.

### 4.4. The Effect of Changing Young’s Modulus of Elasticity E on C–q Relationships

**Figure 9:**
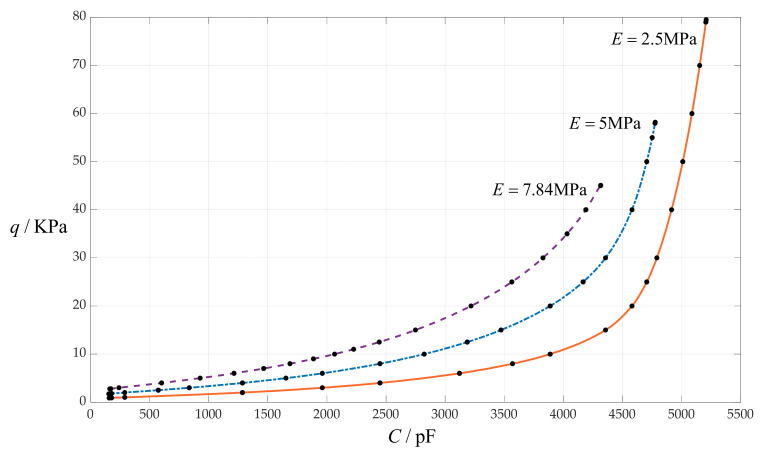
The effect of changing Young’s modulus of elasticity *E* on the *C*–*q* relationships when *a* = 100 mm, *g* = 20 mm, *h* = 1 mm, *t* = 0.1 mm, *ν* = 0.47 and *E* takes 7.84 MPa, 5 MPa and 2.5 MPa, respectively.

In this section, Young’s modulus of elasticity *E* takes 7.84 MPa, 5 MPa and 2.5 MPa, respectively, while the other design parameters remain unchanged, that is, the circular membrane radius *a* = 100 mm, the initially parallel gap *g* = 20 mm, circular membrane thickness *h* = 1 mm, insulator layer thickness *t* = 0.1 mm and Poisson’s ratio *v* = 0.47. In addition, the vacuum permittivity is still *ε*_0_ = 8.854 × 10^−3^ pF/mm, the relative permittivity of the insulator layer (polystyrene) is *ε*_r1_ = 2.7, and the air relative permittivity is *ε*_r2_ = 1.00053. The calculation results of the total capacitances *C* under different pressures *q* are listed in Table 1 when *E* = 7.84 MPa, in Table 7 when *E* = 5 MPa and in Table 8 when *E* = 2.5 MPa. The effect of changing Young’s modulus of elasticity *E* on the *C*–*q* relationships is shown in Figure 9.

From Figure 9, it can be seen that decreasing Young’s modulus of elasticity *E* can increase both the output pressure range and the input capacitance range, but it can also increase the nonlinear strength of the *C*–*q* relationships.

### 4.5. The Effect of Changing Poisson’s Ratio v on C–q Relationships

**Figure 10:**
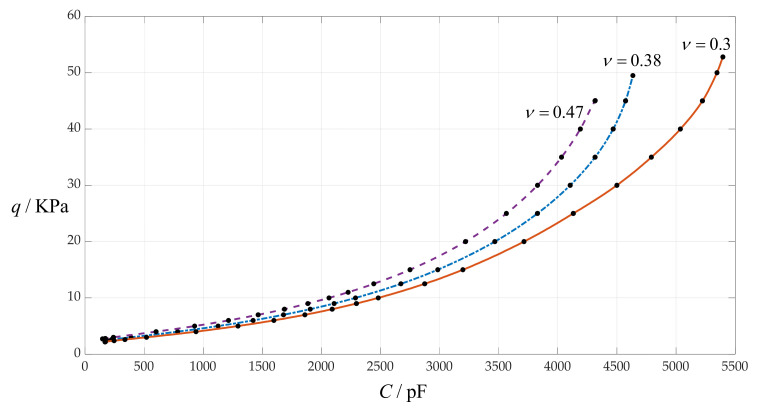
The effect of changing Poisson’s ratio *v* on the *C*–*q* relationships when *a* = 100 mm, *g* = 20 mm, *h* = 1 mm, *t* = 0.1 mm, *E* = 7.84 MPa and *v* takes 0.47, 0.38 and 0.3, respectively.

In this section, Poisson’s ratio *v* takes 0.47, 0.38 and 0.3, respectively, while the other design parameters remain unchanged, that is, the circular membrane radius *a* = 100 mm, the initially parallel gap *g* = 20 mm, circular membrane thickness *h* = 1 mm, insulator layer thickness *t* = 0.1 mm and Young’s modulus of elasticity *E* = 7.84 MPa. In addition, the vacuum permittivity is still *ε*_0_ = 8.854 × 10^−3^ pF/mm, the relative permittivity of the insulator layer (polystyrene) is *ε*_r1_ = 2.7 and the air relative permittivity is *ε*_r2_ = 1.00053. The calculation results of the total capacitances *C* under different pressures *q* are listed in Table 1 when *v* = 0.47, in Table 9 when *v* = 0.38 and in Table 10 when *v* = 0.3. The effect of changing Poisson’s ratio *v* on the *C*–*q* relationships is shown in Figure 10.

From Figure 10, it can be seen that decreasing Poisson’s ratio *v* can increase both the output pressure range and the input capacitance range, and it has little effect on the nonlinear strength of the *C*–*q* relationships.

### 4.6. The Effect of Changing Insulator Layer Thickness t on C–q Relationships

**Figure 11:**
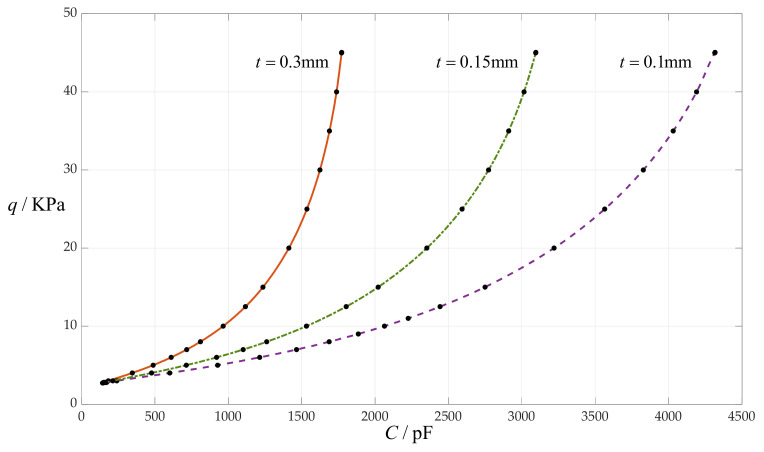
The effect of changing insulator layer thickness *t* on the *C*–*q* relationships when *a* = 100 mm, *g* = 20 mm, *h* = 1 mm, *E* = 7.84 MPa, *ν* = 0.47 and *t* takes 0.1 mm, 0.15 mm and 0.3 mm, respectively.

In this section, the insulator layer thickness *t* takes 0.1 mm, 0.15 mm and 0.3 mm, respectively, while the other design parameters remain unchanged, that is, the circular membrane radius *a* = 100 mm, the initially parallel gap *g* = 20 mm, circular membrane thickness *h* = 1 mm, Young’s modulus of elasticity *E* = 7.84 MPa and Poisson’s ratio *v* = 0.47. In addition, the vacuum permittivity is still *ε*_0_ = 8.854 × 10^−3^ pF/mm, the relative permittivity of the insulator layer (polystyrene) is *ε*_r1_ = 2.7 and the air relative permittivity is *ε*_r2_ = 1.00053. The calculation results of the total capacitances *C* under different pressures *q* are listed in Table 1 when *t* = 0.1 mm, in Table 11 when *t* = 0.15 mm and in Table 12 when *t* = 0.3 mm. The effect of changing the insulator layer thickness *t* on the *C*–*q* relationships is shown in Figure 11.

From Figure 11, it can be seen that decreasing the insulator layer thickness *t* can increase the input capacitance range and does not change the output pressure range, so it can decrease the nonlinear strength of the *C*–*q* relationships.

### 4.7. The Effect of Changing Circular Membrane Radius a on C–q Relationships

**Figure 12:**
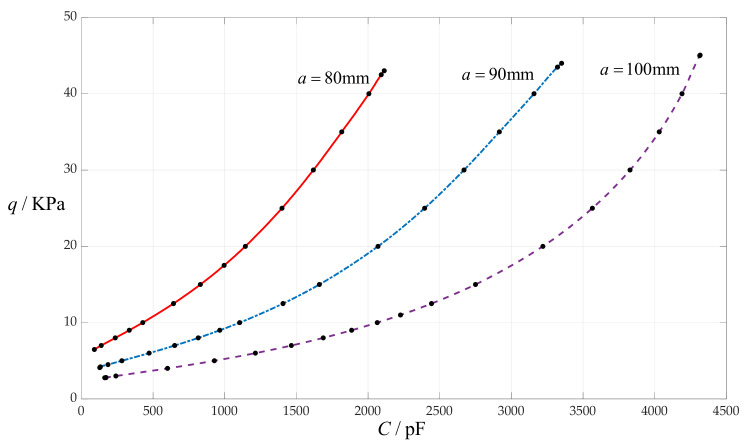
The effect of changing the circular membrane radius *a* on *C*–*q* relationships when *g* = 20 mm, *h* = 1 mm, *t* = 0.1 mm, *E* = 7.84 MPa, *ν* = 0.47 and *a* takes 100 mm, 90 mm and 80 mm, respectively.

In this section, the circular membrane radius *a* takes 100 mm, 90 mm and 80 mm, respectively, while the other design parameters remain unchanged, that is, the initially parallel gap *g* = 20 mm, circular membrane thickness *h* = 1 mm, insulator layer thickness *t* = 0.1 mm, Young’s modulus of elasticity *E* = 7.84 MPa and Poisson’s ratio *v* = 0.47. In addition, the vacuum permittivity is still *ε*_0_ = 8.854 × 10^−3^ pF/mm, the relative permittivity of the insulator layer (polystyrene) is *ε*_r1_ = 2.7 and the air relative permittivity is *ε*_r2_ = 1.00053. The calculation results of the total capacitances *C* under different pressures *q* are listed in Table 1 when *a* = 100 mm, in Table 13 when *a* = 90 mm and in Table 14 when *a* = 80 mm. The effect of changing the circular membrane radius *a* on the *C*–*q* relationships is shown in Figure 12.

From Figure 12, it can be seen that increasing the circular membrane radius *a* can increase the input capacitance range but can increase the output pressure range only a little bit, so it can decrease the nonlinear strength of the *C*–*q* relationships.

### 4.8. The Effect of Changing Number of Parallel Capacitors n on C–q Relationships

**Figure 13:**
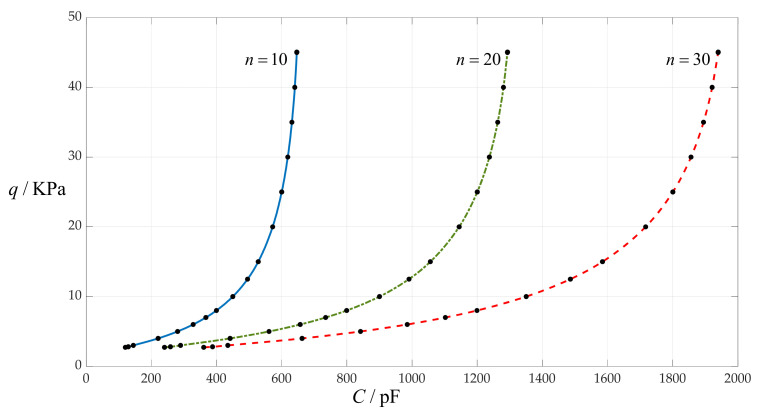
The effect of changing the number of parallel capacitors *n* on *C*–*q* relationships when *a* = 10 mm, *g* = 2 mm, *h* = 0.1 mm, *t* = 0.1 mm, *E* = 7.84 MPa, *ν* = 0.47 and *n* takes 10, 20 and 30, respectively.

The parallel use of many small sensors is a relatively common technical scheme, so here we consider the case of 10, 20 and 30 small capacitors in parallel, where each small capacitor is composed of a circular membrane with radius *a* = 10 mm and thickness *h* = 0.1 mm, and the initially parallel gap *g* = 2 mm. The other design parameters remain unchanged, that is, the insulator layer thickness is still *t* = 0.1 mm, Young’s modulus of elasticity is *E* = 7.84 MPa, Poisson’s ratio is *v* = 0.47, the vacuum permittivity is *ε*_0_ = 8.854 × 10^−3^ pF/mm, the relative permittivity of the insulator layer (polystyrene) is *ε*_r1_ = 2.7 and the air relative permittivity is *ε*_r2_ = 1.00053. The calculation results of the total capacitances *C* under different pressures *q* are listed in Table 15. The effect of changing the number of parallel capacitors *n* on the *C*–*q* relationships is shown in Figure 13.

From Figure 13, it can be seen that increasing the number of parallel capacitors *n* can increase the input capacitance range and does not change the output pressure range, so it can decrease the nonlinear strength of the *C*–*q* relationships.

## 5. Concluding Remarks

In this paper, the plate/membrane axisymmetric contact problem in circular touch mode capacitive pressure sensors is reformulated using a more accurate in-plane equilibrium equation, and a new and more accurate analytical solution is presented. On this basis, the design and numerical calibration theory for circular touch mode capacitive pressure sensors has been greatly improved and perfected. Specifically, the difference between the pressure–capacitance analytical relationships calculated by the new and previous analytical solutions increases gradually with the increase in the applied pressure, showing that in comparison with the previous analytical solution, the new analytical solution is indeed significantly improved, making the design and numerical calibration theory more accurate. In addition, for the first time, this paper illustrates in detail how to use analytical solutions and analytical relationships to design and numerically calibrate a circular touch mode capacitive pressure sensor with a specified pressure detecting range. The effect of changing design parameters on capacitance–pressure analytical relationships is also comprehensively investigated for the first time, which makes clear the direction of changing design parameters to meet the required or desired range of pressure or capacitance. The changing direction of design parameters can be summarized as follows. 

The decrease in the initially parallel gap *g* or Young’s modulus of elasticity *E* can increase both the output pressure range and the input capacitance range, but it can also increases the nonlinear strength of the *C*–*q* relationships. 

The decrease in Poisson’s ratio *v* can increase both the output pressure range and the input capacitance range, and it has little effect on the nonlinear strength of the *C*–*q* relationships.

The decrease in the insulator layer thickness *t* or the increase in the number of parallel capacitors *n* can increase the input capacitance range, and does not change the output pressure range, so it can decrease the nonlinear strength of the *C*–*q* relationships.

The decrease in the circular membrane thickness *h* can decrease the output pressure range and does not change the input capacitance range, so it can decrease the nonlinear strength of the *C*–*q* relationships.

The increase in the circular membrane radius *a* can increase the input capacitance range but can only increase the output pressure range a little bit, so it can decrease the nonlinear strength of the *C*–*q* relationships.

This study makes a very positive contribution to both membrane mechanics and its technical applications: a new and more accurate analytical solution of the plate/membrane contact problem is presented for the first time, and on this basis, the design and numerical calibration theory for circular touch mode capacitive pressure sensors has been greatly improved and perfected. The work presented here can provide a better support for the design and numerical calibration of circular touch mode capacitive pressure sensors.

## Figures and Tables

**Table 1:** The calculation results for *a* = 100 mm, *h* = 1 mm, *t* = 0.1 mm, *E* = 7.84 MPa, *ν* = 0.47, g = 20 mm.

*q*/KPa	*b/*mm	*b*_0_	*c*_0_	*d*_0_	*σ*_m_/MPa	*C*_1_*/*pF	*C*_2_*/*pF	*C/*pF
2.718	0.000	-	-	-	0.332	0.000	140.669	140.669
2.720	0.820	0.04591	0.04591	0.15227	0.338	0.505	148.023	148.528
2.800	5.035	0.04658	0.04122	0.14939	0.344	19.038	152.636	171.674
3.000	9.723	0.04820	0.04237	0.14748	0.360	71.006	169.488	240.494
4.000	21.580	0.05517	0.04810	0.14555	0.426	349.756	251.497	601.253
5.000	28.450	0.06093	0.05306	0.14529	0.484	607.885	319.743	927.629
6.000	33.339	0.06596	0.05742	0.14528	0.535	834.737	379.005	1213.742
7.000	37.095	0.07049	0.06136	0.14533	0.582	1033.427	431.535	1464.962
8.000	40.115	0.07465	0.06496	0.14539	0.626	1208.567	478.694	1687.260
9.000	42.621	0.07852	0.06830	0.14545	0.667	1364.256	521.413	1885.668
10.000	44.748	0.08215	0.07142	0.14552	0.707	1503.819	560.370	2064.189
11.000	46.586	0.08560	0.07437	0.14557	0.745	1629.896	596.086	2225.982
12.500	48.932	0.09047	0.07852	0.14564	0.799	1798.190	644.450	2442.639
15.000	52.060	0.09796	0.08486	0.14574	0.884	2035.489	713.531	2749.021
20.000	56.533	0.11131	0.09600	0.14586	1.040	2400.217	818.943	3219.159
25.000	59.654	0.12317	0.10579	0.14594	1.182	2672.563	892.028	3564.591
30.000	62.002	0.13403	0.114660	0.14598	1.315	2887.090	940.485	3827.574
35.000	63.856	0.14416	0.12287	0.14601	1.441	3062.374	968.835	4031.210
40.000	65.372	0.15372	0.13056	0.14602	1.562	3209.466	980.887	4190.353
45.000	66.642	0.16283	0.13787	0.14603	1.679	3335.431	978.746	4314.177
45.060	66.6562	0.16294	0.13795	0.14603	1.680	3336.835	979.311	4316.146

**Table 2:** The fitted analytical expressions of Functions 1, 2 and 3 in Figure 6.

Functions	*C*/pF	*q*/KPa	Analytical Expressions	Average Fitting Error Squares
Function 1	140.669~4316.146	2.718~45.06	*q* = 5.727382 × 10^−20^*C*^6^ − 6.342403 × 10^−16^*C*^5^ + 2.743908 × 10^−12^*C*^4^ − 5.418370 × 10^−9^*C*^3^ + 5.581685 × 10^−6^ *C*^2^ + 3.712548 × 10^−4^*C* + 2.592449	0.0028814
Function 2	140.669~2225.982	2.718~11	*q* = 3.766098 × 10^−3^*C* + 1.928101	0.1255083
Function 3	140.669~4316.146	2.718~45.06	*q* = 9.115659 × 10^−3^*C* − 3.585634	25.5704600

**Table 3:** The calculation results for *a* = 100 mm, *h* = 1 mm, *t* = 0.1 mm, *E* = 7.84 MPa, *ν* = 0.47, g = 10 mm.

*q*/KPa	*b/*mm	*b*_0_	*c*_0_	*d*_0_	*σ*_m_/MPa	*C/*pF
0.3512	0.000	0.01164	0.01058	0.07784	0.076	313.512
0.3514	0.895	0.01179	0.01064	0.07639	0.080	324.196
0.3530	2.093	0.01181	0.01063	0.07591	0.081	340.354
0.4000	11.378	0.01252	0.01113	0.07383	0.087	464.029
0.5000	20.431	0.01384	0.01227	0.07316	0.098	808.403
1.0000	39.389	0.01847	0.01648	0.07311	0.138	2099.409
2.5000	56.215	0.02655	0.02385	0.07349	0.211	3810.873
5.0000	65.407	0.03497	0.03139	0.07371	0.293	4655.640
7.5000	69.776	0.04121	0.03689	0.07381	0.356	4917.764
10.0000	72.503	0.04642	0.04143	0.07387	0.412	5050.278
20.0000	78.000	0.06256	0.05528	0.07396	0.593	5327.927
40.0000	82.233	0.08647	0.07544	0.07399	0.880	5599.954
60.0000	84.221	0.10623	0.09201	0.07399	1.128	5751.129
80.0000	85.440	0.12406	0.10705	0.07397	1.356	5851.602
100.0000	86.285	0.14074	0.12126	0.07395	1.572	5924.701
110.0000	86.621	0.14877	0.12816	0.07394	1.677	5954.636
110.2000	86.627	0.14893	0.12830	0.07394	1.680	5955.198

**Table 4:** The calculation results for *a* = 100 mm, *h* = 1 mm, *t* = 0.1 mm, *E* = 7.84 MPa, *ν* = 0.47 and g = 30 mm.

*q*/KPa	*b/*mm	*b*_0_	*c*_0_	*d*_0_	*σ*_m_/MPa	*C/*pF
8.702	0.000	0.09818	0.08636	0.23015	0.796	116.259
8.710	1.202	0.09905	0.08662	0.22651	0.805	130.806
9.100	6.357	0.10156	0.08779	0.22163	0.830	147.293
9.500	9.180	0.10393	0.08938	0.21996	0.854	190.312
10.000	11.898	0.10674	0.09142	0.21878	0.884	246.070
12.500	20.863	0.11919	0.10109	0.21672	1.020	526.564
15.000	26.686	0.12996	0.10973	0.21622	1.167	791.823
17.500	31.033	0.13965	0.11755	0.21606	1.256	1037.767
20.000	34.479	0.14856	0.12473	0.21602	1.363	1266.945
22.500	37.311	0.15689	0.13142	0.21602	1.464	1483.482
25.000	39.701	0.16476	0.13770	0.21603	1.561	1692.108
26.000	40.558	0.16779	0.14011	0.21604	1.599	1774.515
27.000	41.368	0.17077	0.14248	0.21605	1.636	1856.934
27.500	41.756	0.17224	0.14365	0.21365	1.650	1898.278
28.000	42.134	0.17370	0.14480	0.21606	1.673	1939.797
28.100	42.208	0.17399	0.14503	0.21606	1.677	1948.130
28.150	42.245	0.17413	0.14514	0.21606	1.679	1952.296
28.170	42.260	0.17419	0.14519	0.21606	1.680	1953.975

**Table 5:** The calculation results for *a* = 100 mm, g = 20 mm, *t* = 0.1 mm, *E* = 7.84 MPa, *ν* = 0.47 and *h* = 1.5 mm.

*q*/KPa	*b/*mm	*b*_0_	*c*_0_	*d*_0_	*σ*_m_/MPa	*C/*pF
4.08	0.820	0.04591	0.04591	0.15227	0.338	148.528
5.00	14.759	0.05071	0.04436	0.14631	0.383	362.457
6.00	21.580	0.05517	0.04810	0.14555	0.426	601.253
8.00	30.243	0.06267	0.05457	0.14527	0.501	1027.261
10.00	35.939	0.06903	0.06009	0.14531	0.566	1384.715
12.50	41.000	0.07597	0.06610	0.14542	0.640	1755.832
15.00	44.748	0.08215	0.07142	0.14552	0.707	2064.189
20.00	50.067	0.09305	0.08071	0.14568	0.828	2551.568
25.00	53.761	0.10262	0.08877	0.14579	0.938	2923.610
30.00	56.533	0.11131	0.09600	0.14586	1.040	3219.159
35.00	58.719	0.11935	0.10265	0.14591	1.136	3460.166
40.00	60.505	0.12689	0.10883	0.14595	1.227	3659.999
45.00	62.002	0.13403	0.11466	0.14598	1.315	3827.574
50.00	63.282	0.14085	0.12019	0.14600	1.400	3968.910
55.00	64.393	0.14740	0.12548	0.14601	1.482	4088.528
60.00	65.372	0.15372	0.13056	0.14602	1.562	4189.975
67.59	66.656	0.16294	0.13795	0.14603	1.680	4316.146

**Table 6:** The calculation results for *a* = 100 mm, g = 20 mm, *t* = 0.1 mm, *E* = 7.84 MPa, *ν* = 0.47 and *h* = 2 mm.

*q*/KPa	*b/*mm	*b*_0_	*c*_0_	*d*_0_	*σ*_m_/MPa	*C/*pF
5.44	0.820	0.04591	0.04591	0.15227	0.338	148.528
6.00	9.723	0.04820	0.04237	0.14748	0.360	240.494
8.00	21.580	0.05517	0.04810	0.14555	0.426	601.253
10.00	28.450	0.06093	0.05306	0.14529	0.484	927.629
12.50	34.364	0.06713	0.05844	0.14529	0.547	1279.574
15.00	38.681	0.07261	0.06319	0.14536	0.604	1579.385
20.00	44.748	0.08215	0.07142	0.14552	0.707	2064.189
25.00	48.932	0.09047	0.07852	0.14564	0.799	2442.639
30.00	52.060	0.09796	0.08486	0.14574	0.884	2749.021
40.00	56.533	0.11131	0.09600	0.14586	1.040	3219.159
50.00	59.654	0.12317	0.10579	0.14594	1.182	3564.591
60.00	62.002	0.13403	0.11466	0.14598	1.315	3827.574
70.00	63.856	0.14416	0.12287	0.14601	1.441	4031.210
80.00	65.372	0.15372	0.13056	0.14602	1.562	4190.353
90.00	66.642	0.16283	0.13787	0.14603	1.679	4314.177
90.12	66.656	0.16294	0.13795	0.14603	1.680	4316.146

**Table 7:** The calculation results for *a* = 100 mm, g = 20 mm, *h* = 1 mm, *t* = 0.1 mm, *ν* = 0.47 and *E* = 5 MPa.

*q*/KPa	*b/*mm	*b*_0_	*c*_0_	*d*_0_	*σ*_m_/MPa	*C/*pF
1.735	0.897	0.04591	0.04097	0.15221	0.215	155.165
1.800	5.720	0.04676	0.04134	0.14904	0.221	178.632
2.000	12.010	0.04925	0.04318	0.14686	0.236	290.047
2.500	20.893	0.05467	0.04767	0.14559	0.269	573.373
3.000	26.684	0.05932	0.05166	0.14532	0.298	835.366
4.000	34.451	0.06724	0.05853	0.14529	0.349	1285.266
5.000	39.671	0.07400	0.06440	0.14538	0.395	1653.415
6.000	43.528	0.08003	0.06960	0.14548	0.436	1960.710
8.000	48.994	0.09061	0.07864	0.14564	0.510	2448.583
10.000	52.785	0.09989	0.08648	0.14576	0.578	2822.750
12.500	56.236	0.11030	0.09517	0.14585	0.655	3186.919
15.000	58.828	0.11978	0.10300	0.14592	0.728	3472.338
20.000	62.547	0.13685	0.11695	0.14599	0.861	3888.116
25.000	65.148	0.15222	0.12936	0.14602	0.984	4167.118
30.000	67.105	0.16644	0.14075	0.14603	1.100	4356.798
40.000	69.913	0.19252	0.16148	0.14601	1.317	4580.665
50.000	71.873	0.21648	0.18040	0.14598	1.520	4705.456
55.000	72.657	0.22788	0.18938	0.14596	1.617	4751.286
58.000	73.081	0.23457	0.19465	0.14594	1.675	4775.727
58.200	73.108	0.23501	0.19500	0.14594	1.680	4777.261

**Table 8:** The calculation results for *a* = 100 mm, g = 20 mm, *h* = 1 mm, *t* = 0.1 mm, *ν* = 0.47 and *E* = 2.5 MPa.

*q*/KPa	*b/*mm	*b*_0_	*c*_0_	*d*_0_	*σ*_m_/MPa	*C/*pF
0.868	1.744	0.04598	0.04095	0.15113	0.108	158.298
0.900	5.720	0.04676	0.04134	0.14904	0.110	178.632
1.000	12.010	0.04925	0.04318	0.14686	0.118	290.047
2.000	34.451	0.06724	0.05853	0.14529	0.175	1285.266
3.000	43.528	0.08003	0.06960	0.14548	0.436	1960.710
4.000	48.994	0.09061	0.07864	0.14564	0.255	2448.583
6.000	55.628	0.10831	0.09351	0.14584	0.320	3121.327
8.000	59.701	0.12337	0.10595	0.14594	0.378	3569.879
10.000	62.547	0.13685	0.11695	0.14599	0.431	3888.116
15.000	67.105	0.16644	0.14075	0.14603	0.550	4356.798
20.000	69.913	0.19252	0.16148	0.14601	0.659	4580.665
25.000	71.873	0.21648	0.18040	0.14598	0.760	4705.456
30.000	73.347	0.23897	0.19812	0.14593	0.856	4790.991
40.000	75.457	0.28089	0.23116	0.14583	1.039	4915.433
50.000	76.932	0.31991	0.26202	0.14573	1.211	5009.968
60.000	78.045	0.35681	0.29135	0.14562	1.375	5087.531
70.000	78.930	0.39202	0.31948	0.14553	1.533	5153.463
79.000	79.591	0.42249	0.34395	0.14544	1.671	5205.312
79.500	79.625	0.42415	0.34529	0.14544	1.680	5208.021

**Table 9:** The calculation results for *a* = 100 mm, g = 20 mm, *h* = 1 mm, *t* = 0.1 mm, *E* = 7.84 MPa and *ν* = 0.38.

*q*/KPa	*b/*mm	*b*_0_	*c*_0_	*d*_0_	*σ*_m_/MPa	*C/*pF
2.392	0.379	0.04067	0.03586	0.15323	0.292	174.459
2.400	1.657	0.04073	0.03579	0.15215	0.293	180.222
2.500	6.282	0.04154	0.03614	0.14935	0.301	186.514
2.600	8.915	0.04234	0.03668	0.14831	0.308	224.696
3.000	15.811	0.04530	0.03893	0.14669	0.336	389.251
4.000	25.829	0.05153	0.04407	0.14584	0.397	783.394
5.000	32.123	0.05675	0.04848	0.14573	0.450	1126.314
6.000	36.673	0.06134	0.05237	0.14575	0.497	1421.968
7.000	40.190	0.06549	0.05588	0.14581	0.541	1679.219
8.000	43.027	0.06932	0.05910	0.14587	0.582	1905.663
9.000	45.385	0.07290	0.06209	0.14593	0.621	2107.173
10.000	47.390	0.07626	0.06489	0.14598	0.658	2288.223
12.500	51.338	0.08399	0.07126	0.14609	0.745	2672.042
15.000	54.293	0.09098	0.07696	0.14616	0.825	2983.817
20.000	58.520	0.10348	0.08701	0.14626	0.973	3466.839
25.000	61.470	0.11465	0.09586	0.14632	1.108	3827.439
30.000	63.689	0.12491	0.10390	0.14635	1.235	4104.314
35.000	65.441	0.13451	0.11136	0.14636	1.356	4315.009
40.000	66.872	0.14360	0.11838	0.14637	1.472	4468.178
45.000	68.071	0.15229	0.12506	0.14637	1.583	4572.718
49.500	68.999	0.15981	0.13082	0.14636	1.680	4634.432

**Table 10:** The calculation results for *a* = 100 mm, g = 20 mm, *h* = 1 mm, *t* = 0.1 mm, *E* = 7.84 MPa and *ν* = 0.3.

*q*/KPa	*b/*mm	*b*_0_	*c*_0_	*d*_0_	*σ*_m_/MPa	*C/*pF
2.173	0.792	0.03714	0.03235	0.15333	0.262	169.126
2.200	3.304	0.03735	0.03231	0.15146	0.264	172.501
2.400	9.935	0.03893	0.03323	0.14846	0.279	246.287
2.600	13.912	0.04041	0.03431	0.14748	0.293	337.604
3.000	19.641	0.04311	0.03642	0.14666	0.319	520.458
4.000	28.820	0.04886	0.04111	0.14616	0.376	938.829
5.000	34.754	0.05372	0.04513	0.14611	0.425	1294.335
6.000	39.075	0.05801	0.04869	0.14615	0.470	1597.681
7.000	42.427	0.06191	0.05190	0.14620	0.512	1860.274
8.000	45.135	0.06552	0.05485	0.14626	0.551	2090.900
9.000	47.389	0.06889	0.05759	0.14631	0.588	2296.061
10.000	49.306	0.07207	0.06016	0.14635	0.623	2480.588
12.500	53.084	0.07939	0.06601	0.14644	0.706	2873.643
15.000	55.914	0.08603	0.07125	0.14651	0.783	3196.845
20.000	59.962	0.09796	0.08050	0.14659	0.925	3713.862
25.000	62.787	0.10865	0.08868	0.14663	1.056	4130.838
30.000	64.912	0.11850	0.09612	0.14665	1.178	4499.176
35.000	66.588	0.12774	0.10304	0.14666	1.295	4791.569
40.000	67.956	0.13651	0.10957	0.14666	1.407	5036.617
45.000	69.103	0.14490	0.11579	0.14666	1.515	5223.193
50.000	70.082	0.15297	0.12176	0.14665	1.621	5346.050
52.800	70.572	0.15738	0.12500	0.14664	1.680	5379.050

**Table 11:** The calculation results for *a* = 100 mm, g = 20 mm, *h* = 1 mm, *E* = 7.84 MPa, *ν* = 0.47 and *t* = 0.15 mm.

*q*/KPa	*b/*mm	*b*_0_	*c*_0_	*d*_0_	*σ*_m_/MPa	*C/*pF
2.72	0.820	0.04591	0.04591	0.15227	0.338	146.585
2.80	5.035	0.04658	0.04122	0.14939	0.344	162.993
3.00	9.723	0.04820	0.04237	0.14748	0.360	213.413
4.00	21.580	0.05517	0.04810	0.14555	0.426	477.139
5.00	28.450	0.06093	0.05306	0.14529	0.484	713.705
6.00	33.339	0.06596	0.05742	0.14528	0.535	920.432
7.00	37.095	0.07049	0.06136	0.14533	0.582	1101.607
8.00	40.115	0.07465	0.06496	0.14539	0.626	1261.671
10.00	44.748	0.08215	0.07142	0.14552	0.707	1532.428
12.50	48.932	0.09047	0.07852	0.14564	0.799	1803.162
15.00	52.060	0.09796	0.08486	0.14574	0.884	2021.169
20.00	56.533	0.11131	0.09600	0.14586	1.040	2352.714
25.00	59.654	0.12317	0.10579	0.14594	1.182	2592.964
30.00	62.002	0.13403	0.11466	0.14598	1.315	2773.046
35.00	63.856	0.14416	0.12287	0.14601	1.441	2910.032
40.00	65.372	0.15372	0.13056	0.14602	1.562	3014.646
45.00	66.642	0.16283	0.13787	0.14603	1.679	3094.516
45.06	66.656	0.16294	0.13795	0.14603	1.680	3095.387

**Table 12:** The calculation results for *a* = 100 mm, g = 20 mm, *h* = 1 mm, *E* = 7.84 MPa, *ν* = 0.47 and *t* = 0.3 mm.

*q*/KPa	*b/*mm	*b*_0_	*c*_0_	*d*_0_	*σ*_m_/MPa	*C/*pF
2.72	0.820	0.04591	0.04591	0.15227	0.338	143.284
2.80	5.035	0.04658	0.04122	0.14939	0.344	151.505
3.00	9.723	0.04820	0.04237	0.14748	0.360	182.955
4.00	21.580	0.05517	0.04810	0.14555	0.426	345.800
5.00	28.450	0.06093	0.05306	0.14529	0.484	488.127
6.00	33.339	0.06596	0.05742	0.14528	0.535	610.728
7.00	37.095	0.07049	0.06136	0.14533	0.582	716.981
8.00	40.115	0.07465	0.06496	0.14539	0.626	809.911
10.00	44.748	0.08215	0.07142	0.14552	0.707	964.911
12.50	48.932	0.09047	0.07852	0.14564	0.799	1116.742
15.00	52.060	0.09796	0.08486	0.14574	0.884	1236.315
20.00	56.533	0.11131	0.09600	0.14586	1.040	1412.682
25.00	59.654	0.12317	0.10579	0.14594	1.182	1535.543
30.00	62.002	0.13403	0.11466	0.14598	1.315	1624.285
35.00	63.856	0.14416	0.12287	0.14601	1.441	1689.360
40.00	65.372	0.15372	0.13056	0.14602	1.562	1737.354
45.00	66.642	0.16283	0.13787	0.14603	1.679	1772.129
45.06	66.656	0.16294	0.13795	0.14603	1.680	1772.506

**Table 13:** The calculation results for g = 20 mm, *h* = 1 mm, *t* = 0.1 mm, *E* = 7.84 MPa, *ν* = 0.47 and *a* = 90 mm.

*q*/KPa	*b/*mm	*b*_0_	*c*_0_	*d*_0_	*σ*_m_/MPa	*C/*pF
19.46	0.556	0.12009	0.10423	0.25143	1.010	55.810
20.00	2.959	0.12217	0.10497	0.24678	1.030	66.442
22.00	6.667	0.12873	0.10930	0.24272	1.100	107.551
24.00	9.077	0.13467	0.11367	0.24125	1.166	148.419
26.00	10.982	0.14022	0.11788	0.24048	1.229	189.159
28.00	12.583	0.14545	0.12192	0.24002	1.290	229.332
30.00	13.969	0.15043	0.12579	0.23973	1.348	268.756
32.00	15.194	0.15519	0.12952	0.23954	1.405	307.370
34.00	16.289	0.15977	0.13311	0.23940	1.460	345.185
36.00	17.280	0.16419	0.13658	0.23931	1.514	382.246
38.00	18.184	0.16847	0.13994	0.23925	1.566	418.632
40.00	19.013	0.17263	0.14319	0.23920	1.618	454.440
42.49	19.957	0.17764	0.14712	0.23917	1.680	498.372

**Table 14:** The calculation results for g = 20 mm, *h* = 1 mm, *t* = 0.1 mm, *E* = 7.84 MPa, *ν* = 0.47 and *a* = 80 mm.

*q*/KPa	*b/*mm	*b*_0_	*c*_0_	*d*_0_	*σ*_m_/MPa	*C/*pF
6.48	0.570	0.07034	0.06226	0.18993	0.543	90.433
7.00	6.811	0.07326	0.06385	0.18428	0.571	138.438
8.00	12.066	0.07829	0.06770	0.18222	0.622	236.738
9.00	15.696	0.08281	0.07137	0.18150	0.669	334.167
10.00	18.556	0.08698	0.07481	0.18117	0.712	427.901
12.50	23.862	0.09628	0.08259	0.18090	0.813	642.502
15.00	27.662	0.10447	0.08946	0.18088	0.904	830.354
17.50	30.588	0.11191	0.09568	0.18093	0.988	995.958
20.00	32.944	0.11879	0.10140	0.18098	1.068	1143.713
25.00	36.556	0.13132	0.11175	0.18109	1.216	1399.378
30.00	39.245	0.14269	0.12102	0.18117	1.354	1618.568
35.00	41.354	0.15321	0.12951	0.18122	1.484	1816.202
40.00	43.070	0.16308	0.13742	0.18127	1.608	2005.539
42.50	43.818	0.16782	0.14120	0.18128	1.668	2092.215
43.02	43.965	0.16879	0.14197	0.18128	1.680	2112.884

**Table 15:** The calculation results for g = 2 mm, *h* = 0.1 mm, *t* = 0.1 mm, *E* = 7.84 MPa, *ν* = 0.47 and *a* = 10 mm.

*q*/KPa	*b/*mm	*b*_0_	*c*_0_	*d*_0_	*σ*_m_/MPa	*C/*pF		
						*n =* 10	*n =* 20	*n =* 30
2.72	0.082	0.04591	0.04591	0.15227	0.338	120.276	240.553	360.829
2.80	0.503	0.04658	0.04122	0.14939	0.344	129.318	258.635	387.953
3.00	0.972	0.04820	0.04237	0.14748	0.360	144.877	289.755	434.632
4.00	2.158	0.05517	0.04810	0.14555	0.426	220.875	441.749	662.624
5.00	2.845	0.06093	0.05306	0.14529	0.484	280.673	561.346	842.019
6.00	3.334	0.06596	0.05742	0.14528	0.535	328.477	656.955	985.432
7.00	3.709	0.07049	0.06136	0.14533	0.582	367.438	734.877	1102.315
8.00	4.012	0.07465	0.06496	0.14539	0.626	399.742	799.484	1199.226
10.00	4.475	0.08215	0.07142	0.14552	0.707	450.097	900.194	1350.292
12.50	4.893	0.09047	0.07852	0.14564	0.799	495.312	990.624	1485.936
15.00	5.206	0.09796	0.08486	0.14574	0.884	528.158	1056.316	1584.474
20.00	5.653	0.11131	0.09600	0.14586	1.040	572.318	1144.636	1716.954
25.00	5.965	0.12317	0.10579	0.14594	1.182	600.135	1200.271	1800.406
30.00	6.200	0.13403	0.11466	0.14598	1.315	618.720	1237.440	1856.160
35.00	6.386	0.14416	0.12287	0.14601	1.441	631.493	1262.985	1894.478
40.00	6.537	0.15372	0.13056	0.14602	1.562	640.340	1280.680	1921.020
45.00	6.664	0.16283	0.13787	0.14603	1.679	646.431	1292.861	1939.292
45.06	6.666	0.16294	0.13795	0.14603	1.680	646.495	1292.991	1939.486

## Data Availability

The original contributions presented in the study are included in the article, further inquiries can be directed to the corresponding author/s.

## Appendix A

$$\begin{array}{l} b_{1}=-\frac{1}{\beta \left(Q^{2}\eta^{2}{d_{1}}^{2}-8\beta^{2}{b_{0}}^{2}{d_{1}}^{2}+Q^{2}\eta^{2}-4\beta^{2}{b_{0}}^{2}\right)}(4\beta^{2}{b_{0}}^{2}c_{0}{d_{1}}^{4}-Q^{2}\eta^{2}c_{0}{d_{1}}^{4}\\ +2Q^{2}\beta^{2}\eta b_{0}{d_{1}}^{2}+Q^{2}\eta^{2}b_{0}{d_{1}}^{2}-2Q^{2}\eta^{2}c_{0}{d_{1}}^{2}-8\beta^{2}{b_{0}}^{3}{d_{1}}^{2}+8\beta^{2}{b_{0}}^{2}c_{0}{d_{1}}^{2}+2Q^{2}\beta^{2}\eta b_{0}\\ +Q^{2}\eta^{2}b_{0}-Q^{2}\eta^{2}c_{0}-4\beta^{2}{b_{0}}^{3}+4\beta^{2}{b_{0}}^{2}c_{0}) \end{array}$$

$$\begin{array}{l} b_{2}=-\frac{1}{2\beta \left(Q^{2}\eta^{2}{d_{1}}^{2}-8\beta^{2}{b_{0}}^{2}d_{1}+Q^{2}\eta^{2}-4\beta^{2}{b_{0}}^{2}\right)}(-8Q^{2}\eta^{2}c_{0}{d_{1}}^{3}d_{2}-Q^{2}\eta^{2}c_{1}{d_{1}}^{4}\\ +32\beta^{2}{b_{0}}^{2}c_{0}{d_{1}}^{3}d_{2}+4\beta^{2}{b_{0}}^{2}c_{1}{d_{1}}^{4}+4Q^{2}\beta^{3}b_{0}{d_{1}}^{2}+16Q^{2}\beta^{2}\eta b_{0}d_{1}d_{2}+2Q^{2}\beta \eta^{2}b_{1}d_{1}d_{2}\\ -40\beta^{3}{b_{0}}^{2}b_{1}d_{1}d_{2}-4\beta^{3}b_{0}{b_{1}}^{2}{d_{1}}^{2}+2Q^{2}\beta \eta b_{0}{d_{1}}^{2}+2Q^{2}\eta^{2}b_{0}d_{1}d_{2}+2Q^{2}\eta^{2}b_{1}{d_{1}}^{2}-8Q^{2}\eta^{2}c_{0}d_{1}d_{2}\\ -2Q^{2}\eta^{2}c_{1}{d_{1}}^{2}-40\beta^{2}{b_{0}}^{3}d_{1}d_{2}-24\beta^{2}{b_{0}}^{2}b_{1}{d_{1}}^{2}+32\beta^{2}{b_{0}}^{2}c_{0}d_{1}d_{2}+8\beta^{2}{b_{0}}^{2}c_{1}{d_{1}}^{2}+4Q^{2}\beta^{3}b_{0}\\ -4\beta {b_{0}}^{3}{d_{1}}^{2}+2Q^{2}\beta \eta b_{0}+2Q^{2}\eta^{2}b_{1}-Q^{2}\eta^{2}c_{1}-8\beta^{2}{b_{0}}^{2}b_{1}+4\beta^{2}{b_{0}}^{2}c_{1}) \end{array}$$

$$\begin{array}{l} b_{3}=-\frac{1}{3\beta \left(Q^{2}\eta^{2}{d_{1}}^{2}-8\beta^{2}{b_{0}}^{2}{d_{1}}^{2}+Q^{2}\eta^{2}-4\beta^{2}{b_{0}}^{2}\right)}(-12Q^{2}\eta^{2}c_{0}{d_{1}}^{3}d_{3}-24Q^{2}\eta^{2}c_{0}{d_{1}}^{2}{d_{2}}^{2}\\ -8Q^{2}\eta^{2}c_{1}{d_{1}}^{3}d_{2}-Q^{2}\eta^{2}c_{2}{d_{1}}^{4}+48\beta^{2}{b_{0}}^{2}c_{0}{d_{1}}^{3}d_{3}+96\beta^{2}{b_{0}}^{2}c_{0}{d_{1}}^{2}{d_{2}}^{2}+32\beta^{2}{b_{0}}^{2}c_{1}{d_{1}}^{3}d_{2}+4\beta^{2}{b_{0}}^{2}c_{2}{d_{1}}^{4}\\ +24Q^{2}\beta^{3}b_{0}d_{1}d_{2}+36Q^{2}\beta^{2}\eta b_{0}d_{1}d_{3}+24Q^{2}\beta^{2}\eta b_{0}{d_{2}}^{2}+6Q^{2}\beta \eta^{2}b_{2}d_{1}d_{2}-72\beta^{3}{b_{0}}^{2}b_{1}d_{1}d_{3}\\ -48\beta^{3}{b_{0}}^{2}b_{1}{d_{2}}^{2}-72\beta^{3}{b_{0}}^{2}b_{2}d_{1}d_{2}-24\beta^{3}b_{0}{b_{1}}^{2}d_{1}d_{2}-12\beta^{3}b_{0}b_{1}b_{2}{d_{1}}^{2}+6Q^{2}\beta^{2}b_{0}{d_{1}}^{2}\\ +12Q^{2}\beta \eta b_{0}d_{1}d_{2}+6Q^{2}\eta^{2}b_{1}d_{1}d_{2}+3Q^{2}\eta^{2}b_{2}{d_{1}}^{2}-12Q^{2}\eta^{2}c_{0}d_{1}d_{3}-8Q^{2}\eta^{2}c_{0}{d_{2}}^{2}\\ -8Q^{2}\eta^{2}c_{1}d_{1}d_{2}-2Q^{2}\eta^{2}c_{2}{d_{1}}^{2}-72\beta^{2}{b_{0}}^{3}d_{1}d_{3}-48\beta^{2}{b_{0}}^{3}{d_{2}}^{2}-120\beta^{2}{b_{0}}^{2}b_{1}d_{1}d_{2}\\ -36\beta^{2}{b_{0}}^{2}b_{2}{d_{1}}^{2}+48\beta^{2}{b_{0}}^{2}c_{0}d_{1}d_{3}+32\beta^{2}{b_{0}}^{2}c_{0}{d_{2}}^{2}+32\beta^{2}{b_{0}}^{2}c_{1}d_{1}d_{2}+8\beta^{2}{b_{0}}^{2}c_{2}{d_{1}}^{2}\\ -12\beta^{2}b_{0}{b_{1}}^{2}{d_{1}}^{2}-24\beta {b_{0}}^{3}d_{1}d_{2}-12\beta {b_{0}}^{2}b_{1}{d_{1}}^{2}+6Q^{2}\beta^{2}b_{0}+3Q^{2}\eta^{2}b_{2}-Q^{2}\eta^{2}c_{2}\\ -12\beta^{2}{b_{0}}^{2}b_{2}+4\beta^{2}{b_{0}}^{2}c_{2}) \end{array}$$

$$\begin{array}{l} b_{4}=-\frac{1}{4\beta \left(Q^{2}\eta^{2}{d_{1}}^{2}-8\beta^{2}{b_{0}}^{2}{d_{1}}^{2}+Q^{2}\eta^{2}-4\beta^{2}{b_{0}}^{2}\right)}(-16Q^{2}\eta^{2}c_{0}{d_{1}}^{3}d_{4}-72Q^{2}\eta^{2}c_{0}{d_{1}}^{2}d_{2}d_{3}\\ -32Q^{2}\eta^{2}c_{0}d_{1}{d_{2}}^{3}-12Q^{2}\eta^{2}c_{1}{d_{1}}^{3}d_{3}-24Q^{2}\eta^{2}c_{1}{d_{1}}^{2}{d_{2}}^{2}-8Q^{2}\eta^{2}c_{2}{d_{1}}^{3}d_{2}-Q^{2}\eta^{2}c_{3}{d_{1}}^{4}\\ +64\beta^{2}{b_{0}}^{2}c_{0}{d_{1}}^{3}d_{4}+288\beta^{2}{b_{0}}^{2}c_{0}{d_{1}}^{2}d_{2}d_{3}+128\beta^{2}{b_{0}}^{2}c_{0}d_{1}{d_{2}}^{3}+48\beta^{2}{b_{0}}^{2}c_{1}{d_{1}}^{3}d_{3}+96\beta^{2}{b_{0}}^{2}c_{1}{d_{1}}^{2}{d_{2}}^{2}\\ +32\beta^{2}{b_{0}}^{2}c_{2}{d_{1}}^{3}d_{2}+4\beta^{2}{b_{0}}^{2}c_{3}{d_{1}}^{4}+48Q^{2}\beta^{3}b_{0}d_{1}d_{3}+32Q^{2}\beta^{3}b_{0}{d_{2}}^{2}64Q^{2}\beta^{2}\eta b_{0}d_{1}d_{4}\\ +96Q^{2}\beta^{2}\eta b_{0}d_{2}d_{3}-4Q^{2}\beta \eta^{2}b_{1}d_{1}d_{4}-6Q^{2}\beta \eta^{2}b_{1}d_{2}d_{3}+6Q^{2}\beta \eta^{2}b_{2}d_{1}d_{3}+4Q^{2}\beta \eta^{2}b_{2}{d_{2}}^{2}\\ +10Q^{2}\beta \eta^{2}b_{3}d_{1}d_{2}-112\beta^{3}{b_{0}}^{2}b_{1}d_{1}d_{4}-168\beta^{3}{b_{0}}^{2}b_{1}d_{2}d_{3}-120\beta^{3}{b_{0}}^{2}b_{2}d_{1}d_{3}-80\beta^{3}{b_{0}}^{2}b_{2}{d_{2}}^{2}\\ -104\beta^{3}{b_{0}}^{2}b_{3}d_{1}d_{2}-48\beta^{3}b_{0}{b_{1}}^{2}d_{1}d_{3}-32\beta^{3}b_{0}{b_{1}}^{2}{d_{2}}^{2}-64\beta^{3}b_{0}b_{1}b_{2}d_{1}d_{2}+6Q^{2}\eta^{2}b_{1}d_{1}d_{3}\\ -16\beta^{3}b_{0}b_{1}b_{3}{d_{1}}^{2}-8\beta^{3}b_{0}{b_{2}}^{2}{d_{1}}^{2}+32Q^{2}\beta^{2}b_{0}d_{1}d_{2}+24Q^{2}\beta \eta b_{0}d_{1}d_{3}+16Q^{2}\beta \eta b_{0}{d_{2}}^{2}\\ -4Q^{2}\eta^{2}b_{0}d_{1}d_{4}-6Q^{2}\eta^{2}b_{0}d_{2}d_{3}+4Q^{2}\eta^{2}b_{1}{d_{2}}^{2}+10Q^{2}\eta^{2}b_{2}d_{1}d_{2}+4Q^{2}\eta^{2}b_{3}{d_{1}}^{2}-16Q^{2}\eta^{2}c_{0}d_{1}d_{4}\\ -24Q^{2}\eta^{2}c_{0}d_{2}d_{3}-12Q^{2}\eta^{2}c_{1}d_{1}d_{3}-8Q^{2}\eta^{2}c_{1}{d_{2}}^{2}-8Q^{2}\eta^{2}c_{2}d_{1}d_{2}-2Q^{2}\eta^{2}c_{3}-112\beta^{2}{b_{0}}^{3}d_{1}d_{4}\\ -168\beta^{2}{b_{0}}^{3}d_{2}d_{3}-216\beta^{2}{b_{0}}^{2}b_{1}d_{1}d_{3}-144\beta^{2}{b_{0}}^{2}b_{1}{d_{2}}^{2}-168\beta^{2}{b_{0}}^{2}b_{2}d_{1}d_{2}-48\beta^{2}{b_{0}}^{2}b_{3}{d_{1}}^{2}\\ +64\beta^{2}{b_{0}}^{2}c_{0}d_{1}d_{4}+96\beta^{2}{b_{0}}^{2}c_{0}d_{2}d_{3}+48\beta^{2}{b_{0}}^{2}c_{1}d_{1}d_{3}+32\beta^{2}{b_{0}}^{2}c_{1}{d_{2}}^{2}+32\beta^{2}{b_{0}}^{2}c_{2}d_{1}d_{2}\\ +8\beta^{2}{b_{0}}^{2}c_{3}{d_{1}}^{2}-64\beta^{2}b_{0}{b_{1}}^{2}d_{1}d_{2}-32\beta^{2}b_{0}b_{1}b_{2}{d_{1}}^{2}+2Q^{2}\beta b_{0}{d_{1}}^{2}-48\beta {b_{0}}^{3}d_{1}d_{3}-32\beta {b_{0}}^{3}{d_{2}}^{2}\\ -64\beta {b_{0}}^{2}b_{1}d_{1}d_{2}-16\beta {b_{0}}^{2}b_{2}{d_{1}}^{2}-8\beta b_{0}{b_{1}}^{2}{d_{1}}^{2}+4Q^{2}\eta^{2}b_{3}-Q^{2}\eta^{2}c_{3}-16\beta^{2}{b_{0}}^{2}b_{3}\\ +4\beta^{2}{b_{0}}^{2}c_{3}+2Q^{2}\beta b_{0}+{d_{1}}^{2}) \end{array}$$

$$\begin{array}{l} b_{5}=-\frac{1}{5\beta \left(Q^{2}\eta^{2}{d_{1}}^{2}-8\beta^{2}{b_{0}}^{2}{d_{1}}^{2}+Q^{2}\eta^{2}-4\beta^{2}{b_{0}}^{2}\right)}(-20Q^{2}\eta^{2}c_{0}{d_{1}}^{3}d_{5}-96Q^{2}\eta^{2}c_{0}{d_{1}}^{2}d_{2}d_{4}\\ -54Q^{2}\eta^{2}c_{0}{d_{1}}^{2}{d_{3}}^{2}-144Q^{2}\eta^{2}c_{0}d_{1}{d_{2}}^{2}d_{3}-16Q^{2}\eta^{2}c_{0}{d_{2}}^{4}-16Q^{2}\eta^{2}c_{1}{d_{1}}^{3}d_{4}-72Q^{2}\eta^{2}c_{1}{d_{1}}^{2}d_{2}d_{3}\\ -32Q^{2}\eta^{2}c_{1}d_{1}{d_{2}}^{3}-12Q^{2}\eta^{2}c_{2}{d_{1}}^{3}d_{3}-24Q^{2}\eta^{2}c_{2}{d_{1}}^{2}{d_{2}}^{2}-8Q^{2}\eta^{2}c_{3}{d_{1}}^{3}d_{2}-Q^{2}\eta^{2}c_{4}{d_{1}}^{4}\\ +80\beta^{2}{b_{0}}^{2}c_{0}{d_{1}}^{3}d_{5}+384\beta^{2}{b_{0}}^{2}c_{0}{d_{1}}^{2}d_{2}d_{4}+216\beta^{2}{b_{0}}^{2}c_{0}{d_{1}}^{2}{d_{3}}^{2}+576\beta^{2}{b_{0}}^{2}c_{0}d_{1}{d_{2}}^{2}d_{3}\\ +64\beta^{2}{b_{0}}^{2}c_{0}{d_{2}}^{4}+64\beta^{2}{b_{0}}^{2}c_{1}{d_{1}}^{3}d_{4}+288\beta^{2}{b_{0}}^{2}c_{1}{d_{1}}^{2}d_{2}d_{3}+128\beta^{2}{b_{0}}^{2}c_{1}d_{1}{d_{2}}^{3}+48\beta^{2}\\ +96\beta^{2}{b_{0}}^{2}c_{2}{d_{1}}^{2}{d_{2}}^{2}+32\beta^{2}{b_{0}}^{2}c_{3}{d_{1}}^{3}d_{2}+4\beta^{2}{b_{0}}^{2}c_{4}{d_{1}}^{4}+80Q^{2}\beta^{3}b_{0}d_{1}d_{4}+120Q^{2}\beta^{3}b_{0}d_{2}d_{3}\\ +100Q^{2}\beta^{2}\eta b_{0}d_{1}d_{5}+160Q^{2}\beta^{2}\eta b_{0}d_{2}d_{4}+90Q^{2}\beta^{2}\eta b_{0}{d_{3}}^{2}-10Q^{2}\beta \eta^{2}b_{1}d_{1}d_{5}\\ -16Q^{2}\beta \eta^{2}b_{1}d_{2}d_{4}-9Q^{2}\beta \eta^{2}b_{1}{d_{3}}^{2}+4Q^{2}\beta \eta^{2}b_{2}d_{1}d_{4}+6Q^{2}\beta \eta^{2}b_{2}d_{2}d_{3}+12Q^{2}\beta \eta^{2}b_{3}d_{1}d_{3}\\ +8Q^{2}\beta \eta^{2}b_{3}{d_{2}}^{2}+14Q^{2}\beta \eta^{2}b_{4}d_{1}d_{2}-160\beta^{3}{b_{0}}^{2}b_{1}d_{1}d_{5}-256\beta^{3}{b_{0}}^{2}b_{1}d_{2}d_{4}-144\beta^{3}{b_{0}}^{2}b_{1}{d_{3}}^{2}\\ +{b_{0}}^{2}c_{2}{d_{1}}^{3}d_{3}-176\beta^{3}{b_{0}}^{2}b_{2}d_{1}d_{4}-264\beta^{3}{b_{0}}^{2}b_{2}d_{2}d_{3}-168\beta^{3}{b_{0}}^{2}b_{3}d_{1}d_{3}-112\beta^{3}{b_{0}}^{2}b_{3}{d_{2}}^{2}\\ -136\beta^{3}{b_{0}}^{2}b_{4}d_{1}d_{2}-80\beta^{3}b_{0}{b_{1}}^{2}d_{1}d_{4}-120\beta^{3}b_{0}{b_{1}}^{2}d_{2}d_{3}-120\beta^{3}b_{0}b_{1}b_{2}d_{1}d_{3}-80\beta^{3}b_{0}b_{1}b_{2}{d_{2}}^{2}\\ -80\beta^{3}b_{0}b_{1}b_{3}d_{1}d_{2}-20\beta^{3}b_{0}b_{1}b_{4}{d_{1}}^{2}-40\beta^{3}b_{0}{b_{2}}^{2}d_{1}d_{2}-20\beta^{3}b_{0}b_{2}b_{3}{d_{1}}^{2}+60Q^{2}\beta^{2}b_{0}d_{1}d_{3}\\ +40Q^{2}\beta^{2}b_{0}{d_{2}}^{2}+40Q^{2}\beta \eta b_{0}d_{1}d_{4}+60Q^{2}\beta \eta b_{0}d_{2}d_{3}-10Q^{2}\eta^{2}b_{0}d_{1}d_{5}-16Q^{2}\eta^{2}b_{0}d_{2}d_{4}\\ -9Q^{2}\eta^{2}b_{0}{d_{3}}^{2}4Q^{2}\eta^{2}b_{1}d_{1}d_{4}+6Q^{2}\eta^{2}b_{1}d_{2}d_{3}+12Q^{2}\eta^{2}b_{2}d_{1}d_{3}+8Q^{2}\eta^{2}b_{2}{d_{2}}^{2}+14Q^{2}\eta^{2}b_{3}d_{1}d_{2}\\ +5Q^{2}\eta^{2}b_{4}{d_{1}}^{2}-20Q^{2}\eta^{2}c_{0}d_{1}d_{5}-32Q^{2}\eta^{2}c_{0}d_{2}d_{4}-18Q^{2}\eta^{2}c_{0}{d_{3}}^{2}-16Q^{2}\eta^{2}c_{1}d_{1}d_{4}\\ -24Q^{2}\eta^{2}c_{1}d_{2}d_{3}-12Q^{2}\eta^{2}c_{2}d_{1}d_{3}-8Q^{2}\eta^{2}c_{2}{d_{2}}^{2}-8Q^{2}\eta^{2}c_{3}d_{1}d_{2}-2Q^{2}\eta^{2}c_{4}{d_{1}}^{2}\\ -160\beta^{2}{b_{0}}^{3}d_{1}d_{5}-256\beta^{2}{b_{0}}^{3}d_{2}d_{4}-144\beta^{2}{b_{0}}^{3}{d_{3}}^{2}-336\beta^{2}{b_{0}}^{2}b_{1}d_{1}d_{4}-504\beta^{2}{b_{0}}^{2}b_{1}d_{2}d_{3}\\ -288\beta^{2}{b_{0}}^{2}b_{2}d_{1}d_{3}-192\beta^{2}{b_{0}}^{2}b_{2}{d_{2}}^{2}-216\beta^{2}-60\beta^{2}{b_{0}}^{2}b_{4}{d_{1}}^{2}+80\beta^{2}{b_{0}}^{2}c_{0}d_{1}d_{5}\\ +128\beta^{2}{b_{0}}^{2}c_{0}d_{2}d_{4}+72\beta^{2}{b_{0}}^{2}c_{0}{d_{3}}^{2}+64\beta^{2}{b_{0}}^{2}c_{1}d_{1}d_{4}+96\beta^{2}{b_{0}}^{2}c_{1}d_{2}d_{3}+48\beta^{2}{b_{0}}^{2}c_{2}d_{1}d_{3}\\ +32\beta^{2}{b_{0}}^{2}c_{2}{d_{2}}^{2}+32\beta^{2}{b_{0}}^{2}c_{3}d_{1}d_{2}+8\beta^{2}{b_{0}}^{2}c_{4}{d_{1}}^{2}-120\beta^{2}b_{0}{b_{1}}^{2}d_{1}d_{3}-80\beta^{2}b_{0}{b_{1}}^{2}{d_{2}}^{2}\\ -160\beta^{2}b_{0}b_{1}b_{2}d_{1}d_{2}-40\beta^{2}b_{0}b_{1}b_{3}{d_{1}}^{2}-20\beta^{2}b_{0}{b_{2}}^{2}{d_{1}}^{2}+10Q^{2}\beta b_{0}d_{1}d_{2}-80\beta {b_{0}}^{3}d_{1}d_{4}\\ -120\beta {b_{0}}^{3}d_{2}d_{3}-120\beta {b_{0}}^{2}b_{1}d_{1}d_{3}-80\beta {b_{0}}^{2}b_{1}{d_{2}}^{2}-80\beta {b_{0}}^{2}b_{2}d_{1}d_{2}-20\beta {b_{0}}^{2}b_{3}{d_{1}}^{2}\\ +{b_{0}}^{2}b_{3}d_{1}d_{2}-40\beta b_{0}{b_{1}}^{2}d_{1}d_{2}-20\beta b_{0}b_{1}b_{2}{d_{1}}^{2}+5Q^{2}\eta^{2}b_{4}-Q^{2}\eta^{2}c_{4}-20\beta^{2}{b_{0}}^{2}b_{4}+4\beta^{2}{b_{0}}^{2}c_{4}) \end{array}$$

$$\begin{array}{l} b_{6}=-\frac{1}{6\beta \left(Q^{2}\eta^{2}{d_{1}}^{2}-8\beta^{2}{b_{0}}^{2}{d_{1}}^{2}+Q^{2}\eta^{2}-4\beta^{2}{b_{0}}^{2}\right)}(-24Q^{2}\eta^{2}c_{0}{d_{1}}^{3}d_{6}-96Q^{2}\eta^{2}c_{0}{d_{2}}^{3}d_{3}\\ -20Q^{2}\eta^{2}c_{1}{d_{1}}^{3}d_{5}-54Q^{2}\eta^{2}c_{1}{d_{1}}^{2}{d_{3}}^{2}-32Q^{2}\eta^{2}c_{2}d_{1}{d_{2}}^{3}-24Q^{2}\eta^{2}c_{3}{d_{1}}^{2}{d_{2}}^{2}-8Q^{2}\eta^{2}c_{4}{d_{1}}^{3}d_{2}\\ +384\beta^{2}{b_{0}}^{2}c_{0}{d_{2}}^{3}d_{3}+128\beta^{2}{b_{0}}^{2}c_{2}d_{1}{d_{2}}^{3}-30Q^{2}\eta^{2}b_{0}d_{2}d_{5}-36Q^{2}\eta^{2}b_{0}d_{3}d_{4}+12Q^{2}\eta^{2}b_{2}d_{1}d_{4}\\ +18Q^{2}\eta^{2}b_{2}d_{2}d_{3}-24Q^{2}\eta^{2}c_{0}d_{1}d_{6}-32Q^{2}\eta^{2}c_{1}d_{2}d_{4}-12Q^{2}\eta^{2}c_{3}d_{1}d_{3}-8Q^{2}\eta^{2}c_{4}d_{1}d_{2}\\ +96\beta^{2}{b_{0}}^{2}c_{0}d_{1}d_{6}+128\beta^{2}{b_{0}}^{2}c_{1}d_{2}d_{4}+48\beta^{2}{b_{0}}^{2}c_{3}d_{1}d_{3}+32\beta^{2}{b_{0}}^{2}c_{4}d_{1}d_{2}-16Q^{2}\eta^{2}c_{2}{d_{1}}^{3}d_{4}\\ +12Q^{2}\beta \eta^{2}b_{4}{d_{2}}^{2}-18Q^{2}\eta^{2}b_{0}d_{1}d_{6}+18Q^{2}\eta^{2}b_{3}d_{1}d_{3}+18Q^{2}\eta^{2}b_{4}d_{1}d_{2}-40Q^{2}\eta^{2}c_{0}d_{2}d_{5}\\ -48Q^{2}\eta^{2}c_{0}d_{3}d_{4}-20Q^{2}\eta^{2}c_{1}d_{1}d_{5}-16Q^{2}\eta^{2}c_{2}d_{1}d_{4}-24Q^{2}\eta^{2}c_{2}d_{2}d_{3}+160\beta^{2}{b_{0}}^{2}c_{0}d_{2}d_{5}\\ +192\beta^{2}{b_{0}}^{2}c_{0}d_{3}d_{4}+80\beta^{2}{b_{0}}^{2}c_{1}d_{1}d_{5}+64\beta^{2}{b_{0}}^{2}c_{2}d_{1}d_{4}+96\beta^{2}{b_{0}}^{2}c_{2}d_{2}d_{3}+12\beta b_{0}Q^{2}{d_{2}}^{2}\\ -108\beta {b_{0}}^{3}{d_{3}}^{2}-72\beta b_{0}{b_{1}}^{2}d_{1}d_{3}-48\beta b_{0}{b_{1}}^{2}{d_{2}}^{2}+6Q^{2}\eta^{2}b_{5}-Q^{2}\eta^{2}c_{5}-24\beta^{2}{b_{0}}^{2}b_{5}\\ +4\beta^{2}{b_{0}}^{2}c_{5}-16Q^{2}\eta^{2}c_{1}{d_{2}}^{4}-Q^{2}\eta^{2}c_{5}{d_{1}}^{4}+64\beta^{2}{b_{0}}^{2}c_{1}{d_{2}}^{4}+4\beta^{2}{b_{0}}^{2}c_{5}{d_{1}}^{4}+108\beta^{3}b_{0}Q^{2}{d_{3}}^{2}\\ -216\beta^{3}{b_{0}}^{2}b_{2}{d_{3}}^{2}-144\beta^{3}{b_{0}}^{2}b_{4}{d_{2}}^{2}-108\beta^{3}b_{0}{b_{1}}^{2}{d_{3}}^{2}-48\beta^{3}b_{0}{b_{2}}^{2}{d_{2}}^{2}-12\beta^{3}b_{0}{b_{3}}^{2}{d_{1}}^{2}\\ +12Q^{2}\eta^{2}b_{3}{d_{2}}^{2}+6Q^{2}\eta^{2}b_{5}{d_{1}}^{2}-18Q^{2}\eta^{2}c_{1}{d_{3}}^{2}-8Q^{2}\eta^{2}c_{3}{d_{2}}^{2}-2Q^{2}\eta^{2}c_{5}{d_{1}}^{2}\\ -216\beta^{2}{b_{0}}^{3}d_{1}d_{6}-360\beta^{2}{b_{0}}^{3}d_{2}d_{5}-432\beta^{2}{b_{0}}^{3}d_{3}d_{4}-432\beta^{2}{b_{0}}^{2}b_{1}{d_{3}}^{2}-240\beta^{2}{b_{0}}^{2}b_{3}{d_{2}}^{2}\\ -72\beta^{2}{b_{0}}^{2}b_{5}{d_{1}}^{2}+72\beta^{2}{b_{0}}^{2}c_{1}{d_{3}}^{2}+32\beta^{2}{b_{0}}^{2}c_{3}{d_{2}}^{2}+8\beta^{2}{b_{0}}^{2}c_{5}{d_{1}}^{2}-12\beta b_{0}{b_{2}}^{2}{d_{1}}^{2}\\ -120Q^{2}\eta^{2}c_{0}{d_{1}}^{2}d_{2}d_{5}-96Q^{2}\eta^{2}c_{1}{d_{1}}^{2}d_{2}d_{4}-144Q^{2}\eta^{2}c_{1}d_{1}{d_{2}}^{2}d_{3}-72Q^{2}\eta^{2}c_{2}{d_{1}}^{2}d_{2}d_{3}\\ +576\beta^{2}{b_{0}}^{2}c_{1}d_{1}{d_{2}}^{2}d_{3}-18Q^{2}\beta \eta^{2}b_{1}d_{1}d_{6}-30Q^{2}\beta \eta^{2}b_{1}d_{2}d_{5}-36Q^{2}\beta \eta^{2}b_{1}d_{3}d_{4}\\ +12Q^{2}\beta \eta^{2}b_{3}d_{1}d_{4}+18Q^{2}\beta \eta^{2}b_{3}d_{2}d_{3}+18Q^{2}\beta \eta^{2}b_{4}d_{1}d_{3}+18Q^{2}\beta \eta^{2}b_{5}d_{1}d_{2}\\ +60\beta b_{0}Q^{2}\eta d_{1}d_{5}+96\beta b_{0}Q^{2}\eta d_{2}d_{4}-120\beta {b_{0}}^{3}d_{1}d_{5}-192\beta {b_{0}}^{3}d_{2}d_{4}-96\beta {b_{0}}^{2}b_{2}{d_{2}}^{2}\\ -24\beta {b_{0}}^{2}b_{4}{d_{1}}^{2}-96\beta b_{0}b_{1}b_{2}d_{1}d_{2}-144Q^{2}\eta^{2}c_{0}{d_{1}}^{2}d_{3}d_{4}-192Q^{2}\eta^{2}c_{0}d_{1}{d_{2}}^{2}d_{4}\\ -216Q^{2}\eta^{2}c_{0}d_{1}d_{2}{d_{3}}^{2}+480\beta^{2}{b_{0}}^{2}c_{0}{d_{1}}^{2}d_{2}d_{5}+576\beta^{2}{b_{0}}^{2}c_{0}{d_{1}}^{2}d_{3}d_{4}+768\beta^{2}{b_{0}}^{2}c_{0}d_{1}{d_{2}}^{2}d_{4}\\ +864\beta^{2}{b_{0}}^{2}c_{0}d_{1}d_{2}{d_{3}}^{2}+384\beta^{2}{b_{0}}^{2}c_{1}{d_{1}}^{2}d_{2}d_{4}+288\beta^{2}{b_{0}}^{2}c_{2}{d_{1}}^{2}d_{2}d_{3}+144\beta^{2}b_{0}Q^{2}\eta d_{1}d_{6}\\ +240\beta^{2}b_{0}Q^{2}\eta d_{2}d_{5}+288\beta^{2}b_{0}Q^{2}\eta d_{3}d_{4}-192\beta^{3}b_{0}b_{1}b_{2}d_{1}d_{4}-288\beta^{3}b_{0}b_{1}b_{2}d_{2}d_{3}\\ -144\beta^{3}b_{0}b_{1}b_{3}d_{1}d_{3}-96\beta^{3}b_{0}b_{1}b_{4}d_{1}d_{2}-96\beta^{3}b_{0}b_{2}b_{3}d_{1}d_{2}120\beta^{3}b_{0}Q^{2}d_{1}d_{5}+192\beta^{3}b_{0}Q^{2}d_{2}d_{4}\\ -120\beta^{3}b_{0}{b_{1}}^{2}d_{1}d_{5}-192\beta^{3}b_{0}{b_{1}}^{2}d_{2}d_{4}-96\beta^{3}b_{0}b_{1}b_{3}{d_{2}}^{2}-24\beta^{3}b_{0}b_{1}b_{5}{d_{1}}^{2}-72\beta^{3}b_{0}{b_{2}}^{2}d_{1}d_{3}\\ -24\beta^{3}b_{0}b_{2}b_{4}{d_{1}}^{2}+96\beta^{2}b_{0}Q^{2}d_{1}d_{4}-288\beta^{2}b_{0}b_{1}b_{2}d_{1}d_{3}-192\beta^{2}b_{0}b_{1}b_{2}{d_{2}}^{2}-192\beta^{2}b_{0}b_{1}b_{3}d_{1}d_{2}\\ -48\beta^{2}b_{0}b_{1}b_{4}{d_{1}}^{2}-96\beta^{2}b_{0}{b_{2}}^{2}d_{1}d_{2}-48\beta^{2}b_{0}b_{2}b_{3}{d_{1}}^{2}-192\beta {b_{0}}^{2}b_{1}d_{1}d_{4}-288\beta {b_{0}}^{2}b_{1}d_{2}d_{3}\\ -144\beta {b_{0}}^{2}b_{2}d_{1}d_{3}-96\beta {b_{0}}^{2}b_{3}d_{1}d_{2}-24\beta b_{0}b_{1}b_{3}{d_{1}}^{2}-216\beta^{3}{b_{0}}^{2}b_{1}d_{1}d_{6}-360\beta^{3}{b_{0}}^{2}b_{1}d_{2}d_{5}\\ -432\beta^{3}{b_{0}}^{2}b_{1}d_{3}d_{4}-240\beta^{3}{b_{0}}^{2}b_{2}d_{1}d_{5}-384\beta^{3}{b_{0}}^{2}b_{2}d_{2}d_{4}-240\beta^{3}{b_{0}}^{2}b_{3}d_{1}d_{4}-360\beta^{3}{b_{0}}^{2}b_{3}d_{2}d_{3}\\ -216\beta^{3}{b_{0}}^{2}b_{4}d_{1}d_{3}-168\beta^{3}{b_{0}}^{2}b_{5}d_{1}d_{2}+144\beta^{2}b_{0}Q^{2}d_{2}d_{3}+54\beta b_{0}Q^{2}\eta {d_{3}}^{2}-480\beta^{2}{b_{0}}^{2}b_{1}d_{1}d_{5}\\ -192\beta^{2}b_{0}{b_{1}}^{2}d_{1}d_{4}-288\beta^{2}b_{0}{b_{1}}^{2}d_{2}d_{3}+18\beta b_{0}Q^{2}d_{1}d_{3}-768\beta^{2}-12Q^{2}\eta^{2}c_{3}{d_{1}}^{3}d_{3}\\ +96\beta^{2}{b_{0}}^{2}c_{0}{d_{1}}^{3}d_{6}+80\beta^{2}{b_{0}}^{2}c_{1}{d_{1}}^{3}d_{5}+216\beta^{2}{b_{0}}^{2}c_{1}{d_{1}}^{2}{d_{3}}^{2}+64\beta^{2}{b_{0}}^{2}c_{2}{d_{1}}^{3}d_{4}+48\beta^{2}{b_{0}}^{2}c_{3}{d_{1}}^{3}d_{3}\\ +96\beta^{2}{b_{0}}^{2}c_{3}{d_{1}}^{2}{d_{2}}^{2}+32\beta^{2}{b_{0}}^{2}c_{4}{d_{1}}^{3}d_{2}-432\beta^{2}{b_{0}}^{2}b_{2}d_{1}d_{4}-648\beta^{2}{b_{0}}^{2}b_{2}d_{2}d_{3}-360\beta^{2}{b_{0}}^{2}b_{3}d_{1}d_{3}\\ -264\beta^{2}{b_{0}}^{2}b_{4}d_{1}d_{2}+{b_{0}}^{2}b_{1}d_{2}d_{4}) \end{array}$$

$$\begin{array}{l} b_{7}=-\frac{1}{7\beta \left(Q^{2}\eta^{2}{d_{1}}^{2}-8\beta^{2}{b_{0}}^{2}{d_{1}}^{2}+Q^{2}\eta^{2}-4\beta^{2}{b_{0}}^{2}\right)}(864\beta^{2}{b_{0}}^{2}c_{1}d_{1}d_{2}{d_{3}}^{2}-60Q^{2}\beta \eta^{2}b_{1}d_{3}d_{5}\\ +10Q^{2}\beta \eta^{2}b_{3}d_{1}d_{5}+24Q^{2}\beta \eta^{2}b_{5}d_{1}d_{3}-12Q^{2}\beta \eta^{2}b_{2}d_{3}d_{4}-10Q^{2}\beta \eta^{2}b_{2}d_{2}d_{5}-48Q^{2}\beta \eta^{2}b_{1}d_{2}d_{6}\\ -28Q^{2}\beta \eta^{2}b_{1}d_{1}d_{7}+30Q^{2}\beta \eta^{2}b_{4}d_{2}d_{3}+22Q^{2}\beta \eta^{2}b_{6}d_{1}d_{2}+2304\beta^{2}{b_{0}}^{2}c_{0}d_{1}d_{2}d_{3}d_{4}\\ +128\beta^{2}{b_{0}}^{2}c_{0}{d_{4}}^{2}-16Q^{2}\eta^{2}c_{2}{d_{2}}^{4}+64\beta^{2}{b_{0}}^{2}c_{2}{d_{2}}^{4}+8\beta^{2}{b_{0}}^{2}{d_{1}}^{2}c_{6}+4\beta^{2}{b_{0}}^{2}{d_{1}}^{4}c_{6}\\ +126\beta^{2}b_{0}Q^{2}{d_{3}}^{2}-252\beta^{2}b_{0}{b_{1}}^{2}{d_{3}}^{2}-112\beta^{2}b_{0}{b_{2}}^{2}{d_{2}}^{2}-28\beta^{2}b_{0}{b_{3}}^{2}{d_{1}}^{2}384\beta^{2}{b_{0}}^{2}{d_{1}}^{2}c_{2}d_{2}d_{4}\\ +288\beta^{2}{b_{0}}^{2}{d_{1}}^{2}c_{3}d_{2}d_{3}+196\beta^{2}b_{0}Q^{2}\eta d_{1}d_{7}+336\beta^{2}b_{0}Q^{2}\eta d_{2}d_{6}+420\beta^{2}b_{0}Q^{2}\eta d_{3}d_{5}\\ -280\beta^{3}b_{0}b_{1}b_{2}d_{1}d_{5}-448\beta^{3}b_{0}b_{1}b_{2}d_{2}d_{4}-224\beta^{3}b_{0}b_{1}b_{3}d_{1}d_{4}-336\beta^{3}b_{0}b_{1}b_{3}d_{2}d_{3}\\ -168\beta^{3}b_{0}b_{1}b_{4}d_{1}d_{3}-112\beta^{3}b_{0}b_{1}b_{5}d_{1}d_{2}-168\beta^{3}b_{0}b_{2}b_{3}d_{1}d_{3}-112\beta^{3}b_{0}b_{2}b_{4}d_{1}d_{2}-168\beta {b_{0}}^{3}d_{1}d_{6}\\ -280\beta {b_{0}}^{3}d_{2}d_{5}-336\beta {b_{0}}^{3}d_{3}d_{4}-252\beta {b_{0}}^{2}b_{1}{d_{3}}^{2}-112\beta {b_{0}}^{2}b_{3}{d_{2}}^{2}-96Q^{2}\eta^{2}c_{2}{d_{1}}^{2}d_{2}d_{4}\\ -144Q^{2}\eta^{2}c_{2}d_{1}{d_{2}}^{2}d_{3}-72Q^{2}\eta^{2}c_{3}{d_{1}}^{2}d_{2}d_{3}+576\beta^{2}{b_{0}}^{2}c_{2}d_{1}{d_{2}}^{2}d_{3}-6Q^{2}\beta \eta^{2}b_{2}d_{1}d_{6}\\ +16Q^{2}\beta \eta^{2}b_{3}d_{2}d_{4}+20Q^{2}\beta \eta^{2}b_{4}d_{1}d_{4}+84\beta b_{0}Q^{2}\eta d_{1}d_{6}+140\beta b_{0}Q^{2}\eta d_{2}d_{5}+168\beta b_{0}Q^{2}\eta d_{3}d_{4}\\ -448\beta^{2}b_{0}b_{1}b_{2}d_{1}d_{4}-672\beta^{2}b_{0}b_{1}b_{2}d_{2}d_{3}-336\beta^{2}b_{0}b_{1}b_{3}d_{1}d_{3}-224\beta^{2}b_{0}b_{1}b_{4}d_{1}d_{2}\\ -224\beta^{2}b_{0}b_{2}b_{3}d_{1}d_{2}-168\beta b_{0}b_{1}b_{2}d_{1}d_{3}-112\beta b_{0}b_{1}b_{3}d_{1}d_{2}-144Q^{2}\eta^{2}c_{0}{d_{1}}^{2}d_{2}d_{6}\\ -180Q^{2}\eta^{2}c_{0}{d_{1}}^{2}d_{3}d_{5}-240Q^{2}\eta^{2}c_{0}d_{1}{d_{2}}^{2}d_{5}-576Q^{2}\eta^{2}c_{0}d_{1}d_{2}d_{3}d_{4}-120Q^{2}\eta^{2}c_{1}{d_{1}}^{2}d_{2}d_{5}\\ -144Q^{2}\eta^{2}c_{1}{d_{1}}^{2}d_{3}d_{4}-192Q^{2}\eta^{2}c_{1}d_{1}{d_{2}}^{2}d_{4}-216Q^{2}\eta^{2}c_{1}d_{1}d_{2}{d_{3}}^{2}+576\beta^{2}{b_{0}}^{2}{d_{1}}^{2}c_{0}d_{2}d_{6}\\ +720\beta^{2}{b_{0}}^{2}{d_{1}}^{2}c_{0}d_{3}d_{5}+960\beta^{2}{b_{0}}^{2}c_{0}d_{1}{d_{2}}^{2}d_{5}+480\beta^{2}{b_{0}}^{2}{d_{1}}^{2}c_{1}d_{2}d_{5}+576\beta^{2}{b_{0}}^{2}{d_{1}}^{2}c_{1}d_{3}d_{4}\\ +768\beta^{2}{b_{0}}^{2}c_{1}d_{1}{d_{2}}^{2}d_{4}-304\beta^{3}{b_{0}}^{2}b_{4}d_{1}d_{4}-320\beta^{2}{b_{0}}^{3}{d_{4}}^{2}+7Q^{2}\eta^{2}b_{6}-Q^{2}\eta^{2}c_{6}-28\beta^{2}{b_{0}}^{2}b_{6}\\ +4\beta^{2}{b_{0}}^{2}c_{6}-456\beta^{3}{b_{0}}^{2}b_{4}d_{2}d_{3}-264\beta^{3}{b_{0}}^{2}b_{5}d_{1}d_{3}-200\beta^{3}{b_{0}}^{2}b_{6}d_{1}d_{2}-648\beta^{2}{b_{0}}^{2}b_{1}d_{1}d_{6}\\ -1080\beta^{2}{b_{0}}^{2}b_{1}d_{2}d_{5}-1296\beta^{2}{b_{0}}^{2}b_{1}d_{3}d_{4}-600\beta^{2}{b_{0}}^{2}b_{2}d_{1}d_{5}-960\beta^{2}{b_{0}}^{2}b_{2}d_{2}d_{4}-528\beta^{2}{b_{0}}^{2}b_{3}d_{1}d_{4}\\ -792\beta^{2}{b_{0}}^{2}b_{3}d_{2}d_{3}-432\beta^{2}{b_{0}}^{2}b_{4}d_{1}d_{3}-312\beta^{2}{b_{0}}^{2}b_{5}d_{1}d_{2}+28\beta b_{0}Q^{2}d_{1}d_{4}+42\beta b_{0}Q^{2}d_{2}d_{3}\\ -112\beta b_{0}{b_{1}}^{2}d_{1}d_{4}-168\beta b_{0}{b_{1}}^{2}d_{2}d_{3}-112\beta b_{0}b_{1}b_{2}{d_{2}}^{2}-28\beta b_{0}b_{1}b_{4}{d_{1}}^{2}-56\beta b_{0}{b_{2}}^{2}d_{1}d_{2}\\ -28\beta b_{0}b_{2}b_{3}{d_{1}}^{2}-28\beta {b_{0}}^{2}b_{5}{d_{1}}^{2}-320\beta^{3}{b_{0}}^{2}b_{1}{d_{4}}^{2}-288\beta^{3}{b_{0}}^{2}b_{3}{d_{3}}^{2}-176\beta^{3}{b_{0}}^{2}b_{5}{d_{2}}^{2}\\ -280\beta^{2}{b_{0}}^{3}d_{1}d_{7}-480\beta^{2}{b_{0}}^{3}d_{2}d_{6}-600\beta^{2}{b_{0}}^{3}d_{3}d_{5}-540\beta^{2}{b_{0}}^{2}b_{2}{d_{3}}^{2}-288\beta^{2}{b_{0}}^{2}b_{4}{d_{2}}^{2}\\ -84\beta^{2}{b_{0}}^{2}b_{6}{d_{1}}^{2}+7Q^{2}\eta^{2}b_{6}{d_{1}}^{2}+16Q^{2}\eta^{2}b_{4}{d_{2}}^{2}+9Q^{2}\eta^{2}b_{2}{d_{3}}^{2}-32Q^{2}\eta^{2}b_{0}{d_{4}}^{2}-Q^{2}\eta^{2}c_{6}{d_{1}}^{4}\\ -2Q^{2}\eta^{2}c_{6}{d_{1}}^{2}-8Q^{2}\eta^{2}c_{4}{d_{2}}^{2}+32\beta^{2}{b_{0}}^{2}c_{4}{d_{2}}^{2}-18Q^{2}\eta^{2}c_{2}{d_{3}}^{2}+72\beta^{2}{b_{0}}^{2}c_{2}{d_{3}}^{2}-32Q^{2}\eta^{2}c_{0}{d_{4}}^{2}\\ -20Q^{2}\eta^{2}c_{2}{d_{1}}^{3}d_{5}-20Q^{2}\eta^{2}c_{2}d_{1}d_{5}+80\beta^{2}{b_{0}}^{2}c_{2}d_{1}d_{5}-96Q^{2}\eta^{2}c_{1}{d_{2}}^{3}d_{3}-32Q^{2}\eta^{2}c_{3}d_{1}{d_{2}}^{3}\\ -12Q^{2}\eta^{2}c_{4}{d_{1}}^{3}d_{3}-8Q^{2}\eta^{2}c_{5}{d_{1}}^{3}d_{2}+384\beta^{2}{b_{0}}^{2}c_{1}{d_{2}}^{3}d_{3}+128\beta^{2}{b_{0}}^{2}c_{3}d_{1}{d_{2}}^{3}-28Q^{2}\eta^{2}b_{0}d_{1}d_{7}\\ -48Q^{2}\eta^{2}b_{0}d_{2}d_{6}-6Q^{2}\eta^{2}b_{1}d_{1}d_{6}-10Q^{2}\eta^{2}b_{1}d_{2}d_{5}-12Q^{2}\eta^{2}b_{1}d_{3}d_{4}+10Q^{2}\eta^{2}b_{2}d_{1}d_{5}\\ +16Q^{2}\eta^{2}b_{2}d_{2}d_{4}+20Q^{2}\eta^{2}b_{3}d_{1}d_{4}-12Q^{2}\eta^{2}c_{4}d_{1}d_{3}-8Q^{2}\eta^{2}c_{5}d_{1}d_{2}+48\beta^{2}{b_{0}}^{2}c_{4}d_{1}d_{3}\\ +32\beta^{2}{b_{0}}^{2}c_{5}d_{1}d_{2}-280\beta {b_{0}}^{2}b_{1}d_{1}d_{5}-448\beta {b_{0}}^{2}b_{1}d_{2}d_{4}-224\beta {b_{0}}^{2}b_{2}d_{1}d_{4}+168\beta^{3}b_{0}Q^{2}d_{1}d_{6}\\ +280\beta^{3}b_{0}Q^{2}d_{2}d_{5}+336\beta^{3}b_{0}Q^{2}d_{3}d_{4}+224\beta^{2}b_{0}Q^{2}\eta {d_{4}}^{2}-168\beta^{3}b_{0}{b_{1}}^{2}d_{1}d_{6}-280\beta^{3}b_{0}{b_{1}}^{2}d_{2}d_{5}\\ -336\beta^{3}b_{0}{b_{1}}^{2}d_{3}d_{4}-252\beta^{3}b_{0}b_{1}b_{2}{d_{3}}^{2}-112\beta^{3}b_{0}b_{1}b_{4}{d_{2}}^{2}-28\beta^{3}b_{0}b_{1}b_{6}{d_{1}}^{2}-112\beta^{3}b_{0}{b_{2}}^{2}d_{1}d_{4}\\ -168\beta^{3}b_{0}{b_{2}}^{2}d_{2}d_{3}-112\beta^{3}b_{0}b_{2}b_{3}{d_{2}}^{2}-28\beta^{3}b_{0}b_{2}b_{5}{d_{1}}^{2}-56\beta^{3}b_{0}{b_{3}}^{2}d_{1}d_{2}-28\beta^{3}b_{0}b_{3}b_{4}{d_{1}}^{2}\\ +140\beta^{2}b_{0}Q^{2}d_{1}d_{5}-336\beta {b_{0}}^{2}b_{2}d_{2}d_{3}-168\beta {b_{0}}^{2}b_{3}d_{1}d_{3}-112\beta {b_{0}}^{2}b_{4}d_{1}d_{2}-96Q^{2}\eta^{2}c_{0}{d_{1}}^{2}{d_{4}}^{2}\\ -24Q^{2}\eta^{2}c_{1}{d_{1}}^{3}d_{6}-54Q^{2}\eta^{2}c_{2}{d_{1}}^{2}{d_{3}}^{2}-24Q^{2}\eta^{2}c_{4}{d_{1}}^{2}{d_{2}}^{2}+224\beta^{2}b_{0}Q^{2}d_{2}d_{4}-60Q^{2}\eta^{2}b_{0}d_{3}d_{5}\\ -60Q^{2}\eta^{2}c_{0}d_{3}d_{5}-24Q^{2}\eta^{2}c_{1}d_{1}d_{6}-40Q^{2}\eta^{2}c_{1}d_{2}d_{5}-48Q^{2}\eta^{2}c_{1}d_{3}d_{4}+240\beta^{2}{b_{0}}^{2}c_{0}d_{3}d_{5}\\ +96\beta^{2}{b_{0}}^{2}c_{1}d_{1}d_{6}+160\beta^{2}{b_{0}}^{2}c_{1}d_{2}d_{5}+192\beta^{2}{b_{0}}^{2}c_{1}d_{3}d_{4}-280\beta^{2}b_{0}{b_{1}}^{2}d_{1}d_{5}-448\beta^{2}b_{0}{b_{1}}^{2}d_{2}d_{4}\\ -224\beta^{2}b_{0}b_{1}b_{3}{d_{2}}^{2}-56\beta^{2}b_{0}b_{1}b_{5}{d_{1}}^{2}-168\beta^{2}b_{0}{b_{2}}^{2}d_{1}d_{3}-56\beta^{2}b_{0}b_{2}b_{4}{d_{1}}^{2}-28Q^{2}\eta^{2}c_{0}{d_{1}}^{3}d_{7}\\ -108Q^{2}\eta^{2}c_{0}d_{1}{d_{3}}^{3}-16Q^{2}\eta^{2}c_{3}{d_{1}}^{3}d_{4}+432\beta^{2}{b_{0}}^{2}c_{0}d_{1}{d_{3}}^{3}-32Q^{2}\beta \eta^{2}b_{1}{d_{4}}^{2}+9Q^{2}\beta \eta^{2}b_{3}{d_{3}}^{2}\\ +16Q^{2}\beta \eta^{2}b_{5}{d_{2}}^{2}+30Q^{2}\eta^{2}b_{3}d_{2}d_{3}+24Q^{2}\eta^{2}b_{4}d_{1}d_{3}+22Q^{2}\eta^{2}b_{5}d_{1}d_{2}-28Q^{2}\eta^{2}c_{0}d_{1}d_{7}\\ -48Q^{2}\eta^{2}c_{0}d_{2}d_{6}-32Q^{2}\eta^{2}c_{2}d_{2}d_{4}-16Q^{2}\eta^{2}c_{3}d_{1}d_{4}-24Q^{2}\eta^{2}c_{3}d_{2}d_{3}+112\beta^{2}{b_{0}}^{2}c_{0}d_{1}d_{7}\\ +192\beta^{2}{b_{0}}^{2}c_{0}d_{2}d_{6}+128\beta^{2}{b_{0}}^{2}c_{2}d_{2}d_{4}+64\beta^{2}{b_{0}}^{2}c_{3}d_{1}d_{4}+96\beta^{2}{b_{0}}^{2}c_{3}d_{2}d_{3}-128Q^{2}\eta^{2}c_{0}{d_{2}}^{3}d_{4}\\ -216Q^{2}\eta^{2}c_{0}{d_{2}}^{2}{d_{3}}^{2}+112\beta^{2}{b_{0}}^{2}{d_{1}}^{3}c_{0}d_{7}+384\beta^{2}{b_{0}}^{2}{d_{1}}^{2}c_{0}{d_{4}}^{2}+512\beta^{2}{b_{0}}^{2}c_{0}{d_{2}}^{3}d_{4}\\ +864\beta^{2}{b_{0}}^{2}c_{0}{d_{2}}^{2}{d_{3}}^{2}+96\beta^{2}{b_{0}}^{2}{d_{1}}^{3}c_{1}d_{6}+80\beta^{2}{b_{0}}^{2}{d_{1}}^{3}c_{2}d_{5}+216\beta^{2}{b_{0}}^{2}{d_{1}}^{2}c_{2}{d_{3}}^{2}\\ +64\beta^{2}{b_{0}}^{2}{d_{1}}^{3}c_{3}d_{4}+48\beta^{2}{b_{0}}^{2}{d_{1}}^{3}c_{4}d_{3}+96\beta^{2}{b_{0}}^{2}{d_{1}}^{2}c_{4}{d_{2}}^{2}+32\beta^{2}{b_{0}}^{2}{d_{1}}^{3}c-312\beta^{3}{b_{0}}^{2}b_{2}d_{1}d_{6}\\ -520\beta^{3}{b_{0}}^{2}b_{2}d_{2}d_{5}-624\beta^{3}{b_{0}}^{2}b_{2}d_{3}d_{4}-320\beta^{3}{b_{0}}^{2}b_{3}d_{1}d_{5}-512\beta^{3}{b_{0}}^{2}b_{3}d_{2}d_{45}d_{2}-280\beta^{3}{b_{0}}^{2}b_{1}d_{1}d_{7}\\ -480\beta^{3}{b_{0}}^{2}b_{1}d_{2}d_{6}-600\beta^{3}{d_{3}}^{3}{b_{0}}^{2}b_{1}d_{3}d_{5}) \end{array}$$

$$\begin{array}{l} b_{8}=-\frac{1}{8\beta \left(Q^{2}\eta^{2}{d_{1}}^{2}-8\beta^{2}{b_{0}}^{2}{d_{1}}^{2}+Q^{2}\eta^{2}-4\beta^{2}{b_{0}}^{2}\right)}(-432Q^{2}\eta^{2}c_{0}d_{1}{d_{3}}^{2}d_{4}-576Q^{2}\eta^{2}c_{0}{d_{2}}^{2}d_{3}d_{4}\\ -240Q^{2}\eta^{2}c_{1}d_{1}{d_{2}}^{2}d_{5}-192Q^{2}\eta^{2}c_{2}d_{1}{d_{2}}^{2}d_{4}+1536\beta^{2}{b_{0}}^{2}c_{0}d_{1}d_{2}{d_{4}}^{2}+1728\beta^{2}{b_{0}}^{2}c_{0}d_{1}{d_{3}}^{2}d_{4}\\ +2304\beta^{2}{b_{0}}^{2}c_{0}{d_{2}}^{2}d_{3}d_{4}+960\beta^{2}{b_{0}}^{2}c_{1}d_{1}{d_{2}}^{2}d_{5}+768\beta^{2}{b_{0}}^{2}c_{2}d_{1}{d_{2}}^{2}d_{4}-40Q^{2}\beta \eta^{2}b_{1}d_{1}d_{8}\\ -70Q^{2}\beta \eta^{2}b_{1}d_{2}d_{7}-90Q^{2}\beta \eta^{2}b_{1}d_{3}d_{6}-100Q^{2}\beta \eta^{2}b_{1}d_{4}d_{5}-30Q^{2}\beta \eta^{2}b_{2}d_{3}d_{5}\\ +6Q^{2}\beta \eta^{2}b_{3}d_{1}d_{6}+12Q^{2}\beta \eta^{2}b_{3}d_{3}d_{4}+20Q^{2}\beta \eta^{2}b_{4}d_{1}d_{5}-384\beta^{3}b_{0}b_{1}b_{4}d_{2}d_{3}\\ +256\beta^{2}b_{0}Q^{2}\eta d_{1}d_{8}-192\beta^{3}b_{0}b_{1}b_{5}d_{1}d_{3}-128\beta^{3}b_{0}b_{1}b_{6}d_{1}d_{2}-256\beta^{3}b_{0}b_{2}b_{3}d_{1}d_{4}\\ -384\beta^{3}b_{0}b_{2}b_{3}d_{2}d_{3}-192\beta^{3}b_{0}b_{2}b_{4}d_{1}d_{3}-128\beta^{3}b_{0}b_{2}b_{5}d_{1}d_{2}-128\beta^{3}b_{0}b_{3}b_{4}d_{1}d_{2}\\ -640\beta^{2}b_{0}b_{1}b_{2}d_{1}d_{5}-1024\beta^{2}b_{0}b_{1}b_{2}d_{2}d_{4}-512\beta^{2}b_{0}b_{1}b_{3}d_{1}d_{4}-768\beta^{2}b_{0}b_{1}b_{3}d_{2}d_{3}\\ -384\beta^{2}b_{0}b_{1}b_{4}d_{1}d_{3}-256\beta^{2}b_{0}b_{1}b_{5}d_{1}d_{2}-384\beta^{2}b_{0}b_{2}b_{3}d_{1}d_{3}-256\beta^{2}b_{0}b_{2}b_{4}d_{1}d_{2}\\ -216Q^{2}\eta^{2}c_{0}{d_{1}}^{2}d_{3}d_{6}-28Q^{2}\eta^{2}c_{1}{d_{1}}^{3}d_{7}-24Q^{2}\eta^{2}c_{2}{d_{1}}^{3}d_{6}-120Q^{2}\eta^{2}c_{2}{d_{1}}^{2}d_{2}d_{5}\\ -54Q^{2}\eta^{2}c_{3}{d_{1}}^{2}{d_{3}}^{2}+448\beta^{2}b_{0}Q^{2}\eta d_{2}d_{7}+576\beta^{2}b_{0}Q^{2}\eta d_{3}d_{6}+640\beta^{2}b_{0}Q^{2}\eta d_{4}d_{5}\\ +20Q^{2}\beta \eta^{2}b_{6}{d_{2}}^{2}-40Q^{2}\eta^{2}b_{0}d_{1}d_{8}-70Q^{2}\eta^{2}b_{0}d_{2}d_{7}-14Q^{2}\eta^{2}b_{1}d_{1}d_{7}-24Q^{2}\eta^{2}b_{1}d_{2}d_{6}\\ -30Q^{2}\eta^{2}b_{1}d_{3}d_{5}+12Q^{2}\eta^{2}b_{2}d_{3}d_{4}+20Q^{2}\eta^{2}b_{3}d_{1}d_{5}+32Q^{2}\eta^{2}b_{3}d_{2}d_{4}+28Q^{2}\eta^{2}b_{4}d_{1}d_{4}\\ -72Q^{2}\eta^{2}c_{0}d_{3}d_{6}+288\beta^{2}{b_{0}}^{2}c_{0}d_{3}d_{6}-160Q^{2}\eta^{2}c_{0}{d_{2}}^{3}d_{5}+640\beta^{2}{b_{0}}^{2}c_{0}{d_{2}}^{3}d_{5}+224\beta^{3}b_{0}Q^{2}d_{1}d_{7}\\ +384\beta^{3}b_{0}Q^{2}d_{2}d_{6}+480\beta^{3}b_{0}Q^{2}d_{3}d_{5}-224\beta^{3}b_{0}{b_{1}}^{2}d_{1}d_{7}-384\beta^{3}b_{0}{b_{1}}^{2}d_{2}d_{6}-480\beta^{3}b_{0}{b_{1}}^{2}d_{3}d_{5}\\ -288\beta^{3}b_{0}b_{1}b_{3}{d_{3}}^{2}-128\beta^{3}b_{0}b_{1}b_{5}{d_{2}}^{2}-32\beta^{3}b_{0}b_{1}b_{7}{d_{1}}^{2}-160\beta^{3}b_{0}{b_{2}}^{2}d_{1}d_{5}-384\beta {b_{0}}^{2}b_{1}d_{1}d_{6}\\ -640\beta {b_{0}}^{2}b_{1}d_{2}d_{5}-768\beta {b_{0}}^{2}b_{1}d_{3}d_{4}-320\beta {b_{0}}^{2}b_{2}d_{1}d_{5}-512\beta {b_{0}}^{2}b_{2}d_{2}d_{4}-256\beta {b_{0}}^{2}b_{3}d_{1}d_{4}\\ -384\beta {b_{0}}^{2}b_{3}d_{2}d_{3}-192\beta {b_{0}}^{2}b_{4}d_{1}d_{3}-128\beta {b_{0}}^{2}b_{5}d_{1}d_{2}+112\beta^{2}{b_{0}}^{2}{d_{1}}^{3}c_{1}d_{7}+96\beta^{2}{b_{0}}^{2}{d_{1}}^{3}c_{2}d_{6}\\ +80\beta^{2}{b_{0}}^{2}{d_{1}}^{3}c_{3}d_{5}+64\beta^{2}{b_{0}}^{2}{d_{1}}^{3}c_{4}d_{4}-368\beta^{3}{b_{0}}^{2}b_{5}d_{1}d_{4}-552\beta^{3}{b_{0}}^{2}b_{5}d_{2}d_{3}-312\beta^{3}{b_{0}}^{2}b_{6}d_{1}d_{3}\\ -232\beta^{3}{b_{0}}^{2}b_{7}d_{1}d_{2}-256\beta^{3}b_{0}{b_{2}}^{2}d_{2}d_{4}-128\beta^{3}b_{0}b_{2}b_{4}{d_{2}}^{2}-32\beta^{3}b_{0}b_{2}b_{6}{d_{1}}^{2}-96\beta^{3}b_{0}{b_{3}}^{2}d_{1}d_{3}\\ -32\beta^{3}b_{0}b_{3}b_{5}{d_{1}}^{2}-840\beta^{2}{b_{0}}^{2}b_{1}d_{1}d_{7}-1440\beta^{2}{b_{0}}^{2}b_{1}d_{2}d_{6}-160\beta b_{0}{b_{1}}^{2}d_{1}d_{5}-256\beta b_{0}{b_{1}}^{2}d_{2}d_{4}\\ -128\beta b_{0}b_{1}b_{3}{d_{2}}^{2}-32\beta b_{0}b_{1}b_{5}{d_{1}}^{2}-96\beta b_{0}{b_{2}}^{2}d_{1}d_{3}-32\beta b_{0}b_{2}b_{4}{d_{1}}^{2}+384\beta^{2}{b_{0}}^{2}{d_{1}}^{2}c_{1}{d_{4}}^{2}\\ +216\beta^{2}{b_{0}}^{2}{d_{1}}^{2}c_{3}{d_{3}}^{2}+96\beta^{2}{b_{0}}^{2}{d_{1}}^{2}c_{5}{d_{2}}^{2}+192\beta^{2}b_{0}Q^{2}d_{1}d_{6}+320\beta^{2}b_{0}Q^{2}d_{2}d_{5}+384\beta^{2}b_{0}Q^{2}d_{3}d_{4}\\ +128\beta b_{0}Q^{2}\eta {d_{4}}^{2}-384\beta^{2}b_{0}{b_{1}}^{2}d_{1}d_{6}-640\beta^{2}b_{0}{b_{1}}^{2}d_{2}d_{5}-768\beta^{2}b_{0}{b_{1}}^{2}d_{3}d_{4}-576\beta^{2}b_{0}b_{1}b_{2}{d_{3}}^{2}\\ -256\beta^{2}b_{0}b_{1}b_{4}{d_{2}}^{2}-64\beta^{2}b_{0}b_{1}b_{6}{d_{1}}^{2}-256\beta^{2}b_{0}{b_{2}}^{2}d_{1}d_{4}-384\beta^{2}b_{0}{b_{2}}^{2}d_{2}d_{3}-256\beta^{2}b_{0}b_{2}b_{3}{d_{2}}^{2}\\ -64\beta^{2}b_{0}b_{2}b_{5}{d_{1}}^{2}-128\beta^{2}b_{0}{b_{3}}^{2}d_{1}d_{2}-64\beta^{2}b_{0}b_{3}b_{4}{d_{1}}^{2}+40\beta b_{0}Q^{2}d_{1}d_{5}+64\beta b_{0}Q^{2}d_{2}d_{4}\\ -96Q^{2}\eta^{2}c_{2}{d_{2}}^{3}d_{3}-8Q^{2}\eta^{2}c_{6}{d_{1}}^{3}d_{2}+864\beta^{2}{b_{0}}^{2}{d_{1}}^{2}c_{0}d_{3}d_{6}+960\beta^{2}{b_{0}}^{2}{d_{1}}^{2}c_{0}d_{4}d_{5}\\ +576\beta^{2}{b_{0}}^{2}{d_{1}}^{2}c_{1}d_{2}d_{6}+720\beta^{2}{b_{0}}^{2}{d_{1}}^{2}c_{1}d_{3}d_{5}+480\beta^{2}{b_{0}}^{2}{d_{1}}^{2}c_{2}d_{2}d_{5}+576\beta^{2}{b_{0}}^{2}{d_{1}}^{2}c_{2}d_{3}d_{4}\\ +384\beta^{2}{b_{0}}^{2}c_{2}{d_{2}}^{3}d_{3}+288\beta^{2}{b_{0}}^{2}{d_{1}}^{2}c_{4}d_{2}d_{3}+30Q^{2}\eta^{2}b_{5}d_{1}d_{3}-56Q^{2}\eta^{2}c_{0}d_{2}d_{7}-80Q^{2}\eta^{2}c_{0}d_{4}d_{5}\\ -20Q^{2}\eta^{2}c_{3}d_{1}d_{5}-32Q^{2}\eta^{2}c_{3}d_{2}d_{4}-16Q^{2}\eta^{2}c_{4}d_{1}d_{4}+320\beta^{2}{b_{0}}^{2}c_{0}d_{4}d_{5}+80\beta^{2}{b_{0}}^{2}c_{3}d_{1}d_{5}\\ +128\beta^{2}{b_{0}}^{2}c_{3}d_{2}d_{4}+64\beta^{2}{b_{0}}^{2}c_{4}d_{1}d_{4}672\beta^{2}{b_{0}}^{2}{d_{1}}^{2}c_{0}d_{2}d_{7}+384\beta^{2}{b_{0}}^{2}{d_{1}}^{2}c_{3}d_{2}d_{4}-384\beta^{3}b_{0}b_{1}b_{2}d_{1}d_{6}\\ -640\beta^{3}b_{0}b_{1}b_{2}d_{2}d_{5}-768\beta^{3}b_{0}b_{1}b_{2}d_{3}d_{4}-320\beta^{3}b_{0}b_{1}b_{3}d_{1}d_{5}-512\beta^{3}b_{0}b_{1}b_{3}d_{2}d_{4}-256\beta^{3}b_{0}b_{1}b_{4}d_{1}d_{4}\\ +112\beta b_{0}Q^{2}\eta d_{1}d_{7}+192\beta b_{0}Q^{2}\eta d_{2}d_{6}+240\beta b_{0}Q^{2}\eta d_{3}d_{5}-256\beta b_{0}b_{1}b_{2}d_{1}d_{4}-384\beta b_{0}b_{1}b_{2}d_{2}d_{3}\\ -192\beta b_{0}b_{1}b_{3}d_{1}d_{3}-128\beta b_{0}b_{1}b_{4}d_{1}d_{2}-128\beta b_{0}b_{2}b_{3}d_{1}d_{2}-168Q^{2}\eta^{2}c_{0}{d_{1}}^{2}d_{2}d_{7}-240Q^{2}\eta^{2}c_{0}{d_{1}}^{2}d_{4}d_{5}\\ -384Q^{2}\eta^{2}c_{0}d_{1}d_{2}{d_{4}}^{2}-144Q^{2}\eta^{2}c_{1}{d_{1}}^{2}d_{2}d_{6}-180Q^{2}\eta^{2}c_{1}{d_{1}}^{2}d_{3}d_{5}-144Q^{2}\eta^{2}c_{2}{d_{1}}^{2}d_{3}d_{4}\\ -216Q^{2}\eta^{2}c_{2}d_{1}d_{2}{d_{3}}^{2}-96Q^{2}\eta^{2}c_{3}{d_{1}}^{2}d_{2}d_{4}-72Q^{2}\eta^{2}c_{4}{d_{1}}^{2}d_{2}d_{3}+864\beta^{2}{b_{0}}^{2}c_{2}d_{1}d_{2}{d_{3}}^{2}\\ -14Q^{2}\beta \eta^{2}b_{2}d_{1}d_{7}-24Q^{2}\beta \eta^{2}b_{2}d_{2}d_{6}+10Q^{2}\beta \eta^{2}b_{3}d_{2}d_{5}+42Q^{2}\beta \eta^{2}b_{5}d_{2}d_{3}+30Q^{2}\beta \eta^{2}b_{6}d_{1}d_{3}\\ +26Q^{2}\beta \eta^{2}b_{7}d_{1}d_{2}-288Q^{2}\eta^{2}c_{0}d_{1}{d_{2}}^{2}d_{6}-144Q^{2}\eta^{2}c_{3}d_{1}{d_{2}}^{2}d_{3}+1152\beta^{2}{b_{0}}^{2}c_{0}d_{1}{d_{2}}^{2}d_{6}\\ +576\beta^{2}{b_{0}}^{2}c_{3}d_{1}{d_{2}}^{2}d_{3}+32Q^{2}\beta \eta^{2}b_{4}d_{2}d_{4}+28Q^{2}\beta \eta^{2}b_{5}d_{1}d_{4}-448\beta^{3}{b_{0}}^{2}b_{2}{d_{4}}^{2}-360\beta^{3}{b_{0}}^{2}b_{4}{d_{3}}^{2}\\ -208\beta^{3}{b_{0}}^{2}b_{6}{d_{2}}^{2}-880\beta^{2}{b_{0}}^{3}d_{4}d_{5}-960\beta^{2}{b_{0}}^{2}b_{1}{d_{4}}^{2}-648\beta^{2}{b_{0}}^{2}b_{3}{d_{3}}^{2}-336\beta^{2}{b_{0}}^{2}b_{5}{d_{2}}^{2}\\ -96\beta^{2}{b_{0}}^{2}b_{7}{d_{1}}^{2}+36\beta b_{0}Q^{2}{d_{3}}^{2}-480\beta {b_{0}}^{3}d_{3}d_{5}-288\beta {b_{0}}^{2}b_{2}{d_{3}}^{2}-128\beta {b_{0}}^{2}b_{4}{d_{2}}^{2}-32\beta {b_{0}}^{2}b_{6}{d_{1}}^{2}\\ -144\beta b_{0}{b_{1}}^{2}{d_{3}}^{2}-64\beta b_{0}{b_{2}}^{2}{d_{2}}^{2}-16\beta b_{0}{b_{3}}^{2}{d_{1}}^{2}-16Q^{2}\eta^{2}c_{3}{d_{2}}^{4}-Q^{2}\eta^{2}c_{7}{d_{1}}^{4}+64\beta^{2}{b_{0}}^{2}c_{3}{d_{2}}^{4}\\ +4\beta^{2}{b_{0}}^{2}{d_{1}}^{4}c_{7}+256\beta^{3}b_{0}Q^{2}{d_{4}}^{2}-256\beta^{3}b_{0}{b_{1}}^{2}{d_{4}}^{2}-144\beta^{3}b_{0}{b_{2}}^{2}{d_{3}}^{2}-64\beta^{3}b_{0}{b_{3}}^{2}{d_{2}}^{2}-16\beta^{3}b_{0}{b_{4}}^{2}{d_{1}}^{2}\\ -16Q^{2}\eta^{2}b_{1}{d_{4}}^{2}+18Q^{2}\eta^{2}b_{3}{d_{3}}^{2}+20Q^{2}\eta^{2}b_{5}{d_{2}}^{2}-32Q^{2}\eta^{2}c_{1}{d_{4}}^{2}-18Q^{2}\eta^{2}c_{3}{d_{3}}^{2}-8Q^{2}\eta^{2}c_{5}{d_{2}}^{2}\\ -352\beta^{2}{b_{0}}^{3}d_{1}d_{8}-616\beta^{2}{b_{0}}^{3}d_{2}d_{7}-792\beta^{2}{b_{0}}^{3}d_{3}d_{6}+128\beta^{2}{b_{0}}^{2}c_{1}{d_{4}}^{2}+72\beta^{2}{b_{0}}^{2}c_{3}{d_{3}}^{2}\\ +32\beta^{2}{b_{0}}^{2}c_{5}{d_{2}}^{2}+8\beta^{2}{b_{0}}^{2}{d_{1}}^{2}c_{7}-224\beta {b_{0}}^{3}d_{1}d_{7}-384\beta {b_{0}}^{3}d_{2}d_{6}-12Q^{2}\eta^{2}c_{5}{d_{1}}^{3}d_{3}\\ -24Q^{2}\eta^{2}c_{5}{d_{1}}^{2}{d_{2}}^{2}+48\beta^{2}{b_{0}}^{2}{d_{1}}^{3}c_{5}d_{3}-16Q^{2}\beta \eta^{2}b_{2}{d_{4}}^{2}-352\beta^{3}{b_{0}}^{2}b_{1}d_{1}d_{8}-616\beta^{3}{b_{0}}^{2}b_{1}d_{2}d_{7}\\ -792\beta^{3}{b_{0}}^{2}b_{1}d_{3}d_{6}-880\beta^{3}{b_{0}}^{2}b_{1}d_{4}d_{5}-392\beta^{3}{b_{0}}^{2}b_{2}d_{1}d_{7}+26Q^{2}\eta^{2}b_{6}d_{1}d_{2}+8Q^{2}\eta^{2}b_{7}{d_{1}}^{2}\\ -32Q^{2}\eta^{2}c_{0}d_{1}d_{8}-12Q^{2}\eta^{2}c_{5}d_{1}d_{3}-8Q^{2}\eta^{2}c_{6}d_{1}d_{2}-2Q^{2}\eta^{2}c_{7}{d_{1}}^{2}-936\beta^{2}{b_{0}}^{2}b_{4}d_{2}d_{3}\\ -504\beta^{2}{b_{0}}^{2}b_{5}d_{1}d_{3}-360\beta^{2}{b_{0}}^{2}b_{6}d_{1}d_{2}+128\beta^{2}{b_{0}}^{2}c_{0}d_{1}d_{8}+224\beta^{2}{b_{0}}^{2}c_{0}d_{2}d_{7}+48\beta^{2}{b_{0}}^{2}c_{5}d_{1}d_{3}\\ +32\beta^{2}{b_{0}}^{2}c_{6}d_{1}d_{2}-720Q^{2}\eta^{2}c_{0}d_{1}d_{2}d_{3}d_{5}-16Q^{2}\eta^{2}c_{4}{d_{1}}^{3}d_{4}+128\beta^{2}{b_{0}}^{2}{d_{1}}^{3}c_{0}d_{8}+32\beta^{2}{b_{0}}^{2}{d_{1}}^{3}c_{6}d_{2}\\ -672\beta^{3}{b_{0}}^{2}b_{2}d_{2}d_{6}-840\beta^{3}{b_{0}}^{2}b_{2}d_{3}d_{5}-408\beta^{3}{b_{0}}^{2}b_{3}d_{1}d_{6}-680\beta^{3}{b_{0}}^{2}b_{3}d_{2}d_{5}-816\beta^{3}{b_{0}}^{2}b_{3}d_{3}d_{4}\\ -400\beta^{3}{b_{0}}^{2}b_{4}d_{1}d_{5}-640\beta^{3}{b_{0}}^{2}b_{4}d_{2}d_{4}-40Q^{2}\eta^{2}c_{2}d_{2}d_{5}-1800\beta^{2}{b_{0}}^{2}b_{1}d_{3}d_{5}-792\beta^{2}{b_{0}}^{2}b_{2}d_{1}d_{6}\\ -1320\beta^{2}{b_{0}}^{2}b_{2}d_{2}d_{5}-1584\beta^{2}{b_{0}}^{2}b_{2}d_{3}d_{4}-720\beta^{2}{b_{0}}^{2}b_{3}d_{1}d_{5}-1152\beta^{2}{b_{0}}^{2}b_{3}d_{2}d_{4}-624\beta^{2}{b_{0}}^{2}b_{4}d_{1}d_{4}\\ +160\beta^{2}{b_{0}}^{2}c_{2}d_{2}d_{5}-32Q^{2}\eta^{2}c_{0}{d_{1}}^{3}d_{8}-216Q^{2}\eta^{2}c_{0}d_{2}{d_{3}}^{3}-108Q^{2}\eta^{2}c_{1}d_{1}{d_{3}}^{3}-128Q^{2}\eta^{2}c_{1}{d_{2}}^{3}d_{4}\\ +864\beta^{2}{b_{0}}^{2}c_{0}d_{2}{d_{3}}^{3}+432\beta^{2}{b_{0}}^{2}c_{1}d_{1}{d_{3}}^{3}+512\beta^{2}{b_{0}}^{2}c_{1}{d_{2}}^{3}d_{4}+18Q^{2}\beta \eta^{2}b_{4}{d_{3}}^{2}-90Q^{2}\eta^{2}b_{0}d_{3}d_{6}\\ -100Q^{2}\eta^{2}b_{0}d_{4}d_{5}-28Q^{2}\eta^{2}c_{1}d_{1}d_{7}-48Q^{2}\eta^{2}c_{1}d_{2}d_{6}-60Q^{2}\eta^{2}c_{1}d_{3}d_{5}-24Q^{2}\eta^{2}c_{2}d_{1}d_{6}\\ -48Q^{2}\eta^{2}c_{2}d_{3}d_{4}+112\beta^{2}{b_{0}}^{2}c_{1}d_{1}d_{7}+192\beta^{2}{b_{0}}^{2}c_{1}d_{2}d_{6}+240\beta^{2}{b_{0}}^{2}c_{1}d_{3}d_{5}+96\beta^{2}{b_{0}}^{2}c_{2}d_{1}d_{6}\\ +192\beta^{2}{b_{0}}^{2}c_{2}d_{3}d_{4}-96Q^{2}\eta^{2}c_{1}{d_{1}}^{2}{d_{4}}^{2}-576Q^{2}\eta^{2}c_{1}d_{1}d_{2}d_{3}d_{4}-216Q^{2}\eta^{2}c_{1}{d_{2}}^{2}{d_{3}}^{2}-20Q^{2}\eta^{2}c_{3}{d_{1}}^{3}d_{5}\\ -32Q^{2}\eta^{2}c_{4}d_{1}{d_{2}}^{3}+2880\beta^{2}{b_{0}}^{2}c_{0}d_{1}d_{2}d_{3}d_{5}+2304\beta^{2}{b_{0}}^{2}c_{1}d_{1}d_{2}d_{3}d_{4}+864\beta^{2}{b_{0}}^{2}c_{1}{d_{2}}^{2}{d_{3}}^{2}\\ +128\beta^{2}{b_{0}}^{2}c_{4}d_{1}{d_{2}}^{3}+6Q^{2}\eta^{2}b_{2}d_{1}d_{6}+10Q^{2}\eta^{2}b_{2}d_{2}d_{5}+42Q^{2}\eta^{2}b_{4}d_{2}d_{3}-24Q^{2}\eta^{2}c_{4}d_{2}d_{3}\\ +96\beta^{2}{b_{0}}^{2}c_{4}d_{2}d_{3}-256\beta {b_{0}}^{3}{d_{4}}^{2}+8Q^{2}\eta^{2}b_{7}-Q^{2}\eta^{2}c_{7}-32\beta^{2}{b_{0}}^{2}b_{7}+4\beta^{2}{b_{0}}^{2}c_{7}) \end{array}$$

$$c_{1}=\frac{\beta \nu b_{1}+\nu b_{0}-c_{0}-1+\sqrt{\nu^{2}{c_{0}}^{2}-2\nu b_{0}c_{0}-2\nu c_{0}+{b_{0}}^{2}-{d_{1}}^{2}+2b_{0}+1}}{\beta }$$

$$\begin{array}{l} c_{2}=\frac{1}{2\beta \left(\beta \nu b_{1}-\beta c_{1}+\nu b_{0}-c_{0}-1\right)}(2\beta^{2}\nu^{2}b_{1}b_{2}-2\beta^{2}\nu b_{2}c_{1}+2\beta \nu^{2}b_{0}b_{2}+2\beta \nu^{2}{b_{1}}^{2}\\ -4\beta \nu b_{1}c_{1}-2\beta \nu b_{2}c_{0}+2\nu^{2}b_{0}b_{1}-\nu^{2}c_{0}c_{1}-2\beta \nu b_{2}+2\beta {c_{1}}^{2}-\nu b_{0}c_{1}-\nu b_{1}c_{0}-2\nu b_{1}+\nu c_{1}\\ -b_{0}b_{1}+2c_{0}c_{1}+2d_{1}d_{2}-b_{1}+2c_{1}) \end{array}$$

$$\begin{array}{l} c_{3}=\frac{1}{6\beta \left(\beta \nu b_{1}-\beta c_{1}+\nu b_{0}-c_{0}-1\right)}(6\beta^{2}\nu^{2}b_{1}b_{3}+4\beta^{2}\nu^{2}{b_{2}}^{2}-8\beta^{2}\nu b_{2}c_{2}-6\beta^{2}\nu b_{3}c_{1}\\ +6\beta \nu^{2}b_{0}b_{3}+14\beta \nu^{2}b_{1}b_{2}+4\beta^{2}{c_{2}}^{2}-14\beta \nu b_{1}c_{2}-14\beta \nu b_{2}c_{1}-6\beta \nu b_{3}c_{0}+6\nu^{2}b_{0}b_{2}\\ +4\nu^{2}{b_{1}}^{2}-2\nu^{2}c_{0}c_{2}-\nu^{2}{c_{1}}^{2}-6\beta \nu b_{3}+14\beta c_{1}c_{2}-4\nu b_{0}c_{2}-6\nu b_{1}c_{1}-4\nu b_{2}c_{0}-6\nu b_{2}\\ +2\nu c_{2}-2b_{0}b_{2}-{b_{1}}^{2}+6c_{0}c_{2}+4{c_{1}}^{2}+6d_{1}d_{3}+4{d_{2}}^{2}-2b_{2}+6c_{2}) \end{array}$$

$$\begin{array}{l} c_{4}=\frac{1}{4\beta \left(\beta \nu b_{1}-\beta c_{1}+\nu b_{0}-c_{0}-1\right)}(4\beta^{2}\nu^{2}b_{1}b_{4}+6\beta^{2}\nu^{2}b_{2}b_{3}-6\beta^{2}\nu b_{2}c_{3}-6\beta^{2}\nu b_{3}c_{2}\\ -4\beta^{2}\nu b_{4}c_{1}+4\beta \nu^{2}b_{0}b_{4}+10\beta \nu^{2}b_{1}b_{3}+6\beta \nu^{2}{b_{2}}^{2}+6\beta^{2}c_{2}c_{3}-10\beta \nu b_{1}c_{3}-12\beta \nu b_{2}c_{2}\\ -10\beta \nu b_{3}c_{1}-4\beta \nu b_{4}c_{0}+4\nu^{2}b_{0}b_{3}+6\nu^{2}b_{1}b_{2}-\nu^{2}c_{0}c_{3}-\nu^{2}c_{1}c_{2}-4\beta \nu b_{4}+10\beta c_{1}c_{3}\\ +6\beta {c_{2}}^{2}-3\nu b_{0}c_{3}-5\nu b_{1}c_{2}-5\nu b_{2}c_{1}-3\nu b_{3}c_{0}-4\nu b_{3}+\nu c_{3}-b_{0}b_{3}-b_{1}b_{2}+4c_{0}c_{3}+6c_{1}c_{2}\\ +4d_{1}d_{4}+6d_{2}d_{3}-b_{3}+4c_{3}) \end{array}$$

$$\begin{array}{l} c_{5}=\frac{1}{10\beta \left(\beta \nu b_{1}-\beta c_{1}+\nu b_{0}-c_{0}-1\right)}(10\beta^{2}\nu^{2}b_{1}b_{5}+16\beta^{2}\nu^{2}b_{2}b_{4}+9\beta^{2}\nu^{2}{b_{3}}^{2}-16\beta^{2}\nu b_{2}c_{4}\\ -18\beta^{2}\nu b_{3}c_{3}-16\beta^{2}\nu b_{4}c_{2}-10\beta^{2}\nu b_{5}c_{1}+10\beta \nu^{2}b_{0}b_{5}+26\beta \nu^{2}b_{1}b_{4}+34\beta \nu^{2}b_{2}b_{3}+16\beta^{2}c_{2}c_{4}\\ +9\beta^{2}{c_{3}}^{2}-26\beta \nu b_{1}c_{4}-34\beta \nu b_{2}c_{3}-34\beta \nu b_{3}c_{2}-26\beta \nu b_{4}c_{1}-10\beta \nu b_{5}c_{0}+10\nu^{2}b_{0}b_{4}\\ +16\nu^{2}b_{1}b_{3}+9\nu^{2}{b_{2}}^{2}-2\nu^{2}c_{0}c_{4}-2\nu^{2}c_{1}c_{3}-\nu^{2}{c_{2}}^{2}-10\beta \nu b_{5}+26\beta c_{1}c_{4}+34\beta c_{2}c_{3}-8\nu b_{0}c_{4}\\ -14\nu b_{1}c_{3}-16\nu b_{2}c_{2}-14\nu b_{3}c_{1}-8\nu b_{4}c_{0}-10\nu b_{4}+2\nu c_{4}-2b_{0}b_{4}-2b_{1}b_{3}-{b_{2}}^{2}+10c_{0}c_{4}\\ +16c_{1}c_{3}+9{c_{2}}^{2}+10d_{1}d_{5}+16d_{2}d_{4}+9{d_{3}}^{2}-2b_{4}+10c_{4}) \end{array}$$

$$\begin{array}{l} c_{6}=\frac{1}{6\beta \left(\beta \nu b_{1}-\beta c_{1}+\nu b_{0}-c_{0}-1\right)}(6\beta^{2}\nu^{2}b_{1}b_{6}+10\beta^{2}\nu^{2}b_{2}b_{5}+12\beta^{2}\nu^{2}b_{3}b_{4}-10\beta^{2}\nu b_{2}c_{5}\\ -12\beta^{2}\nu b_{3}c_{4}-12\beta^{2}\nu b_{4}c_{3}-10\beta^{2}\nu b_{5}c_{2}-6\beta^{2}\nu b_{6}c_{1}+6\beta \nu^{2}b_{0}b_{6}+16\beta \nu^{2}b_{1}b_{5}+22\beta \nu^{2}b_{2}b_{4}\\ +12\beta \nu^{2}{b_{3}}^{2}+10\beta^{2}c_{2}c_{5}+12\beta^{2}c_{3}c_{4}-16\beta \nu b_{1}c_{5}-22\beta \nu b_{2}c_{4}-24\beta \nu b_{3}c_{3}-22\beta \nu b_{4}c_{2}\\ -16\beta \nu b_{5}c_{1}-6\beta \nu b_{6}c_{0}+6\nu^{2}b_{0}b_{5}+10\nu^{2}b_{1}b_{4}+12\nu^{2}b_{2}b_{3}-\nu^{2}c_{0}c_{5}-\nu^{2}c_{1}c_{4}-\nu^{2}c_{2}c_{3}-6\beta \nu b_{6}\\ +16\beta c_{1}c_{5}+22\beta c_{2}c_{4}+12\beta {c_{3}}^{2}-5\nu b_{0}c_{5}-9\nu b_{1}c_{4}-11\nu b_{2}c_{3}-11\nu b_{3}c_{2}-9\nu b_{4}c_{1}-5\nu b_{5}c_{0}\\ -6\nu b_{5}+\nu c_{5}-b_{0}b_{5}-b_{1}b_{4}-b_{2}b_{3}+6c_{0}c_{5}+10c_{1}c_{4}+12c_{2}c_{3}+6d_{1}d_{6}+10d_{2}d_{5}+12d_{3}d_{4}-b_{5}+6c_{5}) \end{array}$$

$$\begin{array}{l} c_{7}=\frac{1}{14\beta \left(\beta \nu b_{1}-\beta c_{1}+\nu b_{0}-c_{0}-1\right)}(14\beta^{2}\nu^{2}b_{1}b_{7}+24\beta^{2}\nu^{2}b_{2}b_{6}+30\beta^{2}\nu^{2}b_{3}b_{5}+16\beta^{2}\nu^{2}{b_{4}}^{2}\\ -24\beta^{2}\nu b_{2}c_{6}-30\beta^{2}\nu b_{3}c_{5}-32\beta^{2}\nu b_{4}c_{4}-30\beta^{2}\nu b_{5}c_{3}-24\beta^{2}\nu b_{6}c_{2}-14\beta^{2}\nu b_{7}c_{1}+14\beta \nu^{2}b_{0}b_{7}\\ +38\beta \nu^{2}b_{1}b_{6}+54\beta \nu^{2}b_{2}b_{5}+62\beta \nu^{2}b_{3}b_{4}+24\beta^{2}c_{2}c_{6}+30\beta^{2}c_{3}c_{5}+16\beta^{2}{c_{4}}^{2}-38\beta \nu b_{1}c_{6}\\ -54\beta \nu b_{2}c_{5}-62\beta \nu b_{3}c_{4}-62\beta \nu b_{4}c_{3}-54\beta \nu b_{5}c_{2}-38\beta \nu b_{6}c_{1}-14\beta \nu b_{7}c_{0}14\nu^{2}b_{0}b_{6}+24\nu^{2}b_{1}b_{5}\\ +30\nu^{2}b_{2}b_{4}+16\nu^{2}{b_{3}}^{2}-2\nu^{2}c_{0}c_{6}-2\nu^{2}c_{1}c_{5}-2\nu^{2}c_{2}c_{4}-\nu^{2}{c_{3}}^{2}-14\beta \nu b_{7}+38\beta c_{1}c_{6}+54\beta c_{2}c_{5}\\ +62\beta c_{3}c_{4}-12\nu b_{0}c_{6}-22\nu b_{1}c_{5}-28\nu b_{2}c_{4}-30\nu b_{3}c_{3}-28\nu b_{4}c_{2}-22\nu b_{5}c_{1}-12\nu b_{6}c_{0}-14\nu b_{6}\\ +2\nu c_{6}-2b_{0}b_{6}-2b_{1}b_{5}-2b_{2}b_{4}-{b_{3}}^{2}+14c_{0}c_{6}+24c_{1}c_{5}+30c_{2}c_{4}+16{c_{3}}^{2}+14d_{1}d_{7}+24d_{2}d_{6}\\ +30d_{3}d_{5}+16{d_{4}}^{2}-2b_{6}+14c_{6}) \end{array}$$

$$\begin{array}{l} c_{8}=\frac{1}{8\beta \left(\beta \nu b_{1}-\beta c_{1}+\nu b_{0}-c_{0}-1\right)}(8\beta^{2}\nu^{2}b_{1}b_{8}+14\beta^{2}\nu^{2}b_{2}b_{7}+18\beta^{2}\nu^{2}b_{3}b_{6}+20\beta^{2}\nu^{2}b_{4}b_{5}\\ -14\beta^{2}\nu b_{2}c_{7}-18\beta^{2}\nu b_{3}c_{6}-20\beta^{2}\nu b_{4}c_{5}-20\beta^{2}\nu b_{5}c_{4}-18\beta^{2}\nu b_{6}c_{3}-14\beta^{2}\nu b_{7}c_{2}-8\beta^{2}\nu b_{8}c_{1}\\ +8\beta \nu^{2}b_{0}b_{8}+22\beta \nu^{2}b_{1}b_{7}+32\beta \nu^{2}b_{2}b_{6}+38\beta \nu^{2}b_{3}b_{5}+20\beta \nu^{2}{b_{4}}^{2}+14\beta^{2}c_{2}c_{7}+18\beta^{2}c_{3}c_{6}\\ +20\beta^{2}c_{4}c_{5}-22\beta \nu b_{1}c_{7}-32\beta \nu b_{2}c_{6}-38\beta \nu b_{3}c_{5}-40\beta \nu b_{4}c_{4}-38\beta \nu b_{5}c_{3}-32\beta \nu b_{6}c_{2}\\ -22\beta \nu b_{7}c_{1}-8\beta \nu b_{8}c_{0}+8\nu^{2}b_{0}b_{7}+14\nu^{2}b_{1}b_{6}+18\nu^{2}b_{2}b_{5}+20\nu^{2}b_{3}b_{4}-\nu^{2}c_{0}c_{7}-\nu^{2}c_{1}c_{6}\\ -\nu^{2}c_{2}c_{5}-\nu^{2}c_{3}c_{4}-8\beta \nu b_{8}+22\beta c_{1}c_{7}+32\beta c_{2}c_{6}+38\beta c_{3}c_{5}+20\beta {c_{4}}^{2}-7\nu b_{0}c_{7}-13\nu b_{1}c_{6}\\ -17\nu b_{2}c_{5}-19\nu b_{3}c_{4}-19\nu b_{4}c_{3}-17\nu b_{5}c_{2}-13\nu b_{6}c_{1}-7\nu b_{7}c_{0}-8\nu b_{7}+\nu c_{7}-b_{0}b_{7}-b_{1}b_{6}\\ -b_{2}b_{5}-b_{3}b_{4}+8c_{0}c_{7}+14c_{1}c_{6}+18c_{2}c_{5}+20c_{3}c_{4}+8d_{1}d_{8}+14d_{2}d_{7}+18d_{3}d_{6}+20d_{4}d_{5}-b_{7}+8c_{7}) \end{array}$$

$$d_{1}=-\frac{\eta Q}{\sqrt{-Q^{2}\eta^{2}+4\beta^{2}{b_{0}}^{2}}}$$

$$\begin{array}{l} d_{2}=-\frac{\beta }{d_{1}\left(Q^{2}\eta^{2}{d_{1}}^{2}-8\beta^{2}{b_{0}}^{2}{d_{1}}^{2}+Q^{2}\eta^{2}-4\beta^{2}{b_{0}}^{2}\right)}(-2b_{0}c_{0}{d_{1}}^{6}+Q^{2}\eta {d_{1}}^{4}-2{b_{0}}^{2}{d_{1}}^{4}-4b_{0}c_{0}{d_{1}}^{4}\\ +2Q^{2}\eta {d_{1}}^{2}+2b_{0}{d_{1}}^{4}-2b_{0}c_{0}{d_{1}}^{2}+Q^{2}\eta ) \end{array}$$

$$\begin{array}{l} d_{3}=-\frac{1}{3d_{1}\left(Q^{2}\eta^{2}{d_{1}}^{2}-8\beta^{2}{b_{0}}^{2}{d_{1}}^{2}+Q^{2}\eta^{2}-4\beta^{2}{b_{0}}^{2}\right)}(-16\beta b_{0}c_{0}{d_{1}}^{5}d_{2}-2\beta b_{0}c_{1}{d_{1}}^{6}+2Q^{2}\beta^{2}{d_{1}}^{4}\\ +8Q^{2}\beta \eta {d_{1}}^{3}d_{2}+2Q^{2}\eta^{2}{d_{1}}^{2}{d_{2}}^{2}-16\beta^{2}{b_{0}}^{2}{d_{1}}^{2}{d_{2}}^{2}-12\beta^{2}b_{0}b_{1}{d_{1}}^{3}d_{2}-2\beta^{2}{b_{1}}^{2}{d_{1}}^{4}+Q^{2}\eta {d_{1}}^{4}\\ -12\beta {b_{0}}^{2}{d_{1}}^{3}d_{2}-4\beta b_{0}b_{1}{d_{1}}^{4}-16\beta b_{0}c_{0}{d_{1}}^{3}d_{2}-4\beta b_{0}c_{1}{d_{1}}^{4}+4Q^{2}\beta^{2}{d_{1}}^{2}+8Q^{2}\beta \eta d_{1}d_{2}+2Q^{2}\eta^{2}{d_{2}}^{2}\\ -8\beta^{2}{b_{0}}^{2}{d_{2}}^{2}-16\beta^{2}b_{0}b_{1}d_{1}d_{2}-2\beta^{2}{b_{1}}^{2}{d_{1}}^{2}-2{b_{0}}^{2}{d_{1}}^{4}+2Q^{2}\eta {d_{1}}^{2}-16\beta {b_{0}}^{2}d_{1}d_{2}-4\beta b_{0}b_{1}{d_{1}}^{2}\\ -2\beta b_{0}c_{1}{d_{1}}^{2}+2Q^{2}\beta^{2}-2{b_{0}}^{2}{d_{1}}^{2}+Q^{2}\eta ) \end{array}$$

$$\begin{array}{l} d_{4}=-\frac{1}{6d_{1}\left(Q^{2}\eta^{2}{d_{1}}^{2}-8\beta^{2}{b_{0}}^{2}{d_{1}}^{2}+Q^{2}\eta^{2}-4\beta^{2}{b_{0}}^{2}\right)}(-24\beta b_{0}c_{0}{d_{1}}^{5}d_{3}-48\beta b_{0}c_{0}{d_{1}}^{4}{d_{2}}^{2}\\ -16\beta b_{0}c_{1}{d_{1}}^{5}d_{2}-2\beta b_{0}c_{2}{d_{1}}^{6}+12Q^{2}\beta^{2}{d_{1}}^{3}d_{2}+18Q^{2}\beta \eta {d_{1}}^{3}d_{3}+12Q^{2}\beta \eta {d_{1}}^{2}{d_{2}}^{2}\\ +9Q^{2}\eta^{2}{d_{1}}^{2}d_{2}d_{3}-72\beta^{2}{b_{0}}^{2}{d_{1}}^{2}d_{2}d_{3}-36\beta^{2}b_{0}b_{1}{d_{1}}^{3}d_{3}-24\beta^{2}b_{0}b_{1}{d_{1}}^{2}{d_{2}}^{2}-12\beta^{2}b_{0}b_{2}{d_{1}}^{3}d_{2}\\ -12\beta^{2}{b_{1}}^{2}{d_{1}}^{3}d_{2}-6\beta^{2}b_{1}b_{2}{d_{1}}^{4}+3Q^{2}\beta {d_{1}}^{4}+6Q^{2}\eta {d_{1}}^{3}d_{2}-36\beta {b_{0}}^{2}{d_{1}}^{3}d_{3}-24\beta {b_{0}}^{2}{d_{1}}^{2}{d_{2}}^{2}\\ -36\beta b_{0}b_{1}{d_{1}}^{3}d_{2}-6\beta b_{0}b_{2}{d_{1}}^{4}-24\beta b_{0}c_{0}{d_{1}}^{3}d_{3}-16\beta b_{0}c_{0}{d_{1}}^{2}{d_{2}}^{2}-16\beta b_{0}c_{1}{d_{1}}^{3}d_{2}-4\beta b_{0}c_{2}{d_{1}}^{4}\\ -6\beta {b_{1}}^{2}{d_{1}}^{4}+12Q^{2}\beta^{2}d_{1}d_{2}+18Q^{2}\beta \eta d_{1}d_{3}12Q^{2}\beta \eta {d_{2}}^{2}+9Q^{2}\eta^{2}d_{2}d_{3}-36\beta^{2}{b_{0}}^{2}d_{2}d_{3}\\ -36\beta^{2}b_{0}b_{1}d_{1}d_{3}-24\beta^{2}b_{0}b_{1}{d_{2}}^{2}-24\beta^{2}b_{0}b_{2}d_{1}d_{2}-12\beta^{2}{b_{1}}^{2}d_{1}d_{2}-6\beta^{2}b_{1}b_{2}{d_{1}}^{2}-12{b_{0}}^{2}{d_{1}}^{3}d_{2}\\ -6b_{0}b_{1}{d_{1}}^{4}+6Q^{2}\beta {d_{1}}^{2}+6Q^{2}\eta d_{1}d_{2}-36\beta {b_{0}}^{2}d_{1}d_{3}-24\beta {b_{0}}^{2}{d_{2}}^{2}-48\beta b_{0}b_{1}d_{1}d_{2}-6\beta b_{0}b_{2}{d_{1}}^{2}\\ -2\beta b_{0}c_{2}{d_{1}}^{2}-6\beta {b_{1}}^{2}{d_{1}}^{2}-12{b_{0}}^{2}d_{1}d_{2}-6b_{0}b_{1}{d_{1}}^{2}+3Q^{2}\beta ) \end{array}$$

$$\begin{array}{l} d_{5}=-\frac{1}{10d_{1}\left(Q^{2}\eta^{2}{d_{1}}^{2}-8\beta^{2}{b_{0}}^{2}{d_{1}}^{2}+Q^{2}\eta^{2}-4\beta^{2}{b_{0}}^{2}\right)}(-32\beta b_{0}c_{0}{d_{1}}^{5}d_{4}-144\beta b_{0}c_{0}{d_{1}}^{4}d_{2}d_{3}\\ -64\beta b_{0}c_{0}{d_{1}}^{3}{d_{2}}^{3}-24\beta b_{0}c_{1}{d_{1}}^{5}d_{3}-48\beta b_{0}c_{1}{d_{1}}^{4}{d_{2}}^{2}-16\beta b_{0}c_{2}{d_{1}}^{5}d_{2}-2\beta b_{0}c_{3}{d_{1}}^{6}+24Q^{2}\beta^{2}{d_{1}}^{3}d_{3}\\ +16Q^{2}\beta^{2}{d_{1}}^{2}{d_{2}}^{2}+32Q^{2}\beta \eta {d_{1}}^{3}d_{4}+48Q^{2}\beta \eta {d_{1}}^{2}d_{2}d_{3}+16Q^{2}\eta^{2}{d_{1}}^{2}d_{2}d_{4}+9Q^{2}\eta^{2}{d_{1}}^{2}{d_{3}}^{2}\\ -128\beta^{2}{b_{0}}^{2}{d_{1}}^{2}d_{2}d_{4}-72\beta^{2}{b_{0}}^{2}{d_{1}}^{2}{d_{3}}^{2}-72\beta^{2}b_{0}b_{1}{d_{1}}^{3}d_{4}-108\beta^{2}b_{0}b_{1}{d_{1}}^{2}d_{2}d_{3}-36\beta^{2}b_{0}b_{2}{d_{1}}^{3}d_{3}\\ -24\beta^{2}b_{0}b_{2}{d_{1}}^{2}{d_{2}}^{2}-12\beta^{2}b_{0}b_{3}{d_{1}}^{3}d_{2}-24\beta^{2}{b_{1}}^{2}{d_{1}}^{3}d_{3}-16\beta^{2}{b_{1}}^{2}{d_{1}}^{2}{d_{2}}^{2}-32\beta^{2}b_{1}b_{2}{d_{1}}^{3}d_{2}\\ -8\beta^{2}b_{1}b_{3}{d_{1}}^{4}-4\beta^{2}{b_{2}}^{2}{d_{1}}^{4}+16Q^{2}\beta {d_{1}}^{3}d_{2}+12Q^{2}\eta {d_{1}}^{3}d_{3}+8Q^{2}\eta {d_{1}}^{2}{d_{2}}^{2}-72\beta {b_{0}}^{2}{d_{1}}^{3}d_{4}\\ -108\beta {b_{0}}^{2}{d_{1}}^{2}d_{2}d_{3}-84\beta b_{0}b_{1}{d_{1}}^{3}d_{3}-56\beta b_{0}b_{1}{d_{1}}^{2}{d_{2}}^{2}-44\beta b_{0}b_{2}{d_{1}}^{3}d_{2}-8\beta b_{0}b_{3}{d_{1}}^{4}\\ -32\beta b_{0}c_{0}{d_{1}}^{3}d_{4}-48\beta b_{0}c_{0}{d_{1}}^{2}d_{2}d_{3}-24\beta b_{0}c_{1}{d_{1}}^{3}d_{3}-16\beta b_{0}c_{1}{d_{1}}^{2}{d_{2}}^{2}-16\beta b_{0}c_{2}{d_{1}}^{3}d_{2}\\ -4\beta b_{0}c_{3}{d_{1}}^{4}-32\beta {b_{1}}^{2}-16\beta b_{1}b_{2}{d_{1}}^{4}+24Q^{2}\beta^{2}d_{1}d_{3}+16Q^{2}\beta^{2}{d_{2}}^{2}+32Q^{2}\beta \eta d_{1}d_{4}\\ +48Q^{2}\beta \eta d_{2}d_{3}+16Q^{2}\eta^{2}d_{2}d_{4}+9Q^{2}\eta^{2}{d_{3}}^{2}+Q^{2}{d_{1}}^{4}-64\beta^{2}{b_{0}}^{2}d_{2}d_{4}-36\beta^{2}{b_{0}}^{2}{d_{3}}^{2}\\ -64\beta^{2}b_{0}b_{1}d_{1}d_{4}-96\beta^{2}b_{0}b_{1}d_{2}d_{3}-48\beta^{2}b_{0}b_{2}d_{1}d_{3}-32\beta^{2}b_{0}b_{2}{d_{2}}^{2}-32\beta^{2}b_{0}b_{3}d_{1}d_{2}\\ -24\beta^{2}{b_{1}}^{2}d_{1}d_{3}-16\beta^{2}{b_{1}}^{2}{d_{2}}^{2}-32\beta^{2}b_{1}b_{2}d_{1}d_{2}-8\beta^{2}b_{1}b_{3}{d_{1}}^{2}-4\beta^{2}{b_{2}}^{2}{d_{1}}^{2}-24{b_{0}}^{2}{d_{1}}^{3}d_{3}\\ -16{b_{0}}^{2}{d_{1}}^{2}{d_{2}}^{2}-32b_{0}b_{1}{d_{1}}^{3}d_{2}-8b_{0}b_{2}{d_{1}}^{4}-4{b_{1}}^{2}{d_{1}}^{4}+d_{2}{d_{1}}^{3}16Q^{2}\beta d_{1}d_{2}+12Q^{2}\eta d_{1}d_{3}\\ +8Q^{2}\eta {d_{2}}^{2}-64\beta {b_{0}}^{2}d_{1}d_{4}-96\beta {b_{0}}^{2}d_{2}d_{3}-96\beta b_{0}b_{1}d_{1}d_{3}-64\beta b_{0}b_{1}{d_{2}}^{2}-64\beta b_{0}b_{2}d_{1}d_{2}\\ -8\beta b_{0}b_{3}{d_{1}}^{2}-2\beta b_{0}c_{3}{d_{1}}^{2}-32\beta {b_{1}}^{2}d_{1}d_{2}-16\beta b_{1}b_{2}{d_{1}}^{2}+2Q^{2}{d_{1}}^{2}-24{b_{0}}^{2}d_{1}d_{3}-16{b_{0}}^{2}{d_{2}}^{2}\\ -32b_{0}b_{1}d_{1}d_{2}-8b_{0}b_{2}{d_{1}}^{2}-4{b_{1}}^{2}{d_{1}}^{2}+Q^{2}) \end{array}$$

$$\begin{array}{l} d_{6}=-\frac{1}{15d_{1}\left(Q^{2}\eta^{2}{d_{1}}^{2}-8\beta^{2}{b_{0}}^{2}{d_{1}}^{2}+Q^{2}\eta^{2}-4\beta^{2}{b_{0}}^{2}\right)}(-40\beta b_{0}c_{0}{d_{1}}^{5}d_{5}-192\beta b_{0}c_{0}{d_{1}}^{4}d_{2}d_{4}\\ -108\beta b_{0}c_{0}{d_{1}}^{4}{d_{3}}^{2}-288\beta b_{0}c_{0}{d_{1}}^{3}{d_{2}}^{2}d_{3}-32\beta b_{0}c_{0}{d_{1}}^{2}{d_{2}}^{4}-32\beta b_{0}c_{1}{d_{1}}^{5}d_{4}-144\beta b_{0}c_{1}{d_{1}}^{4}d_{2}d_{3}\\ -64\beta b_{0}c_{1}{d_{1}}^{3}{d_{2}}^{3}-24\beta b_{0}c_{2}{d_{1}}^{5}d_{3}-48\beta b_{0}c_{2}{d_{1}}^{4}{d_{2}}^{2}-16\beta b_{0}c_{3}{d_{1}}^{5}d_{2}-2\beta b_{0}c_{4}{d_{1}}^{6}\\ +40Q^{2}\beta^{2}{d_{1}}^{3}d_{4}+60Q^{2}\beta^{2}{d_{1}}^{2}d_{2}d_{3}+50Q^{2}\beta \eta {d_{1}}^{3}d_{5}+80Q^{2}\beta \eta {d_{1}}^{2}d_{2}d_{4}+45Q^{2}\beta \eta {d_{1}}^{2}{d_{3}}^{2}\\ +25Q^{2}\eta^{2}{d_{1}}^{2}d_{2}d_{5}+30Q^{2}\eta^{2}{d_{1}}^{2}d_{3}d_{4}-200\beta^{2}{b_{0}}^{2}{d_{1}}^{2}d_{2}d_{5}-240\beta^{2}{b_{0}}^{2}{d_{1}}^{2}d_{3}d_{4}-120\beta^{2}b_{0}b_{1}{d_{1}}^{3}d_{5}\\ -192\beta^{2}b_{0}b_{1}{d_{1}}^{2}d_{2}d_{4}-108\beta^{2}b_{0}b_{1}{d_{1}}^{2}{d_{3}}^{2}-72\beta^{2}b_{0}b_{2}{d_{1}}^{3}d_{4}-108\beta^{2}b_{0}b_{2}{d_{1}}^{2}d_{2}d_{3}-36\beta^{2}b_{0}b_{3}{d_{1}}^{3}d_{3}\\ -24\beta^{2}b_{0}b_{3}{d_{1}}^{2}{d_{2}}^{2}-12\beta^{2}b_{0}b_{4}{d_{1}}^{3}d_{2}-40\beta^{2}{b_{1}}^{2}{d_{1}}^{3}d_{4}-60\beta^{2}{b_{1}}^{2}{d_{1}}^{2}d_{2}d_{3}-60\beta^{2}b_{1}b_{2}{d_{1}}^{3}d_{3}\\ -40\beta^{2}b_{1}b_{2}{d_{1}}^{2}{d_{2}}^{2}-40\beta^{2}b_{1}b_{3}{d_{1}}^{3}d_{2}-10\beta^{2}b_{1}b_{4}{d_{1}}^{4}-20\beta^{2}{b_{2}}^{2}{d_{1}}^{3}d_{2}-10\beta^{2}b_{2}b_{3}{d_{1}}^{4}\\ +30Q^{2}\beta {d_{1}}^{3}d_{3}+20Q^{2}\beta {d_{1}}^{2}{d_{2}}^{2}+20Q^{2}\eta {d_{1}}^{3}d_{4}+30Q^{2}\eta {d_{1}}^{2}d_{2}d_{3}-120\beta {b_{0}}^{2}{d_{1}}^{3}d_{5}\\ -192\beta {b_{0}}^{2}{d_{1}}^{2}d_{2}d_{4}-108\beta {b_{0}}^{2}{d_{1}}^{2}{d_{3}}^{2}-152\beta b_{0}b_{1}{d_{1}}^{3}d_{4}-228\beta b_{0}b_{1}{d_{1}}^{2}d_{2}d_{3}-96\beta b_{0}b_{2}{d_{1}}^{3}d_{3}\\ -64\beta b_{0}b_{2}{d_{1}}^{2}{d_{2}}^{2}-52\beta b_{0}b_{3}{d_{1}}^{3}d_{2}-10\beta b_{0}b_{4}{d_{1}}^{4}-40\beta b_{0}c_{0}{d_{1}}^{3}d_{5}-64\beta b_{0}c_{0}{d_{1}}^{2}d_{2}d_{4}\\ -36\beta b_{0}c_{0}{d_{1}}^{2}{d_{3}}^{2}-32\beta b_{0}c_{1}{d_{1}}^{3}d_{4}-48\beta b_{0}c_{1}{d_{1}}^{2}d_{2}d_{3}-24\beta b_{0}c_{2}{d_{1}}^{3}d_{3}-16\beta b_{0}c_{2}{d_{1}}^{2}{d_{2}}^{2}\\ -16\beta b_{0}c_{3}{d_{1}}^{3}d_{2}-4\beta b_{0}c_{4}{d_{1}}^{4}-60\beta {b_{1}}^{2}{d_{1}}^{3}d_{3}-40\beta {b_{1}}^{2}{d_{1}}^{2}{d_{2}}^{2}-80\beta b_{1}b_{2}{d_{1}}^{3}d_{2}-20\beta b_{1}b_{3}{d_{1}}^{4}\\ -10\beta {b_{2}}^{2}{d_{1}}^{4}+40Q^{2}\beta^{2}d_{1}d_{4}+60Q^{2}\beta^{2}d_{2}d_{3}+50Q^{2}\beta \eta d_{1}d_{5}+80Q^{2}\beta \eta d_{2}d_{4}+45Q^{2}\beta \eta {d_{3}}^{2}\\ +25Q^{2}\eta^{2}d_{2}d_{5}+30Q^{2}\eta^{2}d_{3}d_{4}+5Q^{2}{d_{1}}^{3}d_{2}-100\beta^{2}{b_{0}}^{2}d_{2}d_{5}-120\beta^{2}{b_{0}}^{2}d_{3}d_{4}-100\beta^{2}b_{0}b_{1}d_{1}d_{5}\\ -160\beta^{2}b_{0}b_{1}d_{2}d_{4}-90\beta^{2}b_{0}b_{1}{d_{3}}^{2}-80\beta^{2}b_{0}b_{2}d_{1}d_{4}-120\beta^{2}b_{0}b_{2}d_{2}d_{3}-60\beta^{2}b_{0}b_{3}d_{1}d_{3}-40\beta^{2}b_{0}b_{3}{d_{2}}^{2}\\ -40\beta^{2}b_{0}b_{4}d_{1}d_{2}-40\beta^{2}{b_{1}}^{2}d_{1}d_{4}-60\beta^{2}{b_{1}}^{2}d_{2}d_{3}-60\beta^{2}b_{1}b_{2}d_{1}d_{3}-40\beta^{2}b_{1}b_{2}{d_{2}}^{2}-40\beta^{2}b_{1}b_{3}d_{1}d_{2}\\ -10\beta^{2}b_{1}b_{4}{d_{1}}^{2}-20\beta^{2}{b_{2}}^{2}d_{1}d_{2}-10\beta^{2}b_{2}b_{3}{d_{1}}^{2}-40{b_{0}}^{2}{d_{1}}^{3}d_{4}-60{b_{0}}^{2}{d_{1}}^{2}d_{2}d_{3}-60b_{0}b_{1}{d_{1}}^{3}d_{3}\\ -40b_{0}b_{1}{d_{1}}^{2}{d_{2}}^{2}-40b_{0}b_{2}{d_{1}}^{3}d_{2}-10b_{0}b_{3}{d_{1}}^{4}-20{b_{1}}^{2}{d_{1}}^{3}d_{2}-10b_{1}b_{2}{d_{1}}^{4}+30Q^{2}\beta d_{1}d_{3}+20Q^{2}\beta {d_{2}}^{2}\\ +20Q^{2}\eta d_{1}d_{4}+30Q^{2}\eta d_{2}d_{3}-100\beta {b_{0}}^{2}d_{1}d_{5}-160\beta {b_{0}}^{2}d_{2}d_{4}-90\beta {b_{0}}^{2}{d_{3}}^{2}-160\beta b_{0}b_{1}d_{1}d_{4}\\ -240\beta b_{0}b_{1}d_{2}d_{3}-120\beta b_{0}b_{2}d_{1}d_{3}-80\beta b_{0}b_{2}{d_{2}}^{2}-80\beta b_{0}b_{3}d_{1}d_{2}-10\beta b_{0}b_{4}{d_{1}}^{2}-2\beta b_{0}c_{4}{d_{1}}^{2}\\ -60\beta {b_{1}}^{2}d_{1}d_{3}-40\beta {b_{1}}^{2}{d_{2}}^{2}-80\beta b_{1}b_{2}d_{1}d_{2}-20\beta b_{1}b_{3}{d_{1}}^{2}-10\beta {b_{2}}^{2}{d_{1}}^{2}+5Q^{2}d_{1}d_{2}-40{b_{0}}^{2}d_{1}d_{4}\\ -60{b_{0}}^{2}d_{2}d_{3}-60b_{0}b_{1}d_{1}d_{3}-40b_{0}b_{1}{d_{2}}^{2}-40b_{0}b_{2}d_{1}d_{2}-10b_{0}b_{3}{d_{1}}^{2}-20{b_{1}}^{2}d_{1}d_{2}-10b_{1}b_{2}{d_{1}}^{2}) \end{array}$$

$$\begin{array}{l} d_{7}=-\frac{1}{21d_{1}\left(Q^{2}\eta^{2}{d_{1}}^{2}-8\beta^{2}{b_{0}}^{2}{d_{1}}^{2}+Q^{2}\eta^{2}-4\beta^{2}{b_{0}}^{2}\right)}(-48\beta b_{0}c_{0}{d_{1}}^{5}d_{6}-40\beta b_{0}c_{1}{d_{1}}^{5}d_{5}\\ -108\beta b_{0}c_{1}{d_{1}}^{4}{d_{3}}^{2}-32\beta b_{0}c_{1}{d_{1}}^{2}{d_{2}}^{4}-96\beta b_{1}b_{2}{d_{1}}^{2}{d_{2}}^{2}-96\beta b_{1}b_{3}{d_{1}}^{3}d_{2}+54Q^{2}\beta^{2}{d_{3}}^{2}\\ +24Q^{2}\eta^{2}{d_{4}}^{2}-96\beta^{2}{b_{0}}^{2}{d_{4}}^{2}-54\beta^{2}{b_{1}}^{2}{d_{3}}^{2}-48\beta^{2}b_{1}b_{4}d_{1}d_{2}-12\beta^{2}b_{1}b_{5}{d_{1}}^{2}-36\beta^{2}{b_{2}}^{2}d_{1}d_{3}\\ -24\beta^{2}{b_{2}}^{2}{d_{2}}^{2}-48\beta^{2}b_{2}b_{3}d_{1}d_{2}-12\beta^{2}b_{2}b_{4}{d_{1}}^{2}-144b_{0}b_{1}{d_{1}}^{2}d_{2}d_{3}+27Q^{2}\eta {d_{3}}^{2}-192\beta b_{0}b_{2}d_{1}d_{4}\\ -12\beta b_{0}b_{5}{d_{1}}^{2}-96\beta {b_{1}}^{2}d_{1}d_{4}-144\beta b_{1}b_{2}d_{1}d_{3}+6Q^{2}{d_{2}}^{2}-54{b_{0}}^{2}{d_{3}}^{2}-48b_{1}b_{2}d_{1}d_{2}\\ -32\beta b_{0}c_{2}{d_{1}}^{5}d_{4}-64\beta b_{0}c_{2}{d_{1}}^{3}{d_{2}}^{3}-24\beta b_{0}c_{3}{d_{1}}^{5}d_{3}-48\beta b_{0}c_{3}{d_{1}}^{4}{d_{2}}^{2}-16\beta b_{0}c_{4}{d_{1}}^{5}d_{2}\\ +96Q^{2}\beta^{2}{d_{1}}^{2}d_{2}d_{4}+72Q^{2}\beta \eta {d_{1}}^{3}d_{6}+36Q^{2}\eta^{2}{d_{1}}^{2}d_{2}d_{6}+45Q^{2}\eta^{2}{d_{1}}^{2}d_{3}d_{5}-288\beta^{2}{b_{0}}^{2}{d_{1}}^{2}d_{2}d_{6}\\ -360\beta^{2}{b_{0}}^{2}{d_{1}}^{2}d_{3}d_{5}-180\beta^{2}b_{0}b_{1}{d_{1}}^{3}d_{6}-120\beta^{2}b_{0}b_{2}{d_{1}}^{3}d_{5}-108\beta^{2}b_{0}b_{2}{d_{1}}^{2}{d_{3}}^{2}-48\beta b_{0}c_{0}{d_{1}}^{3}d_{6}\\ -40\beta b_{0}c_{1}{d_{1}}^{3}d_{5}-36\beta b_{0}c_{1}{d_{1}}^{2}{d_{3}}^{2}-32\beta b_{0}c_{2}{d_{1}}^{3}d_{4}-24\beta b_{0}c_{3}{d_{1}}^{3}d_{3}-16\beta b_{0}c_{3}{d_{1}}^{2}{d_{2}}^{2}\\ -16\beta b_{0}c_{4}{d_{1}}^{3}d_{2}-72\beta^{2}b_{0}b_{3}{d_{1}}^{3}d_{4}-36\beta^{2}b_{0}b_{4}{d_{1}}^{3}d_{3}-24\beta^{2}b_{0}b_{4}{d_{1}}^{2}{d_{2}}^{2}-12\beta^{2}b_{0}b_{5}{d_{1}}^{3}d_{2}\\ -96\beta^{2}{b_{1}}^{2}{d_{1}}^{2}d_{2}d_{4}-96\beta^{2}b_{1}b_{2}{d_{1}}^{3}d_{4}-72\beta^{2}b_{1}b_{3}{d_{1}}^{3}d_{3}-48\beta^{2}b_{1}b_{3}{d_{1}}^{2}{d_{2}}^{2}-48\beta^{2}b_{1}b_{4}{d_{1}}^{3}d_{2}\\ -48\beta^{2}b_{2}b_{3}{d_{1}}^{3}d_{2}+72Q^{2}\beta {d_{1}}^{2}d_{2}d_{3}+48Q^{2}\eta {d_{1}}^{2}d_{2}d_{4}-300\beta {b_{0}}^{2}{d_{1}}^{2}d_{2}d_{5}-360\beta {b_{0}}^{2}{d_{1}}^{2}d_{3}d_{4}\\ -240\beta b_{0}b_{1}{d_{1}}^{3}d_{5}-216\beta b_{0}b_{1}{d_{1}}^{2}{d_{3}}^{2}-168\beta b_{0}b_{2}{d_{1}}^{3}d_{4}-108\beta b_{0}b_{3}{d_{1}}^{3}d_{3}-72\beta b_{0}b_{3}{d_{1}}^{2}{d_{2}}^{2}\\ -60\beta b_{0}b_{4}{d_{1}}^{3}d_{2}-144\beta {b_{1}}^{2}{d_{1}}^{2}d_{2}d_{3}60Q^{2}\beta^{2}{d_{1}}^{3}d_{5}+54Q^{2}\beta^{2}{d_{1}}^{2}{d_{3}}^{2}+24Q^{2}\eta^{2}{d_{1}}^{2}{d_{4}}^{2}\\ -192\beta^{2}{b_{0}}^{2}{d_{1}}^{2}{d_{4}}^{2}-60\beta^{2}{b_{1}}^{2}{d_{1}}^{3}d_{5}-54\beta^{2}{b_{1}}^{2}{d_{1}}^{2}{d_{3}}^{2}-12\beta^{2}b_{1}b_{5}{d_{1}}^{4}-36\beta^{2}{b_{2}}^{2}{d_{1}}^{3}d_{3}\\ -24\beta^{2}{b_{2}}^{2}{d_{1}}^{2}{d_{2}}^{2}-12\beta^{2}b_{2}b_{4}{d_{1}}^{4}+48Q^{2}\beta {d_{1}}^{3}d_{4}+30Q^{2}\eta {d_{1}}^{3}d_{5}-144\beta b_{1}b_{2}{d_{1}}^{3}d_{3}+60Q^{2}\beta^{2}d_{1}d_{5}\\ +96Q^{2}\beta^{2}d_{2}d_{4}-72\beta^{2}b_{0}b_{4}d_{1}d_{3}-96\beta^{2}b_{1}b_{2}d_{1}d_{4}-6\beta^{2}{b_{3}}^{2}{d_{1}}^{2}+48Q^{2}\beta d_{1}d_{4}+72Q^{2}\beta d_{2}d_{3}\\ +30Q^{2}\eta d_{1}d_{5}+48Q^{2}\eta d_{2}d_{4}-216\beta b_{0}b_{1}{d_{3}}^{2}-24{b_{1}}^{2}{d_{2}}^{2}-6{b_{2}}^{2}{d_{1}}^{2}-6\beta^{2}{b_{3}}^{2}{d_{1}}^{4}+27Q^{2}\eta {d_{1}}^{2}{d_{3}}^{2}\\ -180\beta {b_{0}}^{2}{d_{1}}^{3}d_{6}-12\beta b_{0}b_{5}{d_{1}}^{4}-96\beta {b_{1}}^{2}{d_{1}}^{3}d_{4}-24\beta b_{1}b_{4}{d_{1}}^{4}-48\beta {b_{2}}^{2}{d_{1}}^{3}d_{2}-24\beta b_{2}b_{3}{d_{1}}^{4}\\ +9Q^{2}{d_{1}}^{3}d_{3}+6Q^{2}{d_{1}}^{2}{d_{2}}^{2}-120\beta^{2}b_{0}b_{2}d_{1}d_{5}-192\beta^{2}b_{0}b_{2}d_{2}d_{4}-48\beta^{2}b_{0}b_{5}d_{1}d_{2}-60{b_{0}}^{2}{d_{1}}^{3}d_{5}\\ -54{b_{0}}^{2}{d_{1}}^{2}{d_{3}}^{2}-12b_{0}b_{4}{d_{1}}^{4}-36{b_{1}}^{2}{d_{1}}^{3}d_{3}-24{b_{1}}^{2}{d_{1}}^{2}{d_{2}}^{2}-12b_{1}b_{3}{d_{1}}^{4}-240\beta b_{0}b_{1}d_{1}d_{5}\\ -96\beta b_{0}b_{4}d_{1}d_{2}-96\beta b_{1}b_{2}{d_{2}}^{2}-96\beta b_{1}b_{3}d_{1}d_{2}-24\beta b_{1}b_{4}{d_{1}}^{2}-48\beta {b_{2}}^{2}d_{1}d_{2}-144b_{0}b_{1}d_{2}d_{3}\\ -2\beta b_{0}c_{5}{d_{1}}^{6}+120Q^{2}\beta \eta {d_{1}}^{2}d_{2}d_{5}+144Q^{2}\beta \eta {d_{1}}^{2}d_{3}d_{4}-300\beta^{2}b_{0}b_{1}{d_{1}}^{2}d_{2}d_{5}-4\beta b_{0}c_{5}{d_{1}}^{4}\\ +36Q^{2}\eta^{2}d_{2}d_{6}+45Q^{2}\eta^{2}d_{3}d_{5}-144\beta^{2}{b_{0}}^{2}d_{2}d_{6}-180\beta^{2}{b_{0}}^{2}d_{3}d_{5}-108\beta^{2}b_{0}b_{2}{d_{3}}^{2}-144\beta^{2}b_{1}b_{2}d_{2}d_{3}\\ -96{b_{0}}^{2}{d_{1}}^{2}d_{2}d_{4}-96b_{0}b_{1}{d_{1}}^{3}d_{4}-72b_{0}b_{2}{d_{1}}^{3}d_{3}-48b_{0}b_{2}{d_{1}}^{2}{d_{2}}^{2}-48b_{0}b_{3}{d_{1}}^{3}d_{2}-48b_{1}b_{2}{d_{1}}^{3}d_{2}\\ -144\beta {b_{0}}^{2}d_{1}d_{6}-240\beta {b_{0}}^{2}d_{2}d_{5}-288\beta {b_{0}}^{2}d_{3}d_{4}-144\beta b_{0}b_{3}d_{1}d_{3}-2\beta b_{0}c_{5}{d_{1}}^{2}-72b_{0}b_{2}d_{1}d_{3}\\ -240\beta b_{0}c_{0}{d_{1}}^{4}d_{2}d_{5}-288\beta b_{0}c_{0}{d_{1}}^{4}d_{3}d_{4}-384\beta b_{0}c_{0}{d_{1}}^{3}{d_{2}}^{2}d_{4}-432\beta b_{0}c_{0}{d_{1}}^{3}d_{2}{d_{3}}^{2}\\ -192\beta b_{0}c_{0}{d_{1}}^{2}{d_{2}}^{3}d_{3}-192\beta b_{0}c_{1}{d_{1}}^{4}d_{2}d_{4}-288\beta b_{0}c_{1}{d_{1}}^{3}{d_{2}}^{2}d_{3}-144\beta b_{0}c_{2}{d_{1}}^{4}d_{2}d_{3}\\ -360\beta^{2}b_{0}b_{1}{d_{1}}^{2}d_{3}d_{4}-192\beta^{2}b_{0}b_{2}{d_{1}}^{2}d_{2}d_{4}-108\beta^{2}b_{0}b_{3}{d_{1}}^{2}d_{2}d_{3}-144\beta^{2}b_{1}b_{2}{d_{1}}^{2}d_{2}d_{3}\\ -384\beta b_{0}b_{1}{d_{1}}^{2}d_{2}d_{4}-252\beta b_{0}b_{2}{d_{1}}^{2}d_{2}d_{3}-80\beta b_{0}c_{0}{d_{1}}^{2}d_{2}d_{5}-96\beta b_{0}c_{0}{d_{1}}^{2}d_{3}d_{4}-64\beta b_{0}c_{1}{d_{1}}^{2}d_{2}d_{4}\\ -48\beta b_{0}c_{2}{d_{1}}^{2}d_{2}d_{3}72Q^{2}\beta \eta d_{1}d_{6}+120Q^{2}\beta \eta d_{2}d_{5}+144Q^{2}\beta \eta d_{3}d_{4}-96\beta^{2}b_{0}b_{3}d_{1}d_{4}\\ -144\beta^{2}b_{0}b_{3}d_{2}d_{3}-48\beta^{2}b_{0}b_{4}{d_{2}}^{2}-60\beta^{2}{b_{1}}^{2}d_{1}d_{5}-96\beta^{2}{b_{1}}^{2}d_{2}d_{4}-72\beta^{2}b_{1}b_{3}d_{1}d_{3}-48\beta^{2}b_{1}b_{3}{d_{2}}^{2}\\ -6{b_{2}}^{2}{d_{1}}^{4}-384\beta b_{0}b_{1}d_{2}d_{4}-288\beta b_{0}b_{2}d_{2}d_{3}-144\beta {b_{1}}^{2}d_{2}d_{3}-24\beta b_{2}b_{3}{d_{1}}^{2}-96b_{0}b_{1}d_{1}d_{4}\\ -48b_{0}b_{3}d_{1}d_{2}-12b_{1}b_{3}{d_{1}}^{2}-144\beta^{2}b_{0}b_{1}d_{1}d_{6}-240\beta^{2}b_{0}b_{1}d_{2}d_{5}-288\beta^{2}b_{0}b_{1}d_{3}d_{4}-96\beta b_{0}b_{3}{d_{2}}^{2}\\ +9Q^{2}d_{1}d_{3}-60{b_{0}}^{2}d_{1}d_{5}-96{b_{0}}^{2}d_{2}d_{4}-48b_{0}b_{2}{d_{2}}^{2}-12b_{0}b_{4}{d_{1}}^{2}-36{b_{1}}^{2}d_{1}d_{3}) \end{array}$$

$$\begin{array}{l} d_{8}=-\frac{1}{28d_{1}\left(Q^{2}\eta^{2}{d_{1}}^{2}-8\beta^{2}{b_{0}}^{2}{d_{1}}^{2}+Q^{2}\eta^{2}-4\beta^{2}{b_{0}}^{2}\right)}(-192\beta b_{0}{d_{1}}^{2}c_{1}{d_{2}}^{3}d_{3}-192\beta b_{0}{d_{1}}^{4}c_{2}d_{2}d_{4}\\ +168Q^{2}\beta \eta {d_{1}}^{2}d_{2}d_{6}+210Q^{2}\beta \eta {d_{1}}^{2}d_{3}d_{5}-432\beta^{2}b_{0}b_{1}{d_{1}}^{2}d_{2}d_{6}-540\beta^{2}b_{0}b_{1}{d_{1}}^{2}d_{3}d_{5}\\ -300\beta^{2}b_{0}b_{2}{d_{1}}^{2}d_{2}d_{5}-360\beta^{2}b_{0}b_{2}{d_{1}}^{2}d_{3}d_{4}-192\beta^{2}b_{0}b_{3}{d_{1}}^{2}d_{2}d_{4}-108\beta^{2}b_{0}b_{4}{d_{1}}^{2}d_{2}d_{3}\\ -224\beta^{2}b_{1}b_{2}{d_{1}}^{2}d_{2}d_{4}-168\beta^{2}b_{1}b_{3}{d_{1}}^{2}d_{2}d_{3}-580\beta b_{0}b_{1}{d_{1}}^{2}d_{2}d_{5}-696\beta b_{0}b_{1}{d_{1}}^{2}d_{3}d_{4}\\ -416\beta b_{0}b_{2}{d_{1}}^{2}d_{2}d_{4}-276\beta b_{0}b_{3}{d_{1}}^{2}d_{2}d_{3}-96\beta b_{0}{d_{1}}^{2}c_{1}d_{3}d_{4}-336\beta b_{1}b_{2}{d_{1}}^{2}d_{2}d_{3}\\ -288\beta b_{0}{d_{1}}^{4}c_{0}d_{2}d_{6}-360\beta b_{0}{d_{1}}^{4}c_{0}d_{3}d_{5}-480\beta b_{0}{d_{1}}^{3}c_{0}{d_{2}}^{2}d_{5}-1152\beta b_{0}{d_{1}}^{3}c_{0}d_{2}d_{3}d_{4}\\ -240\beta b_{0}{d_{1}}^{4}c_{1}d_{2}d_{5}-288\beta b_{0}{d_{1}}^{4}c_{1}d_{3}d_{4}-384\beta b_{0}{d_{1}}^{3}c_{1}{d_{2}}^{2}d_{4}-432\beta b_{0}{d_{1}}^{3}c_{1}d_{2}{d_{3}}^{2}\\ -288\beta b_{0}{d_{1}}^{3}c_{2}{d_{2}}^{2}d_{3}-144\beta b_{0}{d_{1}}^{4}c_{3}d_{2}d_{3}-120\beta b_{0}{d_{1}}^{2}c_{0}d_{3}d_{5}-80\beta b_{0}{d_{1}}^{2}c_{1}d_{2}d_{5}\\ -126\beta^{2}b_{1}b_{2}{d_{3}}^{2}-56\beta^{2}b_{1}b_{4}{d_{2}}^{2}-14\beta^{2}b_{1}b_{6}{d_{1}}^{2}-56\beta^{2}{b_{2}}^{2}d_{1}d_{4}-84\beta^{2}{b_{2}}^{2}d_{2}d_{3}\\ -56\beta^{2}b_{2}b_{3}{d_{2}}^{2}-14\beta^{2}b_{2}b_{5}{d_{1}}^{2}-28\beta^{2}{b_{3}}^{2}d_{1}d_{2}-14\beta^{2}b_{3}b_{4}{d_{1}}^{2}+70Q^{2}\beta d_{1}d_{5}\\ +112Q^{2}\beta d_{2}d_{4}+42Q^{2}\eta d_{1}d_{6}+70Q^{2}\eta d_{2}d_{5}+84Q^{2}\eta d_{3}d_{4}-196\beta {b_{0}}^{2}d_{1}d_{7}-336\beta {b_{0}}^{2}d_{2}d_{6}\\ -420\beta {b_{0}}^{2}d_{3}d_{5}-252\beta b_{0}b_{2}{d_{3}}^{2}-112\beta b_{0}b_{4}{d_{2}}^{2}-14\beta b_{0}b_{6}{d_{1}}^{2}-140\beta {b_{1}}^{2}d_{1}d_{5}-224\beta {b_{1}}^{2}d_{2}d_{4}\\ -112\beta b_{1}b_{3}{d_{2}}^{2}-28\beta b_{1}b_{5}{d_{1}}^{2}-84\beta {b_{2}}^{2}d_{1}d_{3}-28\beta b_{2}b_{4}{d_{1}}^{2}-140b_{0}b_{1}d_{1}d_{5}-224b_{0}b_{1}d_{2}d_{4}\\ -112b_{0}b_{2}d_{1}d_{4}-168b_{0}b_{2}d_{2}d_{3}-84b_{0}b_{3}d_{1}d_{3}-56b_{0}b_{4}d_{1}d_{2}-84b_{1}b_{2}d_{1}d_{3}-256\beta b_{0}{d_{1}}^{2}c_{0}{d_{2}}^{3}d_{4}\\ -432\beta b_{0}{d_{1}}^{2}c_{0}{d_{2}}^{2}{d_{3}}^{2}-96\beta b_{0}{d_{1}}^{2}c_{0}d_{2}d_{6}-64\beta b_{0}{d_{1}}^{2}c_{2}d_{2}d_{4}-48\beta b_{0}{d_{1}}^{2}c_{3}d_{2}d_{3}-14\beta {b_{3}}^{2}{d_{1}}^{4}\\ -336\beta b_{0}b_{1}d_{1}d_{6}-560\beta b_{0}b_{1}d_{2}d_{5}-672\beta b_{0}b_{1}d_{3}d_{4}-280\beta b_{0}b_{2}d_{1}d_{5}-448\beta b_{0}b_{2}d_{2}d_{4}\\ -224\beta b_{0}b_{3}d_{1}d_{4}-336\beta b_{0}b_{3}d_{2}d_{3}-168\beta b_{0}b_{4}d_{1}d_{3}-112\beta b_{0}b_{5}d_{1}d_{2}-224\beta b_{1}b_{2}d_{1}d_{4}\\ -336\beta b_{1}b_{2}d_{2}d_{3}-168\beta b_{1}b_{3}d_{1}d_{3}-112\beta b_{1}b_{4}d_{1}d_{2}-112\beta b_{2}b_{3}d_{1}d_{2}-56b_{1}b_{3}d_{1}d_{2}\\ -40\beta b_{0}{d_{1}}^{3}c_{2}d_{5}-16\beta b_{0}{d_{1}}^{2}c_{4}{d_{2}}^{2}+84Q^{2}\beta^{2}d_{1}d_{6}+140Q^{2}\beta^{2}d_{2}d_{5}+168Q^{2}\beta^{2}d_{3}d_{4}\\ +112Q^{2}\beta \eta {d_{4}}^{2}+49Q^{2}\eta^{2}d_{2}d_{7}+63Q^{2}\eta^{2}d_{3}d_{6}+70Q^{2}\eta^{2}d_{4}d_{5}+14Q^{2}{d_{1}}^{3}d_{4}-196\beta^{2}{b_{0}}^{2}d_{2}d_{7}\\ -252\beta^{2}{b_{0}}^{2}d_{3}d_{6}-280\beta^{2}{b_{0}}^{2}d_{4}d_{5}-224\beta^{2}b_{0}b_{1}{d_{4}}^{2}-126\beta^{2}b_{0}b_{3}{d_{3}}^{2}-56\beta^{2}b_{0}b_{5}{d_{2}}^{2}\\ -84\beta^{2}{b_{1}}^{2}d_{1}d_{6}-140\beta^{2}{b_{1}}^{2}d_{2}d_{5}-168\beta^{2}{b_{1}}^{2}d_{3}d_{4}-84{b_{0}}^{2}{d_{1}}^{3}d_{6}-14b_{0}b_{5}{d_{1}}^{4}-56{b_{1}}^{2}{d_{1}}^{3}d_{4}\\ -14b_{1}b_{4}{d_{1}}^{4}-28{b_{2}}^{2}{d_{1}}^{3}d_{2}-14b_{2}b_{3}{d_{1}}^{4}-56\beta b_{0}{d_{1}}^{5}c_{0}d_{7}-192\beta b_{0}{d_{1}}^{4}c_{0}{d_{4}}^{2}-216\beta b_{0}{d_{1}}^{3}c_{0}{d_{3}}^{3}\\ -48\beta b_{0}{d_{1}}^{5}c_{1}d_{6}-40\beta b_{0}{d_{1}}^{5}c_{2}d_{5}-108\beta b_{0}{d_{1}}^{4}c_{2}{d_{3}}^{2}-32\beta b_{0}{d_{1}}^{2}c_{2}{d_{2}}^{4}-32\beta b_{0}{d_{1}}^{5}c_{3}d_{4}\\ -64\beta b_{0}{d_{1}}^{3}c_{3}{d_{2}}^{3}-24\beta b_{0}{d_{1}}^{5}c_{4}d_{3}-48\beta b_{0}{d_{1}}^{4}c_{4}{d_{2}}^{2}-16\beta b_{0}{d_{1}}^{5}c_{5}d_{2}+140Q^{2}\beta^{2}{d_{1}}^{2}d_{2}d_{5}\\ +168Q^{2}\beta^{2}{d_{1}}^{2}d_{3}d_{4}+98Q^{2}\beta \eta {d_{1}}^{3}d_{7}-56\beta b_{0}{d_{1}}^{3}c_{0}d_{7}-64\beta b_{0}{d_{1}}^{2}c_{0}{d_{4}}^{2}-48\beta b_{0}{d_{1}}^{3}c_{1}d_{6}\\ -36\beta b_{0}{d_{1}}^{2}c_{2}{d_{3}}^{2}-32\beta b_{0}{d_{1}}^{3}c_{3}d_{4}-24\beta b_{0}{d_{1}}^{3}c_{4}d_{3}-16\beta b_{0}{d_{1}}^{3}c_{5}d_{2}112Q^{2}\beta \eta {d_{1}}^{2}{d_{4}}^{2}\\ +49Q^{2}\eta^{2}{d_{1}}^{2}d_{2}d_{7}+63Q^{2}\eta^{2}{d_{1}}^{2}d_{3}d_{6}+70Q^{2}\eta^{2}{d_{1}}^{2}d_{4}d_{5}-392\beta^{2}{b_{0}}^{2}{d_{1}}^{2}d_{2}d_{7}-504\beta^{2}{b_{0}}^{2}{d_{1}}^{2}d_{3}d_{6}\\ -560\beta^{2}{b_{0}}^{2}{d_{1}}^{2}d_{4}d_{5}-252\beta^{2}b_{0}b_{1}{d_{1}}^{3}d_{7}-288\beta^{2}b_{0}b_{1}{d_{1}}^{2}{d_{4}}^{2}-180\beta^{2}b_{0}b_{2}{d_{1}}^{3}d_{6}-120\beta^{2}b_{0}b_{3}{d_{1}}^{3}d_{5}\\ -108\beta^{2}b_{0}b_{3}{d_{1}}^{2}{d_{3}}^{2}-72\beta^{2}b_{0}b_{4}{d_{1}}^{3}d_{4}-36\beta^{2}b_{0}b_{5}{d_{1}}^{3}d_{3}-24\beta^{2}b_{0}b_{5}{d_{1}}^{2}{d_{2}}^{2}-12\beta^{2}b_{0}b_{6}{d_{1}}^{3}d_{2}\\ -140\beta^{2}{b_{1}}^{2}{d_{1}}^{2}d_{2}d_{5}-168\beta^{2}{b_{1}}^{2}{d_{1}}^{2}d_{3}d_{4}-140\beta^{2}b_{1}b_{2}{d_{1}}^{3}d_{5}-126\beta^{2}b_{1}b_{2}{d_{1}}^{2}{d_{3}}^{2}-112\beta^{2}b_{1}b_{3}{d_{1}}^{3}d_{4}\\ -84\beta^{2}b_{1}b_{4}{d_{1}}^{3}d_{3}-56\beta^{2}b_{1}b_{4}{d_{1}}^{2}{d_{2}}^{2}-56\beta^{2}b_{1}b_{5}{d_{1}}^{3}d_{2}-84\beta^{2}{b_{2}}^{2}{d_{1}}^{2}d_{2}d_{3}-84\beta^{2}b_{2}b_{3}{d_{1}}^{3}d_{3}\\ -56\beta^{2}b_{2}b_{3}{d_{1}}^{2}{d_{2}}^{2}-56\beta^{2}b_{2}b_{4}{d_{1}}^{3}d_{2}+112Q^{2}\beta {d_{1}}^{2}d_{2}d_{4}+70Q^{2}\eta {d_{1}}^{2}d_{2}d_{5}+84Q^{2}\eta {d_{1}}^{2}d_{3}d_{4}\\ -432\beta {b_{0}}^{2}{d_{1}}^{2}d_{2}d_{6}-540\beta {b_{0}}^{2}{d_{1}}^{2}d_{3}d_{5}-348\beta b_{0}b_{1}{d_{1}}^{3}d_{6}-260\beta b_{0}b_{2}{d_{1}}^{3}d_{5}-234\beta b_{0}b_{2}{d_{1}}^{2}{d_{3}}^{2}\\ -184\beta b_{0}b_{3}{d_{1}}^{3}d_{4}-120\beta b_{0}b_{4}{d_{1}}^{3}d_{3}-80\beta b_{0}b_{4}{d_{1}}^{2}{d_{2}}^{2}-68\beta b_{0}b_{5}{d_{1}}^{3}d_{2}-224\beta {b_{1}}^{2}{d_{1}}^{2}d_{2}d_{4}\\ -2\beta b_{0}{d_{1}}^{6}c_{6}+84Q^{2}\beta^{2}{d_{1}}^{3}d_{6}-4\beta b_{0}{d_{1}}^{4}c_{6}-224\beta b_{1}b_{2}{d_{1}}^{3}d_{4}-168\beta b_{1}b_{3}{d_{1}}^{3}d_{3}-112\beta b_{1}b_{3}{d_{1}}^{2}{d_{2}}^{2}\\ -112\beta b_{1}b_{4}{d_{1}}^{3}d_{2}-112\beta b_{2}b_{3}{d_{1}}^{3}d_{2}+21Q^{2}{d_{1}}^{2}d_{2}d_{3}-140{b_{0}}^{2}{d_{1}}^{2}d_{2}d_{5}-168{b_{0}}^{2}{d_{1}}^{2}d_{3}d_{4}\\ -140b_{0}b_{1}{d_{1}}^{3}d_{5}-224b_{0}b_{1}{d_{1}}^{2}d_{2}d_{4}-126b_{0}b_{1}{d_{1}}^{2}{d_{3}}^{2}-112b_{0}b_{2}{d_{1}}^{3}d_{4}-168b_{0}b_{2}{d_{1}}^{2}d_{2}d_{3}\\ -84b_{0}b_{3}{d_{1}}^{3}d_{3}-56b_{0}b_{3}{d_{1}}^{2}{d_{2}}^{2}-56b_{0}b_{4}{d_{1}}^{3}d_{2}-84{b_{1}}^{2}{d_{1}}^{2}d_{2}d_{3}-84b_{1}b_{2}{d_{1}}^{3}d_{3}-56b_{1}b_{2}{d_{1}}^{2}{d_{2}}^{2}\\ -56b_{1}b_{3}{d_{1}}^{3}d_{2}-2\beta b_{0}{d_{1}}^{2}c_{6}-84\beta^{2}{b_{1}}^{2}{d_{1}}^{3}d_{6}-14\beta^{2}b_{1}b_{6}{d_{1}}^{4}-56\beta^{2}{b_{2}}^{2}{d_{1}}^{3}d_{4}-14\beta^{2}b_{2}b_{5}{d_{1}}^{4}\\ -28\beta^{2}{b_{3}}^{2}{d_{1}}^{3}d_{2}-14\beta^{2}b_{3}b_{4}{d_{1}}^{4}+70Q^{2}\beta {d_{1}}^{3}d_{5}+63Q^{2}\beta {d_{1}}^{2}{d_{3}}^{2}+42Q^{2}\eta {d_{1}}^{3}d_{6}-252\beta {b_{0}}^{2}{d_{1}}^{3}d_{7}\\ -288\beta {b_{0}}^{2}{d_{1}}^{2}{d_{4}}^{2}-14\beta b_{0}b_{6}{d_{1}}^{4}-140\beta {b_{1}}^{2}{d_{1}}^{3}d_{5}-126\beta {b_{1}}^{2}{d_{1}}^{2}{d_{3}}^{2}-28\beta b_{1}b_{5}{d_{1}}^{4}-84\beta {b_{2}}^{2}{d_{1}}^{3}d_{3}\\ -56\beta {b_{2}}^{2}{d_{1}}^{2}{d_{2}}^{2}-28\beta b_{2}b_{4}{d_{1}}^{4}+98Q^{2}\beta \eta d_{1}d_{7}+168Q^{2}\beta \eta d_{2}d_{6}+210Q^{2}\beta \eta d_{3}d_{5}-196\beta^{2}b_{0}b_{1}d_{1}d_{7}\\ -336\beta^{2}b_{0}b_{1}d_{2}d_{6}-420\beta^{2}b_{0}b_{1}d_{3}d_{5}-168\beta^{2}b_{0}b_{2}d_{1}d_{6}-280\beta^{2}b_{0}b_{2}d_{2}d_{5}-336\beta^{2}b_{0}b_{2}d_{3}d_{4}\\ -140\beta^{2}b_{0}b_{3}d_{1}d_{5}-224\beta^{2}b_{0}b_{3}d_{2}d_{4}-112\beta^{2}b_{0}b_{4}d_{1}d_{4}-168\beta^{2}b_{0}b_{4}d_{2}d_{3}-84\beta^{2}b_{0}b_{5}d_{1}d_{3}\\ -56\beta^{2}b_{0}b_{6}d_{1}d_{2}-140\beta^{2}b_{1}b_{2}d_{1}d_{5}-224\beta^{2}b_{1}b_{2}d_{2}d_{4}-112\beta^{2}b_{1}b_{3}d_{1}d_{4}-168\beta^{2}b_{1}b_{3}d_{2}d_{3}\\ -84\beta^{2}b_{1}b_{4}d_{1}d_{3}-56\beta^{2}b_{1}b_{5}d_{1}d_{2}-84\beta^{2}b_{2}b_{3}d_{1}d_{3}-56\beta^{2}b_{2}b_{4}d_{1}d_{2}-224\beta {b_{0}}^{2}{d_{4}}^{2}-14\beta {b_{3}}^{2}{d_{1}}^{2}\\ +14Q^{2}d_{1}d_{4}+21Q^{2}d_{2}d_{3}-140{b_{0}}^{2}d_{2}d_{5}63Q^{2}\beta {d_{3}}^{2}-126\beta {b_{1}}^{2}{d_{3}}^{2}-56\beta {b_{2}}^{2}{d_{2}}^{2}-84{b_{0}}^{2}d_{1}d_{6}\\ -168{b_{0}}^{2}d_{3}d_{4}-126b_{0}b_{1}{d_{3}}^{2}-56b_{0}b_{3}{d_{2}}^{2}-14b_{0}b_{5}{d_{1}}^{2}-56{b_{1}}^{2}d_{1}d_{4}-84{b_{1}}^{2}d_{2}d_{3}-56b_{1}b_{2}{d_{2}}^{2}\\ -14b_{1}b_{4}{d_{1}}^{2}-28{b_{2}}^{2}d_{1}d_{2}-14b_{2}b_{3}{d_{1}}^{2}) \end{array}$$
